# Evolutionary change in the construction of the nursery environment when parents are prevented from caring for their young directly

**DOI:** 10.1073/pnas.2102450118

**Published:** 2021-11-24

**Authors:** Ana Duarte, Darren Rebar, Allysa C. Hallett, Benjamin J. M. Jarrett, Rebecca M. Kilner

**Affiliations:** ^a^College of Life and Environmental Sciences, University of Exeter, Penryn TR10 9FE, United Kingdom;; ^b^Department of Biological Sciences, Emporia State University, Emporia, KS 66801;; ^c^Department of Biology, Lund University, 223 62 Lund, Sweden;; ^d^Department of Zoology, University of Cambridge, Cambridge CB2 3EJ, United Kingdom

**Keywords:** parental care, extended phenotype, local adaptation, burying beetle, experimental evolution

## Abstract

Parental care can be partitioned into traits that involve direct engagement with offspring and traits that are expressed as an extended phenotype and influence the developmental environment, such as constructing a nursery. Here, we use experimental evolution to test whether parents can evolve modifications in nursery construction when they are experimentally prevented from supplying care directly to offspring. We exposed replicate experimental populations of burying beetles (*Nicrophorus vespilloides*) to different regimes of posthatching care by allowing larvae to develop in the presence (Full Care) or absence of parents (No Care). After only 13 generations of experimental evolution, we found an adaptive evolutionary increase in the pace at which parents in the No Care populations converted a dead body into a carrion nest for larvae. Cross-fostering experiments further revealed that No Care larvae performed better on a carrion nest prepared by No Care parents than did Full Care larvae. We conclude that parents construct the nursery environment in relation to their effectiveness at supplying care directly, after offspring are born. When direct care is prevented entirely, they evolve to make compensatory adjustments to the nursery in which their young will develop. The rapid evolutionary change observed in our experiments suggests there is considerable standing genetic variation for parental care traits in natural burying beetle populations—for reasons that remain unclear.

## Significance

Parents can care for offspring directly by giving them food or warmth, for example, or they can help them without direct contact via an extended phenotype by manipulating the nursery environment in which offspring develop. Using an experimental evolution approach, we prevented parents from directly supplying care to their offspring and observed how the extended care phenotype and offspring traits evolved in response. We found that depriving offspring of direct care caused rapid adaptive change in the construction of the nursery environment, which rescued offspring from otherwise poor developmental conditions. Overall, offspring tended to perform better when transplanted to a nursery environment constructed by parents of their own lineage, suggesting that offspring adapt to the evolved extended parental phenotype.

Parental care encompasses all parental traits that enhance offspring fitness and that have evolved for this purpose (1). Direct forms of care have been analyzed extensively in previous work. They involve parents engaging directly with their young by defending their offspring from attack, for example, or by brooding them when they are cold or feeding them (2). Yet, parental care can also take the form of an extended phenotype. Before their offspring even exist, parents can manipulate the nursery environment in which their future young will develop by carefully choosing the territory within which the nursery is sited, by constructing a nursery or nest, and by stockpiling it with food for the newly hatched offspring (3). In some species, such as dung beetles, beewolves, skates, and jacky dragons, parents and their offspring never meet again after egg laying. Nevertheless, the extended parental care phenotype in these species endures to influence offspring fitness (4–7).

Here, we are interested in the evolutionary relationship between the extent of direct care and the extended parental care phenotype and how that, in turn, influences the evolution of offspring traits. Each form of care is understood to generate a fitness benefit for the offspring, usually at some fitness cost to the parent that supplies it (1, 2). Any existing fitness costs limit the supply of care, but the relative benefits derived from each form of care presumably determine the relative level of investment in each of them. If the relative fitness benefits derived from direct care suddenly decline, for example, then we might expect a corresponding adaptive increase in the extended parental care phenotype to compensate for any loss in fitness experienced by the offspring. Previous studies have produced correlational evidence that is consistent with this possibility (e.g., refs. 8–10). Furthermore, recent work has investigated whether such compensatory changes can be induced via phenotypic plasticity within the lifetime of an individual (11, 12). However, we are unaware of any work that has considered how changes in one form of care cause evolutionary change in other forms of care or how that could causally influence the evolution of offspring traits.

To address this question, we took advantage of the natural variation in parental care found in the burying beetle *Nicrophorus vespilloides*, which comprises both direct care and an extended parental care phenotype. Burying beetles use small dead vertebrates, such as mice or small birds, to rear their larvae (13, 14). The extended parental care phenotype is expressed when parents transform the carcass into an edible nest. They scissor off the fur or feathers, roll it into a ball, cover the flesh with antimicrobial exudates, and bury it in a shallow grave (14–16). Eggs are laid in the soil surrounding the carcass, and when larvae hatch, they crawl to the carcass. Parents assist the offspring in colonizing the carcass by biting small holes in the flesh, which are used by larvae to penetrate the carcass. Parents may stay to supply their offspring with direct care, which involves defending them and feeding them via oral trophallaxis (17). Larvae can also feed themselves and can survive without any posthatching care (17, 18). Approximately 1 wk after hatching, larvae disperse to pupate in the soil. Parental presence during larval development increases larval survival (19), yet the duration of posthatching parental care is highly variable, with a range spanning from no posthatching care at all to the whole period of larval development (13, 20–22). Thus, the extent of direct parental care experienced by burying beetle larvae in early life is highly variable.

We used experimental evolution to investigate how a change in the supply of direct parental care affects the evolution of an extended parental care phenotype: that is, construction of the nursery environment through the conversion of the carcass into an edible nest. Both types of care have been shown to improve offspring survival (19, 23) and incur life span costs in burying beetles (24–26). We established experimental populations that evolved either with “Full Care” (FC; i.e., direct care plus extended parental care) or “No Care” (NC; i.e., only extended parental care but no direct contact with parents).

We have reported some of the outcomes of this experimental evolution work previously. We found that preventing direct posthatching care in experimental NC populations for generation after generation initially resulted in lower breeding success and larval survival (27). However, this was followed by a rapid increase in fitness in subsequent generations so that FC and NC populations had similar measures of fitness by generation 13 (27). We have also investigated how the evolution of larval traits, such as their morphology (22) and social interactions on the carcass (18, 28), contributed to this recovery in fitness in the NC populations. Here, we focus more on the evolution of parental traits in the NC populations by examining how changes in the parental extended phenotype of carcass preparation promote and interact with offspring fitness in the absence of direct care.

To disentangle the fitness consequences of changes in the parental traits from changes in larval traits, we cross-fostered larvae within and between FC and NC after multiple generations of experimental evolution (18, 22, 27, 29). By measuring correlates of larval fitness in the absence of direct care, we further determined whether evolved change in the extended parental care phenotype compensated for the loss of direct parental care (in our laboratory environment). Our results demonstrate that there is rapid adaptive evolution of the extended parental care phenotype when parents are prevented from interacting with their offspring and that offspring adapt rapidly to this changed nursery environment.

## Methods

### Experimentally Evolving Populations.

The experimental populations were founded from four wild populations of *N. vespilloides* collected in Cambridgeshire, United Kingdom (Byron’s Pool, Gamlingay Woods, Overhall Grove, and Waresley Woods) in the summer of 2014. Further details of these wild populations are given in refs. 22 and 28. The populations were interbred to create a genetically diverse stock laboratory population from which the experimentally evolving populations could be derived. This allowed us to avoid potential confounding effects of inbreeding depression, which is masked by direct parental care (30). Two selective regimes were established, one with full posthatching parental care, FC, and the other without any posthatching parental care, NC. Two independent replicates (hereafter referred to as block 1 and block 2) of the FC and NC regimes were maintained, with block 2 breeding a week after block 1.

At each generation, males and females were paired within each experimental population, excluding sibling and cousin pairings. Each pair was placed in a breeding box (17 × 12 × 6 cm) half filled with moistened compost, and a small, thawed dead mouse (8 to 14 g, obtained frozen from LiveFoods Direct) was placed on top of the soil. Breeding boxes were kept in dark cabinets to simulate natural underground conditions. In the FC populations, a minimum of 30 pairs of unrelated beetles were bred at each generation. Parents were allowed to remain in the box throughout larval development and so, were able to provide posthatching care. In the NC populations, we set up a minimum of 50 pairs each generation to compensate for the increased number of failed broods (27), and both parents were removed from the breeding box 53 h after pairing, before larvae started hatching. This allowed parents sufficient time to convert the mouse body into a carrion nest and for females to lay eggs in the surrounding soil, but it deprived larvae of any posthatching care (31, 32).

Eight days after pairing, dispersing larvae were placed into individual cells (2 × 2 × 2 cm) in an eclosion box (10 × 10 × 2 cm), with one brood per eclosion box. They were covered with moistened peat and left undisturbed to pupate. Newly eclosed adults were then removed and housed individually until breeding a minimum of 17 d after eclosion. All adult beetles were fed raw ground beef twice a week.

In generation 11, we created a third type of experimental population (from here on, known as Nm—standing for No Care, maternal) from each of the two replicates of the NC population. The two replicate Nm populations were bred in parallel with each of the two FC and NC replicates. The Nm population passed through one generation of full posthatching parental care to eliminate potential maternal and/or other environmental effects in the NC population. To create each Nm population, we thus followed the same protocol as for the FC populations and bred an additional 30 pairs of unrelated beetles from each NC replicate population. The Nm populations were discontinued after the experimental analysis described below.

In generation 13 of the FC and NC populations (corresponding to generation 1 of Nm), we collected newly eclosed adults from all three populations. We housed all beetles individually in plastic boxes (12 × 8 × 2 cm) until pairing for the experiment described below.

### Cross-Fostering Design.

We randomly selected males and females from each population to set up 450 pairs (75 pairs per population per block) and placed them in a breeding box (17 × 12 × 6 cm) with a 10- to 12-g thawed mouse carcass. After 53 h, before any larvae hatched, we removed the parents and measured the parent’s pronotum width, which is standardly used as a proxy for adult body size. As a measure of reproductive investment, we counted the number of eggs visible on the bottom of the breeding boxes, which is a noninvasive accurate method for deducing clutch size (33). Eggs were left in situ, in the soil in the breeding boxes, to hatch into larvae.

Before the eggs hatched, carcasses were swapped between breeding boxes to create a fully factorial 3 × 3 experimental design, such that larvae from each experimental population were allowed to develop on carcasses prepared by adults either from the larvae’s own natal population or from the other two experimental populations (*SI Appendix*, Fig. S1). Larvae were, therefore, always unrelated to the adults that had prepared the carcass whereupon they developed. Furthermore, since the adults were removed, no broods received any direct parental care. After 8 d, we counted and weighed surviving larvae to derive correlates of offspring fitness on the different carcasses.

Using this experimental design, we were able to separate contributions to larval fitness due to the extended parental care phenotype from any contributions to larval fitness made by larval traits (22, 28).

### The Extended Parental Care Phenotype: Nursery Construction Traits.

Burying beetles typically roll the denuded flesh of the carcass into a ball to create a nest for their larvae. The first measure we made of the nursery environment was the roundness of the carrion nest. Although no correlation between development on a rounder carcass and larval mass at dispersal has yet been found, rounder carcasses correlate negatively with paternal life span, indicating a cost to nest construction (25), and are also less hospitable to rival blowfly larvae (34). Burying beetles also smear costly antimicrobial exudates on the carcass surface (35, 36), which improves larval survival (23). A rounder carcass would minimize the surface area to volume ratio, thus potentially reducing defense costs and the possibility of carcass desiccation. This could be particularly advantageous to offspring in the absence of posthatching parental care.

We measured carcass roundness 53 h after pairing adults and presenting them with a dead mouse, following methods described in ref. 25. Briefly, we took two photographs of each carcass from perpendicular positions 30 cm away with identical cameras and settings (Fujifilm av200). When visible, we digitally removed the mouse’s tail from all photos with GIMP (v. 2.8.16; The GIMP Development Team; https://www.gimp.org/), as the tail strongly influences roundness estimates. We then calculated carcass roundness with a custom-written script (*SI Appendix*) in ImageJ (1.49v, Wayne Rasband; NIH; https://imagej.nih.gov/ij). By applying this process to a ping-pong ball, we established that a perfect sphere has a roundness score of 0.9. We, therefore, adjusted subsequent measures of roundness by dividing by 0.9, meaning that a value of 1.0 intuitively equaled a sphere.

Second, we recorded whether small holes were visible on the surface of the carcass at 53 h after pairing. There is individual variation in the timing of these holes. In previous work, we found that only 26% of wild-caught *N. vespilloides*, breeding in laboratory conditions, made a visible hole in the surface of the carcass before larval hatching (22). In the absence of posthatching care, the presence of a hole in the carcass is critical for larval survival (19). We hypothesized that NC populations might make these entrance holes earlier than FC populations. Some of these data, namely the presence of a hole in the carcass in FC and NC populations at 53 h after pairing, were published in ref. 22. Here, we present a different analysis of these data, including examining the fitness consequences of the presence of a hole for different experimental populations, as well as data regarding Nm populations.

Third, we measured antimicrobial activity in male and female anal exudates at 53 h after pairing. Adults deposit these exudates all over the carrion nest during carcass preparation. The presence of these exudates improves larval survival (23) and affects the composition of bacterial communities growing on prepared carcasses (16, 37). Lytic activity is heritable in *N. vespilloides* (38), with significant positive maternal effects, and therefore, it is feasible that selection could act to increase it in NC and Nm populations, where parents cannot maintain the carcass after larval hatching.

Burying beetles readily produce a red–brown liquid when gently tapped on the back of the abdomen. However, in some cases, individuals did not produce exudates. The total numbers of successfully sampled individuals were 398 males (131 FC, 130 NC, and 137 Nm) and 401 females (133 FC, 129 NC, and 139 Nm). We collected exudates with Pasteur pipettes, stored them in 1.5-mL Eppendorf tubes, and kept them frozen at −20 °C until further analysis. Lytic activity was measured in an automated plate reader (Biotek ELx808) by a microplate turbidity assay that quantifies the degradation rate of bacterial cell walls (adapted from ref. 23). Briefly, we diluted exudates 25-fold in potassium phosphate buffer (pH 6.4, 0.02 M). We added 10 µL of diluted exudates per well to 96-well microtiter plates filled with 100 µL per well of a 1.3-mg mL^−1^ suspension of lyophilized *Micrococcus luteus* (Sigma-Aldrich) in potassium phosphate buffer. Samples were initially incubated in the plate reader at 25 °C for 30 s with continuous shaking. Absorbance at 450 nm was measured every 10 min for 60 min, with continuous shaking for 10 min at 25 °C between measurements. We calculated lytic activity as the percentage change in absorbance relative to control wells, with 10 µL of potassium phosphate buffer and 100 µL of *M. luteus* suspension. We report here results for change in absorbance at 450 nm after 60 min.

### Statistical Analysis.

All analyses were performed using the statistical program R version 4.1.1 (39). Mixed effects models were performed with the package “lme4” version 1.1-27.1 (40). Seventeen breeding pairs were removed from the analysis either because a parent died or was damaged before adult removal (FC = 2, NC = 3, Nm = 3) or because no eggs were observed in the box upon adult removal (FC = 2, NC = 5, Nm = 2). Therefore, 433 breeding pairs were included in the analysis (FC = 146, NC = 142, Nm = 145).

#### Analysis of carcass preparation traits.

A further seven breeding pairs were removed from the analysis of carcass preparation traits because we could not measure pronotum width for both parents, and body size is an important explanatory variable for carcass roundness and lytic activity. Therefore, 426 breeding pairs were included in the analysis of carcass preparation traits (FC = 143, NC = 140, Nm = 143). To analyze the presence of a hole in the carcass, we initially fitted a generalized linear mixed model (GLMM) with a binomial distribution and a population per block random effect to account for variation between the independent replicates due to founder effects and asynchronous maintenance. No variance was explained by the random effect, and therefore, we used a generalized linear model (GLM) with a binomial distribution to analyze the presence of a hole. Carcass roundness and lytic activity were analyzed with linear mixed models. Lytic activity was log transformed to ensure that model residuals met the assumptions of normality for regression. In these and subsequent models with mixed effects, we included a population per block random effect.

#### Analysis of reproductive investment and brood performance.

To compare brood performance between experimental populations, we analyzed breeding success (i.e., survival of at least one larva to dispersal), brood size, and brood mass. Offspring mass is a known correlate of fitness in burying beetles (19, 26). Brood success was initially analyzed with a binomial GLMM. Again, the random effect population per block did not explain any variance, and we, therefore, used a binomial GLM to model brood success. Brood size and brood mass were analyzed with linear mixed models (LMMs). We removed brood failures (i.e., no larvae survived to dispersal) from the analysis of brood size and brood mass. To evaluate the relative contribution of parental and larval traits on larval survival to dispersal, we fitted a linear regression model on brood size, with the number of eggs as a covariate. We then used regression residuals as a measure of offspring survival from egg laying to larval dispersal. We included the residuals as the response variable in a LMM with population, carcass type (i.e., population that prepared the carcass), presence of a hole, female size, and carcass roundness as explanatory factors/covariates. Post hoc comparisons were performed with the package “emmeans” version 1.6.3 in R (41).

#### Model selection.

Model selection in all analyses was performed by comparing nested models with the Akaike Information Criterion (AIC) and ANOVA (42). Here, we report the minimal adequate models, where nonsignificant terms (*P* > 0.05, as reported by ANOVA of nested models) were dropped when this resulted in a decrease of AIC by two units. The significance of interaction terms was determined by performing ANOVAs on nested models (with and without the interaction terms). To validate models, we inspected residuals of all minimal adequate models. Female and male sizes, as well as carcass mass, were included as covariates in all the initial models. For LMMs, we used Satterthwaite’s approximation to calculate degrees of freedom and *P* values with the package “lmerTest” version 3.1-3 (43). To check whether approximating degrees of freedom alters statistical results, we ran all minimal adequate models with and without random effects. All models maintained the same qualitative results, and most variables showed *P* values and effect sizes of the same magnitude whether we included or excluded random effects. Only in the models for brood mass and offspring mortality did we find lower *P* values when the random effect population per block was excluded (reported in *Results*).

## Results

### Evolution of the Nursery Environment.

We found significant differences in the extended parental care phenotype across the different populations we sampled. Carcasses prepared by NC and Nm beetles were twice as likely to have a hole as carcasses prepared by FC beetles (Table 1). At the time of the removal of parents (53 h after pairing), the percentages of carcasses with a visible hole were 30% in FC carcasses, 61% in NC carcasses, and 59% in Nm carcasses.

**Table 1.** Summary of minimal adequate models of carcass preparation traits in FC, NC, and Nm populations

Term	Estimate	SEM	DF	*z* value	*t* value	*P* value
Presence of a hole: Binomial GLM						
Intercept	−5.30	2.53		−2.10		0.04*
Population N	1.36	0.27		4.94		<0.001***
Population Nm	1.22	0.25		4.83		<0.001***
Male size	0.04	0.25		0.15		0.88
Female size	0.30	0.29		1.04		0.30
Carcass mass	0.23	0.13		1.75		0.08
Carcass roundness: GLMM						
Intercept	−0.34	0.19	314.69		−1.77	0.08
Population N	0.96	0.23	343.04		4.26	<0.001***
Population NM	1.23	0.23	349.51		5.24	<0.001***
Male size	0.14	0.02	339.35		6.30	<0.001***
Female size	0.10	0.03	373.68		3.78	<0.001***
Carcass mass	0.001	0.01	415.03		0.27	0.79
* *Interaction *Χ*^2^						
Population × male size	20.79		2			<0.001***
Population × female size	9.85		2			0.007**
Female lytic activity (log transformed): GLMM						
Intercept	2.15	0.57	307.15		3.80	<0.001***
Female size	0.41	0.10	340.57		3.88	<0.001***
Partner’s log lytic activity	0.27	0.06	322.37		4.93	<0.001***
Male lytic activity (log transformed): GLMM						
Intercept	3.53	0.47	312.08		7.59	<0.001***
Male size	0.17	0.09	333.87		2.00	0.047*
Partner’s log lytic activity	0.20	0.05	355.90		4.38	<0.001***

We present *z* values for binomial generalized linear model (GLM) and *T* values for generalized linear mixed models (GLMMs). Degrees of freedom (DF) for GLMMs calculated with Satterthwaite's approximation. Asterisks denote statistical significance. *P < 0.05; **P < 0.01; ***P < 0.001.

**Fig. 1.**
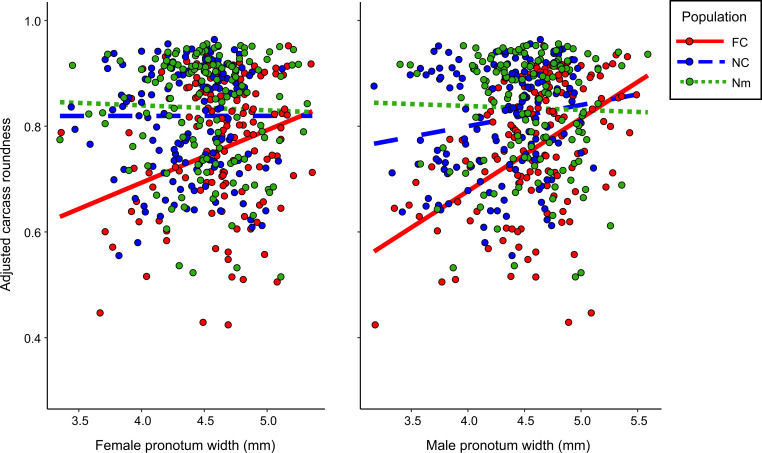
The predicted partial effects of the experimental population of origin and female (*Left*) and male (*Right*) pronotum width on carcass roundness. Each data point represents a carcass prepared by a pair of beetles (*n* = 426). Lines represent adjusted carcass roundness values predicted by a linear mixed model.

Carcasses prepared by NC and Nm beetles were also rounder (Table 1) (overall effect of population: *Χ*^2^ = 25.80, *P* = 2.5 × 10^−6^). There was a complex, significant interaction between population type and both female and male body sizes (Fig. 1 and Table 1). In FC lines, carcass roundness was positively associated with both male and female sizes. In NC and Nm lines, the slope of the relationship between carcass roundness and body size was significantly shallower than in FC lines, with beetles across a range of sizes producing similarly well-rounded carcasses.

Lytic activity in anal exudates at 53 h was similar across the three populations, for both males and females (Table 1). The main predictors of lytic activity, for both sexes, were the individual’s size and the lytic activity of their breeding partner. Larger individuals produced higher lytic activity, and individuals paired with beetles that had higher lytic activity also showed higher lytic activity themselves. Lytic activity did not differ significantly between males and females (ANOVA: *F*_1,722_ = 1.245, *P* = 0.265).

### Fitness Consequences for Broods from the Changes in the Nursery Environment.

#### Breeding success.

Across all populations, the presence of a hole on the surface of the carcass was the best predictor of breeding success (i.e., whether at least one larva would survive to dispersal; absence of hole: 79% successful [169 of 214]; presence of hole: 96% successful [211 of 219]) (Table 2). The number of successful broods did not differ across the three experimental populations, nor were broods more likely to be successful on FC-, NC-, or Nm-prepared carcasses after the effect of hole presence was controlled for statistically by including it in the model (Table 2).

**Table 2.** Summary of minimal adequate models explaining differences in the success, size, and mass of broods from FC, NC, and Nm populations on carcasses prepared by FC, NC, and Nm parents

Term	Estimate	SEM	DF	*z* value	*t* value	*P* value
Brood success: Binomial GLM						
Intercept	1.34	0.27		4.99		<0.001***
Population N	−0.38	0.36		−1.05		0.29
Population Nm	0.36	0.40		0.91		0.36
Hole	1.98	0.40		4.97		<0.001***
Brood size: GLMM						
Intercept	−8.64	7.10	354.55		−1.22	0.22
Population N	0.82	2.42	31.87		0.34	0.74
Population Nm	1.37	2.28	25.66		0.60	0.55
Carcass N	−6.68	2.11	359.17		−3.17	0.002**
Carcass Nm	−6.90	2.12	360.57		−3.25	0.001**
Female size	5.64	1.47	361.76		3.84	<0.001***
Hole	12.48	1.74	359.83		7.17	<0.001***
* *Interaction *Χ*^2^						
Population × carcass	15.03		4			0.005**
Population × hole	10.90		2			0.004**
Brood mass: GLMM						
Intercept	−0.75	0.73	355.39		−1.02	0.31
Population N	0.59	0.22	30.95		2.65	0.01*
Population Nm	0.55	0.21	25.16		2.63	0.01*
Carcass N	−0.41	0.22	356.45		−1.82	0.07
Carcass Nm	−0.43	0.23	358.85		−1.9	0.06
Female size	0.48	0.13	358.69		3.54	<0.001***
Hole present	0.8	0.21	356.62		3.83	<0.001***
Carcass roundness	0.4	0.4	356.71		1	0.32
* *Interaction *Χ^2^*						
Population × carcass	10.55		4			0.03*
Population × hole	15.13		2			<0.001
Carcass × hole	5.94		2			0.005

We present *z* values for binomial generalized linear model (GLM) and *T* values for generalized linear mixed models (GLMMs). Degrees of freedom (DF) for GLMMs calculated with Sattterthwaite's approximation.  Asterisks denote statistical significance. *P < 0.05; **P < 0.01; ***P < 0.001.

#### Clutch size and brood size.

**Fig. 2.**
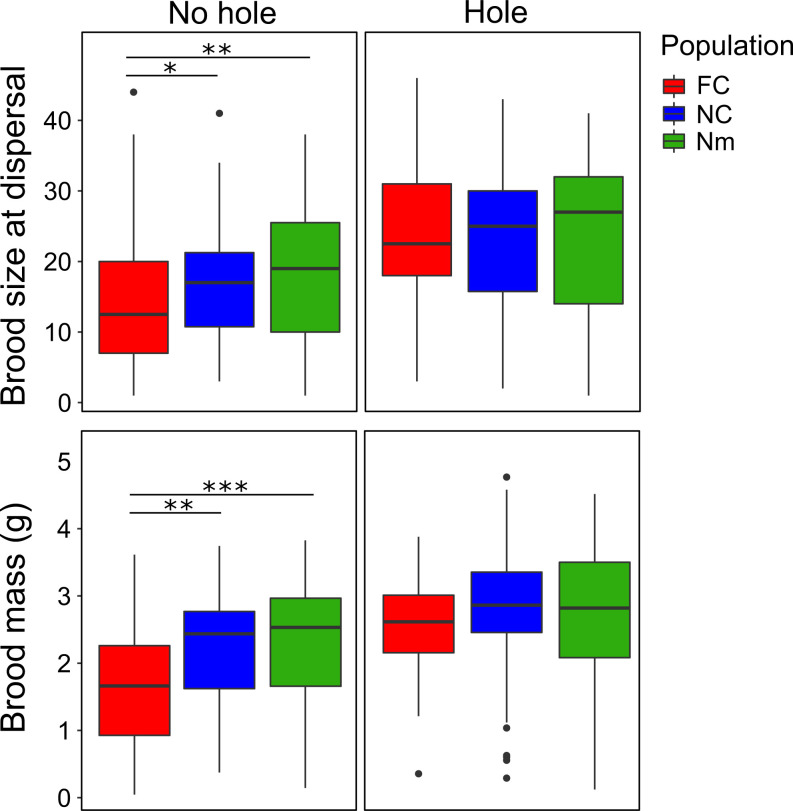
Brood size and brood mass of larvae at dispersal from FC, NC, and Nm broods (red, blue, and green box plots, respectively) developing on carcasses with and without a hole. Sample sizes: FC = 125, NC = 127, and Nm = 128. Box plots depict the first quartile, median, and third quartile. Whiskers on the box plots range from the sample’s lowest to highest value within 1.5× interquartile range. Points depict sample outliers. Significant differences between populations are indicated with asterisks (post hoc analyses with emmeans using the Tukey method; *P < 0.05; **P < 0.01; ***P < 0.001) (*SI Appendix*, Tables S2 and S4).

The number of eggs observed at the bottom of breeding boxes, a proxy measure for clutch size, was significantly larger in the FC populations than in the NC and Nm populations (on average, FC clutches had 4.21 and 4.15 more eggs than NC and Nm clutches, respectively) (*SI Appendix*, Fig. S2 and Table S1). Brood size varied across populations and also depended on which populations had prepared the carcasses that the larvae developed upon (Table 2). Despite FC females laying larger clutches, FC broods were not larger overall at dispersal. The presence of a hole was, of all the factors considered, the one with the largest positive effect on the number of larvae that survived to dispersal (Fig. 2 and Table 2) across all three populations. There was, however, a significant interaction between population and the presence/absence of a hole. In carcasses where no hole could be seen, FC broods were significantly smaller than both NC and Nm broods (Fig. 2 and *SI Appendix*, Table S2). There was no effect of carcass roundness on brood size (ANOVA between nested models with and without carcass roundness as a fixed effect: *Χ*^2^ = 2.16, *P* = 0.14).

**Fig. 3.**
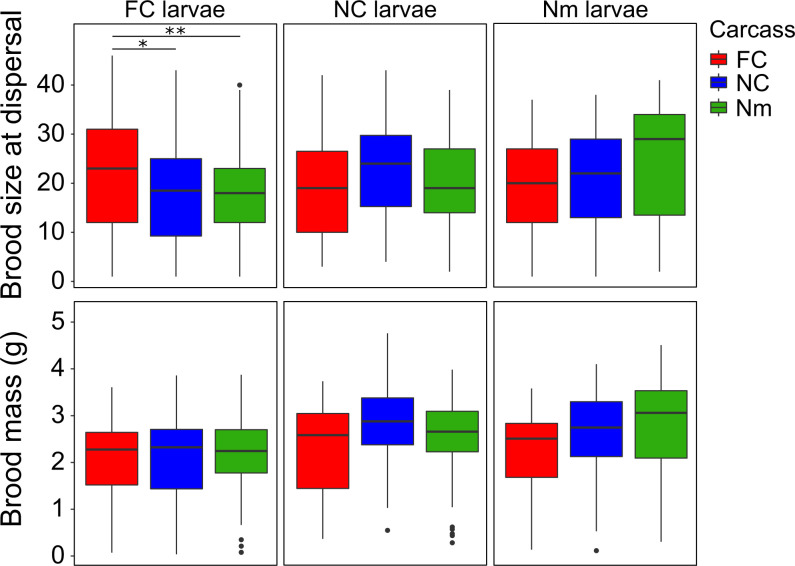
Brood size and brood mass of larvae at dispersal from FC, NC, and Nm broods developing on carcasses prepared by FC, NC, or Nm parents (red, blue, and green box plots, respectively). Sample sizes: FC = 125, NC = 127, and Nm = 128. Box plots depict the first quartile, median, and third quartile. Whiskers on the box plots range from the sample’s lowest to highest value within 1.5× interquartile range. Points depict sample outliers. Significant differences between populations are indicated with asterisks (post hoc analyses with emmeans using the Tukey method; *P < 0.05; **P < 0.01; ***P < 0.001) (*SI Appendix*, Tables S3 and S5).

A significant interaction was also found between the brood’s population of origin and the population of origin of the beetles that prepared the carcass (population × carcass) (Fig. 3 and Table 2). There was a tendency for broods to be larger at dispersal on carcasses prepared by parents of their own population of origin (Fig. 3 and *SI Appendix*, Table S3). FC larvae performed significantly better on carcasses prepared by FC parents than on carcasses prepared by NC and Nm parents (post hoc tests and 95% CIs) (*SI Appendix*, Table S3). NC and Nm larvae showed a nonsignificant tendency to perform better on carcasses prepared by NC and Nm parents, respectively (*SI Appendix*, Table S3).

#### Brood mass.

Brood mass varied across populations and carcasses prepared by different populations, in a similar way to brood size, with presence of a hole having the largest positive effect (Fig. 2 and Table 2). There was a significant interaction between the presence of a hole and the population of origin, with FC broods having significantly smaller mass than NC and Nm broods in the absence of a hole (post hoc tests and 95% CIs) (*SI Appendix*, Table S4). Again, there was a tendency for brood mass to be highest in broods reared on carcasses from their own parental population (Fig. 3) (post hoc tests and 95% CIs) (*SI Appendix*, Table S5). The minimal adequate model for brood mass also included a marginally significant interaction between the presence of a hole and the carcass of origin; post hoc tests suggest that the presence of a hole leads to broods attaining a greater mass at dispersal when reared on NC and Nm carcasses than on FC carcasses (post hoc tests and 95% CIs) (*SI Appendix*, Table S6). When running the minimal adequate model without random effects, the *P* values for population were lower than in the mixed model with Satterthwaite’s approximated degrees of freedom (LMM: *P* = 0.01 and *P* = 0.02 for NC and Nm, respectively; LM: *P* = 0.006 and *P* = 0.007 for NC and Nm, respectively).

#### Larval mortality between egg laying and larval dispersal.

To deduce the extent of larval mortality, we applied a best-fit regression on the entire dataset of brood size on clutch size (*F*_1,378_ = 148.5, *P* < 0.001, *R*^2^ = 0.28) (*SI Appendix*, Fig. S3 and Table S7) and used the residuals as a response variable in a GLMM (Table 3).

**Table 3.** Generalized linear mixed model of offspring mortality between egg laying and larval dispersal for FC, NC, and Nm populations reared on FC, NC, and Nm carcasses

Term	Estimate	SEM	DF	*t* value	*P* value
Offspring mortality between egg laying and dispersal: GLMM					
Intercept	−7.86	1.51	12.27	−5.19	<0.001***
Population N	4.8	2.17	12.82	2.22	0.045*
Population Nm	6.55	2.09	11.18	3.13	0.009**
Carcass N	−3.06	1.66	360.16	−1.85	0.07
Carcass Nm	−2.81	1.67	360.86	−1.68	0.09
Hole	11.81	1.37	360.38	8.62	<0.001***
Interaction *Χ^2^*					
Population × carcass	8.192		4		0.085****
Population × hole	11.752		2		0.003**

Residuals of a linear regression of brood size on egg count (proxy for clutch size) were used as the response variable (i.e., this was the measure of relative offspring mortality). Degrees of freedom (DF) calculated with Satterthwaite's approximation. Asterisks denote statistical significance. *P < 0.05; **P < 0.01; ***P < 0.001; ****P < 0.1.

**Fig. 4.**
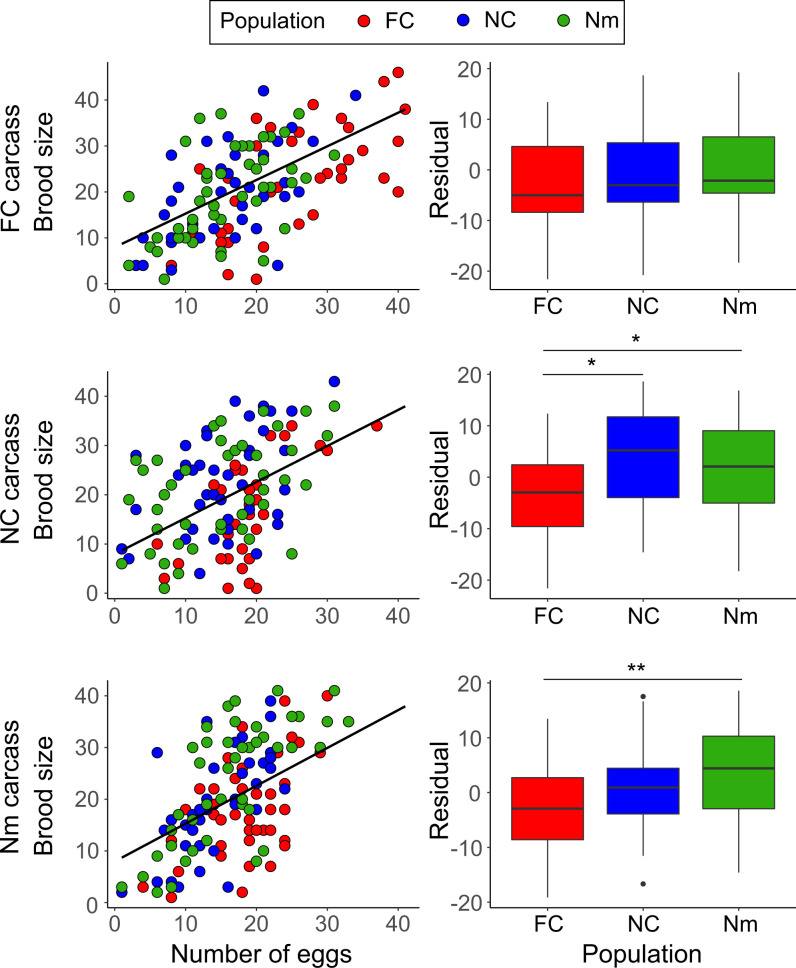
Number of larvae that survived from egg laying to larval dispersal in relation to clutch size (estimated from the number of eggs on the bottom of the breeding box). Figures show the FC, NC, and Nm populations (red, blue, and green circles and box plots, respectively) developing on FC- (*Top*), NC- (*Middle*), or Nm-prepared carcasses (*Bottom*). Each data point in *Right* represents a different brood. Sample sizes: FC = 125, NC = 127, and Nm = 128. A regression model for brood size was fit to all the data (black lines in *Left*; *R*^2^ = 0.28) (*SI Appendix*, Fig. S2 and Table S7), and the residuals from those lines (*Right*) were used as response variables in linear mixed models (i.e., this was our measure of relative offspring mortality). Box plots depict the first quartile, median, and third quartile. Whiskers on the box plots range from the sample’s lowest to highest value within 1.5× interquartile range. Points depict sample outliers. Significant differences between populations (indicated with an asterisk; *P < 0.05; **P < 0.01; ***P < 0.001) were found in NC- and Nm-prepared carcasses but not FC-prepared carcasses. Post hoc analyses were performed with emmeans using the Tukey method (Tables 4 and 5).

Overall, there was a population effect on larval mortality, with FC populations showing higher mortality than NC or Nm populations, thus potentially explaining why FC broods were not on average larger, despite having larger clutch sizes. Again, we found a significant interaction between the larval population of origin and the presence of a hole in the carcass. On carcasses without a hole, FC populations showed greater offspring mortality between egg laying and larval dispersal than NC and Nm populations (Table 3 and *SI Appendix*, Table S8). An interaction between the larval population of origin and the carcass population of origin (population × carcass) (Table 3) was retained in the minimal adequate model, despite being only marginally significant, because removing it did not decrease AIC. Post hoc tests revealed that FC offspring were most likely to die between egg laying and larval dispersal across all types of carcasses (Tables 4 and 5). There was again a tendency for larvae to perform better on carcasses prepared by parents of their own parental population (Fig. 4 and Tables 4 and 5). Offspring were equally likely to survive on FC-prepared carcasses, regardless of their population of origin. However, FC populations were significantly more likely to die between egg laying and larval dispersal than NC and Nm populations when they developed on NC-prepared carcasses. On Nm-prepared carcasses, FC populations differed significantly only from Nm populations in the likelihood that larvae would die between egg laying and larval dispersal (Tables 4 and 5). When running the minimal adequate model without random effects, the *P* values for population were lower than in the mixed model with approximated degrees of freedom (LMM: *P* = 0.045 and *P* = 0.009 for NC and Nm, respectively; LM: *P* = 0.009 and *P* = 0.0002 for NC and Nm, respectively).

**Table 4.** Variation in offspring mortality between egg laying and larval dispersal due to the interaction between population and carcass type: least-square means (LS) and 95% confidence levels (CL)

Carcass	Population	LS means	SE	DF	Lower CL	Upper CL
FC	FC	−1.95	1.44	10.09	−5.16	1.25
FC	NC	0.50	1.47	10.86	−2.73	3.74
FC	Nm	1.27	1.42	9.44	−1.91	4.45
NC	FC	−5.01	1.42	9.63	−8.20	−1.82
NC	NC	2.29	1.48	11.19	−0.96	5.54
NC	Nm	1.29	1.43	9.72	−1.90	4.48
Nm	FC	−4.76	1.43	9.83	−7.96	−1.56
Nm	NC	−0.17	1.49	11.55	−3.43	3.10
Nm	Nm	3.24	1.41	9.14	0.07	6.41

Residuals of a linear regression of brood size on egg count (proxy for clutch size) were used as the response variable (i.e., this was the measure of relative offspring mortality). The package emmeans was used to calculate LS means and perform the post hoc pairwise comparisons using *P* value adjustment with the Tukey method for multiple comparisons. Degrees of freedom (DF) calculated with Satterthwaite's approximation.

**Table 5.** Variation in offspring mortality between egg laying and larval dispersal due to the interaction between population and carcass type: estimated differences between populations within carcass types

Carcass	Contrast	Estimate	SE	DF	*t* ratio	*P* value
FC	FC – NC	−2.458	2.057	10.470	−1.195	0.481
FC	FC – Nm	−3.222	2.021	9.761	−1.594	0.293
FC	NC – Nm	−0.764	2.040	10.138	−0.375	0.926
NC	FC – NC	−7.301	2.053	10.394	−3.557	0.012*
NC	FC – Nm	−6.302	2.016	9.674	−3.126	0.028*
NC	NC – Nm	0.999	2.055	10.442	0.486	0.879
Nm	FC – NC	−4.594	2.066	10.671	−2.223	0.112
Nm	FC – Nm	−8.000	2.006	9.483	−3.989	0.007**
Nm	NC – Nm	−3.406	2.048	10.313	−1.663	0.265

Residuals of a linear regression of brood size on egg count (proxy for clutch size) were used as the response variable (i.e., this was the measure of relative offspring mortality). The package emmeans was used to calculate least-square means and perform the post hoc pairwise comparisons using *P* value adjustment with the Tukey method for multiple comparisons. Degrees of freedom (DF) was calculated with Satterthwaite's approximation. Asterisks denote statistical significance. *P < 0.05; **P < 0.01.

## Discussion

We found that when experimental populations were prevented from supplying care directly to their young for several generations, individuals adapted by modifying the way they constructed the nursery in which their offspring developed. Even though they could no longer meet their larvae, parents were still able to enhance their offspring’s fitness through this modified form of indirect care. Importantly, Nm parents, which were reared with posthatching care, showed the same response as NC parents, which had not experienced parental care themselves. Hence, the differences we observed between FC and NC parents were not a result of phenotypic plasticity in response to their own early life environment but represented evolved divergence in this trait.

We measured change in several facets of nursery construction, and the results offer preliminary insights into the modular nature of carcass preparation and the extent to which the different elements are subject to different selection pressures and are genetically uncorrelated. The prehatching care trait with the strongest fitness consequences to the brood was the timing of insertion of a hole in the carcass. When we tested the adaptive value of the new nursery environment, in a previous study, by inserting a hole in the carcass ourselves and forcing larvae to develop with no posthatching care, we observed increased brood survival, brood size, and mass and more surviving larvae for a given clutch size—regardless of the population from which larvae were drawn (22). By biting this hole in the carcass before they were removed, parents probably enabled larvae to enter the carcass, to feed upon it, and to take up residence there (19, 22), even in the parents’ absence. Furthermore, the fitness gained by the evolution of this extended parental care phenotype helped to compensate for the fitness lost from the experimental removal of direct care; by generation 13, parents from the NC and FC populations produced similar numbers of larvae per gram of carrion, as shown in ref. 27.

If the presence of a hole has such strong fitness consequences when parents are absent, why did roughly 70% of FC and 40% of NC and Nm parents still not bite a hole before larval hatching? The results for FC parents are unsurprising because they typically remain with their brood under their selective regime. Unlike NC parents, FC parents had the opportunity to make entrance holes in the carcass after their larvae hatched, as they crawled through the soil to the carcass. The proportion of FC parents making a hole in the carcass before larval hatching is close to the proportion found in wild-caught beetles breeding in the laboratory (∼26%) (22). As for the NC parents, it is possible that the accompanying evolution of morphological and behavioral larval traits (22, 28) helped to decrease selective pressure on accelerated hole biting by parents, or it may be that this trait was still under selection and spread further through the NC population in subsequent generations. Either possibility can explain why we did not observe all NC parents biting a hole prior to their removal by the 13th generation of experimental evolution.

Focusing on carcass roundness, we found that carcasses prepared by parents from the NC populations were rounder by the time we removed parents. In FC populations, by contrast, only larger parents were able to produce rounder carrion nests. However, just as in a previous study (25), we found no evidence that carcass roundness affected brood performance directly. Why, then, did we observe a change in this trait?

One possibility is that the shape of the carcass itself is not the trait under selection but that it changes as a by-product of selection on the pace of carcass preparation. Our experimental protocol placed NC beetles under selection to complete carcass preparation within 53 h to ensure they had bitten a hole for their larvae before they were removed and perhaps thereby incidentally favored beetles that had prepared rounder nests. FC parents, by contrast, are not under selection to complete this task as quickly. Furthermore, converting the dead body into a carrion nest is likely to be energetically costly because it involves rolling the corpse around and pushing it against the soil. This might explain why smaller beetles, with fewer energy reserves, engaged in carcass preparation less vigorously and, consequently, produced less rounded carcasses in the same timeframe in the FC lines (and also as observed in ref. 25). The strong fitness consequences of the presence of a hole contrast with the seeming lack of fitness consequences of carcass roundness, and suggest that both traits belong to the same behavioral module. Strong selection on the timing of the hole could, therefore, have dragged the rest of the behavioral module with it as a correlated response, resulting in rounder carcasses. It would be interesting to test whether this speculation is correct or whether carcass rolling is in fact a distinct behavioral module.

We found no difference between our experimental populations in a third trait associated with carcass preparation—the lytic activity of the anal exudates—which is again consistent with the suggestion that carcass preparation comprises distinct behavioral modules. Our results imply that the different modules evolve under different selection pressures and are not strongly genetically correlated, much as has been found in the extended parental phenotypes of bees (44) and mice (45). This could explain why some elements of carcass preparation have evolved in response to the elimination of posthatching care, while others have not.

An additional explanation for the lack of differences in lytic activity between experimental populations, which does not exclude the suggestion of modularity, is that the NC larvae have evolved stronger lytic activity in their antimicrobial exudates (46–48). A further explanation is that the lytic activity of the anal exudates is a highly plastic trait (24), and its plasticity prevented any evolutionary change. All these interpretations remain to be tested in future work.

We detected evolved change in the way that parents constructed the nursery environment after only 13 generations of experimental evolution. The most likely explanation is that we selected on existing standing genetic variation in the mix of wild populations that founded the experimental populations. By mixing different wild populations, we intentionally increased the genetic variation in our experimental laboratory populations. We must, therefore, be cautious not to directly extrapolate our results to wild populations and risk overestimating their ability to respond to similar selective pressures. Nevertheless, the question remains of how so much genetic variation is able to persist naturally over relatively small geographic distances. One possibility is that there is genetic differentiation between the different wild populations that we sampled originally (49). We have recently found that even nearby populations can be divergently adapted to the specific local conditions within their woodland (50). It is also possible that there is considerable genetic variation in parental care within populations, which is maintained by variation in key environmental conditions governing each breeding attempt, such as the species of the dead animal, the density of carrion, and the extent of competition within and among species for the opportunity to breed upon it (51).

The extended parental care phenotype was not the only trait to diversify across populations. NC populations had smaller clutch sizes than FC populations. Typically, burying beetles lay more eggs than can successfully be reared on a carcass and then, cull the hatchlings to adjust brood size to the existing resources (52). It is possible that NC populations are selected to lay fewer eggs that more accurately match the existing resources because they are removed before they can cull any hatchlings. This hypothesis is currently under investigation in our laboratory.

Furthermore, the cross-fostering experiment suggests that larvae were divergently and locally adapted to develop under NC vs. FC. FC larvae not only suffered higher mortality in the absence of posthatching care than NC larvae, they were also more likely than NC larvae to die when developing in carcasses prepared by parents from other lineages. NC larvae also tended to perform better in carcasses prepared by their own lineage, although this was not statistically significant—perhaps because our experiment did not have enough statistical power to detect very small effects, despite a large sample size. Why is the effect size larger for FC larvae than for NC larvae? One possibility is that NC larvae mostly rely on their own traits to secure access to the carcass, whereas FC larvae are more locally adapted to a particular extended parental phenotype. We know from previous work that NC larvae have evolved relatively larger mandibles (22), a propensity to hatch more synchronously (53), and an inclination to behave more cooperatively toward siblings (28). However, further work is needed to fully understand the extent and mechanisms of local adaptation to the extended parental phenotype in FC vs. NC larvae.

How does carcass preparation in burying beetles compare with other forms of extended parental phenotypes? Despite the widespread occurrence of extended parental phenotypes across taxa, the fitness consequences are likely to depend on the extent of parental presence during offspring development. In species with no direct parental care, such as the mass provisioning beewolves and dung beetles, it is likely that the extended parental phenotype has strong fitness consequences. In dung beetles, the size of the brood mass (the ball of dung with developing larvae) that the mother stockpiles determines many offspring traits (4); in beewolves, the antimicrobial secretions applied by the parent protect the larvae against pathogens during development (5). In other species, direct parental care may mask the adaptive value of extended parental phenotypes. For example, egg size can be considered an extended parental phenotype, yet evidence that avian egg size correlates with offspring survival is inconclusive (reviewed in ref. 54). As pointed out in ref. 54, large eggs can benefit offspring survival in harsh environments, but in good conditions, parental provisioning masks any effect of egg size on survival. Interestingly, the same occurs in burying beetles, where effects of egg size on survival are masked by posthatching care (55). Our findings regarding the timing of carcass preparation parallel results obtained previously for egg size in burying beetles and birds. It suggests that the stability of the environment (whether social or abiotic) affects the evolutionary feedbacks between different forms of parental care. In future work, it will be important to test whether these evolutionary feedbacks exist in natural populations, where the environment is more complex and unpredictable than in the laboratory.

Parental care comprises a suite of traits that are integrated to promote offspring fitness (56). Previous work has emphasized that the wider physical environment and the social environment within the family are each sources of selection on the form and function of parental behavior (56–59). Here, we have shown that acts of care are themselves a source of selection on other types of parental traits. Preventing any form of direct care causes parents to evolve modifications in the way in which they construct the nursery environment. Furthermore, coadapted traits in parents and offspring evolved rapidly when we experimentally eliminated direct forms of posthatching care, suggesting that these traits have a high degree of genetic variability in natural populations.

## Supplementary Material

Supplementary File

## Data Availability

All data, code for image analysis and statistical analysis are available in the *SI Appendix*.

## References

[1] N. J. Royle, P. T. Smiseth, M. Kölliker, Eds., The Evolution of Parental Care (Oxford University Press, 2012).

[2] T. H. Clutton-Brock, The Evolution of Parental Care (Princeton University Press, 1991).

[3] R. Dawkins, The Extended Phenotype—The Long Reach of the Gene (Oxford University Press, ed. 2, 1999).

[4] J. Hunt, L. W. Simmons, Patterns of fluctuating asymmetry in beetle horns: An experimental examination of the honest signalling hypothesis. Behav. Ecol. Sociobiol. 41, 109–114 (1997).

[5] S. Koehler, J. Doubský, M. Kaltenpoth, Dynamics of symbiont-mediated antibiotic production reveal efficient long-term protection for beewolf offspring. Front. Zool. 10, 3 (2013). 23369509 10.1186/1742-9994-10-3 PMC3599432

[6] K. L. Chiquillo, D. A. Ebert, C. J. Slager, K. D. Crow, The secret of the mermaid’s purse: Phylogenetic affinities within the Rajidae and the evolution of a novel reproductive strategy in skates. Mol. Phylogenet. Evol. 75, 245–251 (2014). 24486989 10.1016/j.ympev.2014.01.012 PMC4036632

[7] D. A. Warner, R. Shine, The adaptive significance of temperature-dependent sex determination: Experimental tests with a short-lived lizard. Evolution 59, 2209–2221 (2005). 16405164

[8] C. P. Andrews, L. E. B. Kruuk, P. T. Smiseth, Evolution of elaborate parental care: Phenotypic and genetic correlations between parent and offspring traits. Behav. Ecol. 28, 39–48 (2017). 28127224 10.1093/beheco/arw129 PMC5255903

[9] J. E. Mank, D. E. L. Promislow, J. C. Avise, Phylogenetic perspectives in the evolution of parental care in ray-finned fishes. Evolution 59, 1570–1578 (2005). 16153042

[10] D. P. Wetzel, D. F. Westneat, Parental care syndromes in house sparrows: Positive covariance between provisioning and defense linked to parent identity. Ethology 120, 249–257 (2014).

[11] N. DiRienzo, H. Aonuma, Plasticity in extended phenotype increases offspring defence despite individual variation in web structure and behaviour. Anim. Behav. 138, 9–17 (2018). 30364586 10.1016/j.anbehav.2018.01.022 PMC6197064

[12] N. Vasey, M. Mogilewsky, G. E. Schatz, Infant nest and stash sites of variegated lemurs (*Varecia rubra*): The extended phenotype. Am. J. Primatol. 80, e22911 (2018). 30187943 10.1002/ajp.22911

[13] E. Pukowski, Ecological investigation of *Necrophorus* F. Zoomorphology 27, 518–586 (1933).

[14] M. P. Scott, The ecology and behavior of burying beetles. Annu. Rev. Entomol. 43, 595–618 (1998). 15012399 10.1146/annurev.ento.43.1.595

[15] S. C. Cotter, R. M. Kilner, Sexual division of antibacterial resource defence in breeding burying beetles, *Nicrophorus vespilloides.* J. Anim. Ecol. 79, 35–43 (2010). 19627394 10.1111/j.1365-2656.2009.01593.x

[16] A. Duarte, M. Welch, C. Swannack, J. Wagner, R. M. Kilner, Strategies for managing rival bacterial communities: Lessons from burying beetles. J. Anim. Ecol. (2017). 10.1111/1365-2656.12725 PMC5836980 28682460

[17] P. T. Smiseth, C. T. Darwell, A. J. Moore, Partial begging: An empirical model for the early evolution of offspring signalling. Proc. Biol. Sci. 270, 1773–1777 (2003). 12964978 10.1098/rspb.2003.2444 PMC1691438

[18] M. Schrader, B. J. M. Jarrett, R. M. Kilner, Using experimental evolution to study adaptations for life within the family. Am. Nat. 185, 610–619 (2015). 25905504 10.1086/680500 PMC4497813

[19] A. Eggert, M. Reinking, J. K. Müller, Parental care improves offspring survival and growth in burying beetles. Anim. Behav. 55, 97–107 (1998). 9480676 10.1006/anbe.1997.0588

[20] O. De Gasperin, A. Duarte, R. M. Kilner, Interspecific interactions explain variation in the duration of paternal care in the burying beetle. Anim. Behav. 109, 199–207 (2015). 26778845 10.1016/j.anbehav.2015.08.014 PMC4686539

[21] D. J. Parker , Transcriptomes of parents identify parenting strategies and sexual conflict in a subsocial beetle. Nat. Commun. 6, 8449 (2015). 26416581 10.1038/ncomms9449 PMC4598741

[22] B. J. M. Jarrett , A sustained change in the supply of parental care causes adaptive evolution of offspring morphology. Nat. Commun. 9, 3987 (2018). 30266903 10.1038/s41467-018-06513-6 PMC6162320

[23] A. N. Arce, P. R. Johnston, P. T. Smiseth, D. E. Rozen, Mechanisms and fitness effects of antibacterial defences in a carrion beetle. J. Evol. Biol. 25, 930–937 (2012). 22409257 10.1111/j.1420-9101.2012.02486.x

[24] S. C. Cotter, E. Topham, A. J. P. Price, R. M. Kilner, Fitness costs associated with mounting a social immune response. Ecol. Lett. 13, 1114–1123 (2010). 20545735 10.1111/j.1461-0248.2010.01500.x

[25] O. De Gasperin, A. Duarte, J. Troscianko, R. M. Kilner, Fitness costs associated with building and maintaining the burying beetle’s carrion nest. Sci. Rep. 6, 35293 (2016). 27734965 10.1038/srep35293 PMC5062497

[26] R. M. Kilner , Parental effects alter the adaptive value of an adult behavioural trait. eLife 4, e07340 (2015). 26393686 10.7554/eLife.07340 PMC4613925

[27] M. Schrader, B. J. M. Jarrett, D. Rebar, R. M. Kilner, Adaptation to a novel family environment involves both apparent and cryptic phenotypic changes. Proc. Biol. Sci. 284, 20171295 (2017). 28878064 10.1098/rspb.2017.1295 PMC5597835

[28] D. Rebar, N. W. Bailey, B. J. M. Jarrett, R. M. Kilner, An evolutionary switch from sibling rivalry to sibling cooperation, caused by a sustained loss of parental care. Proc. Natl. Acad. Sci. 117, 2544–2550 (2020). 31964847 10.1073/pnas.1911677117 PMC7007579

[29] M. Schrader, B. J. M. Jarrett, R. M. Kilner, Parental care masks a density-dependent shift from cooperation to competition among burying beetle larvae. Evolution 69, 1077–1084 (2015). 25648525 10.1111/evo.12615 PMC4476075

[30] N. Pilakouta, S. Jamieson, J. A. Moorad, P. T. Smiseth, Parental care buffers against inbreeding depression in burying beetles. Proc. Natl. Acad. Sci. U.S.A. 112, 8031–8035 (2015). 26080412 10.1073/pnas.1500658112 PMC4491787

[31] G. Boncoraglio, R. M. Kilner, Female burying beetles benefit from male desertion: Sexual conflict and counter-adaptation over parental investment. PLoS One 7, e31713 (2012). 22355390 10.1371/journal.pone.0031713 PMC3280230

[32] P. T. Smiseth, S. Musa, A. J. Moore, Negotiation between parents: Does the timing of mate loss affect female compensation in *Nicrophorus vespilloides*? Behaviour 143, 293–301 (2006).

[33] M. Schrader, R. M. Crosby, A. R. Hesketh, B. J. M. Jarrett, R. M. Kilner, A limit on the extent to which increased egg size can compensate for a poor postnatal environment revealed experimentally in the burying beetle, *Nicrophorus vespilloides.* Ecol. Evol. 6, 329–336 (2015). 26811796 10.1002/ece3.1876 PMC4716521

[34] S.-J. Sun, R. M. Kilner, Temperature stress induces mites to help their carrion beetle hosts by eliminating rival blowflies. eLife 9, e55649 (2020). 32755542 10.7554/eLife.55649 PMC7431131

[35] S. C. Cotter, R. M. Kilner, Personal immunity versus social immunity. Behav. Ecol. 21, 663–668 (2010).

[36] W. J. Palmer , A gene associated with social immunity in the burying beetle *Nicrophorus vespilloides*. Proc. Biol. Sci. 283, 20152733 (2016). 26817769 10.1098/rspb.2015.2733 PMC4795035

[37] H. Vogel , The digestive and defensive basis of carcass utilization by the burying beetle and its microbiota. Nat. Commun. 8, 15186 (2017). 28485370 10.1038/ncomms15186 PMC5436106

[38] C. E. Reavey, Investigating the Immune and Reproductive Strategies of Burying Beetles, Nicrophorus vespilloides (Queen’s University Belfast, 2015).

[39] R Core Team, *R: A Language and Environment for Statistical Computing,* version 4.1.1. *R Foundation for Statistical Computing*, Vienna, Austria (2021).

[40] D. Bates, M. Mächler, B. Bolker, S. Walker, Fitting linear mixed-effects models using lme4. J. Stat. Softw. 67, 1–48 (2015).

[41] R. Lenth, emmeans: Estimated marginal means, aka least-squares means. R package version 1.6.3.* R Foundation for Statistical Computing*, Vienna, Austria (2019).

[42] A. F. Zuur, E. N. Ieno, N. J. Walker, A. A. Saveliev, G. M. Smith, “The linear mixed effects model” in Mixed Effects Models and Extensions in Ecology with R, M. Gail, K. Krickeberg, J. M. Samet, A. Tsiatis, W. Wong, Eds. (*Statistics for Biology and Health*, Springer, New York, NY, 2009), pp. 105–111.

[43] A. Kuznetsova, P. Bruun Brockhoff, R. Haubo Bojesen Christensen, lmerTest Package: Tests in Linear Mixed Effects Models. *J*. *Stat*. *Softw*. , 1–26 (2017).

[44] R. Royauté , Phenotypic integration in an extended phenotype: Among-individual variation in nest-building traits of the alfalfa leafcutting bee (*Megachile rotundata*). J. Evol. Biol. 31, 944–956 (2018). 29499106 10.1111/jeb.13259

[45] J. N. Weber, B. K. Peterson, H. E. Hoekstra, Discrete genetic modules are responsible for complex burrow evolution in *Peromyscus* mice. Nature 493, 402–405 (2013). 23325221 10.1038/nature11816

[46] C. E. Reavey, L. Beare, S. C. Cotter, Parental care influences social immunity in burying beetle larvae. Ecol. Entomol. 39, 395–398 (2014).

[47] A. N. Arce, P. T. Smiseth, D. E. Rozen, Antimicrobial secretions and social immunity in larval burying beetles, *Nicrophorus vespilloides.* Anim. Behav. 86, 741–745 (2013).

[48] A. Duarte , Social immunity of the family: Parental contributions to a public good modulated by brood size. Evol. Ecol. 30, 123–135 (2016). 26900202 10.1007/s10682-015-9806-3 PMC4750363

[49] S. Pascoal, R. M. Kilner, Development and application of 14 microsatellite markers in the burying beetle *Nicrophorus vespilloides* reveals population genetic differentiation at local spatial scales. PeerJ 5, e3278 (2017). 28480146 10.7717/peerj.3278 PMC5417058

[50] S.-J. Sun , Rapid local adaptation linked with phenotypic plasticity. Evol. Lett. 4, 345–359 (2020). 32774883 10.1002/evl3.176 PMC7403679

[51] N. J. Royle, P. E. Hopwood, Covetable corpses and plastic beetles—The socioecological behavior of burying beetles. Adv. Stud. Behav. 49, 101–146 (2017).

[52] J. Bartlett, C. M. Ashworth, Brood size and fitness in *Nicrophorus vespilloides* (Coleoptera, Silphidae). Behav. Ecol. Sociobiol. 22, 429–434 (1988).

[53] B. J. M. Jarrett , Adaptive evolution of synchronous egg-hatching in compensation for the loss of parental care. Proc. Biol. Sci. 285, 20181452 (2018). 30158310 10.1098/rspb.2018.1452 PMC6125895

[54] J. K. Christians, Avian egg size: Variation within species and inflexibility within individuals. Biol. Rev. Camb. Philos. Soc. 77, 1–26 (2002). 11911371 10.1017/s1464793101005784

[55] K. M. Monteith, C. Andrews, P. T. Smiseth, Post-hatching parental care masks the effects of egg size on offspring fitness: A removal experiment on burying beetles. J. Evol. Biol. 25, 1815–1822 (2012). 22775779 10.1111/j.1420-9101.2012.02567.x

[56] C. A. Walling, C. E. Stamper, P. T. Smiseth, A. J. Moore, The quantitative genetics of sex differences in parenting. Proc. Natl. Acad. Sci. U.S.A. 105, 18430–18435 (2008). 19008350 10.1073/pnas.0803146105 PMC2587554

[57] R. D. Montgomerie, P. J. Weatherhead, Risks and rewards of nest defence by parent birds. Q. Rev. Biol. 63, 167–187 (1988).

[58] A. F. Russell, N. E. Langmore, J. L. Gardner, R. M. Kilner, Maternal investment tactics in superb fairy-wrens. Proc. Biol. Sci. 275, 29–36 (2008). 17956851 10.1098/rspb.2007.0821 PMC2562397

[59] S. M. Caro, A. S. Griffin, C. A. Hinde, S. A. West, Unpredictable environments lead to the evolution of parental neglect in birds. Nat. Commun. 7, 10985 (2016). 27023250 10.1038/ncomms10985 PMC4820566

