# Supplementary material for: Evolutionary change in the construction of the nursery environment when parents are prevented from caring for their young directly
Source: Proc Natl Acad Sci U S A. 2021 Nov 24;118(48):e2102450118. doi: 10.1073/pnas.2102450118 (PMC8640939; doi:10.1073/pnas.2102450118)
Supplement: Supplementary File [file pnas.2102450118.sapp.pdf]

PNAS
www.pnas.org

Supplementary Information for
Evolutionary change in the construction of the nursery environment when parents are prevented from caring for their young directly.

Ana Duarte, Darren Rebar, Allysa C Hallett, Benjamin J M Jarrett, Rebecca M Kilner

Ana Duarte
Email: a.duarte@exeter.ac.uk

This PDF file includes:

- Figures S1 to S3
- Tables S1 to S8
- R code for statistical models and figures
- ImageJ script for analysis of carcass photos

Other supplementary materials for this manuscript include the following:

- Datasets S1

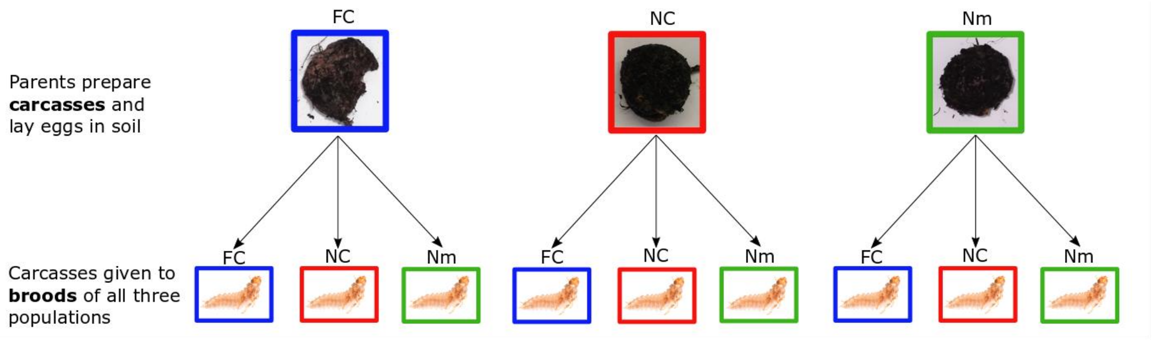

Fig. S1. Fully factorial experimental design: FC, NC and Nm parents were allowed to prepare carcasses and lay eggs. Carcasses were then given to other broods from all three populations, such that FC, NC or Nm broods developed in either FC, NC or Nm carcasses. Importantly, all broods developed on carcasses prepared by adults unrelated to them.

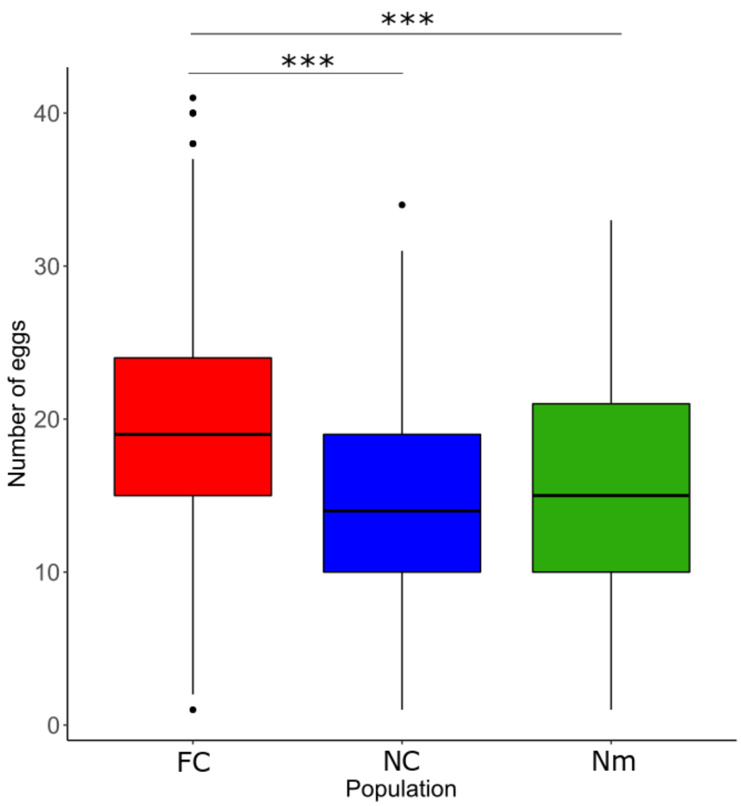

Fig. S2. Number of eggs counted at the bottom of breeding boxes 53h after pairing for each experimental population. Box plots depict first quartile, median and third quartile. Whiskers on the box plots range from the sample's lowest to highest value within 1.5 × inter-quartile range (IQR). Points depict sample outliers. Significant differences between populations are indicated with asterisks (*$P < 0.05$; **$P < 0.01$; ***$P < 0.001$).

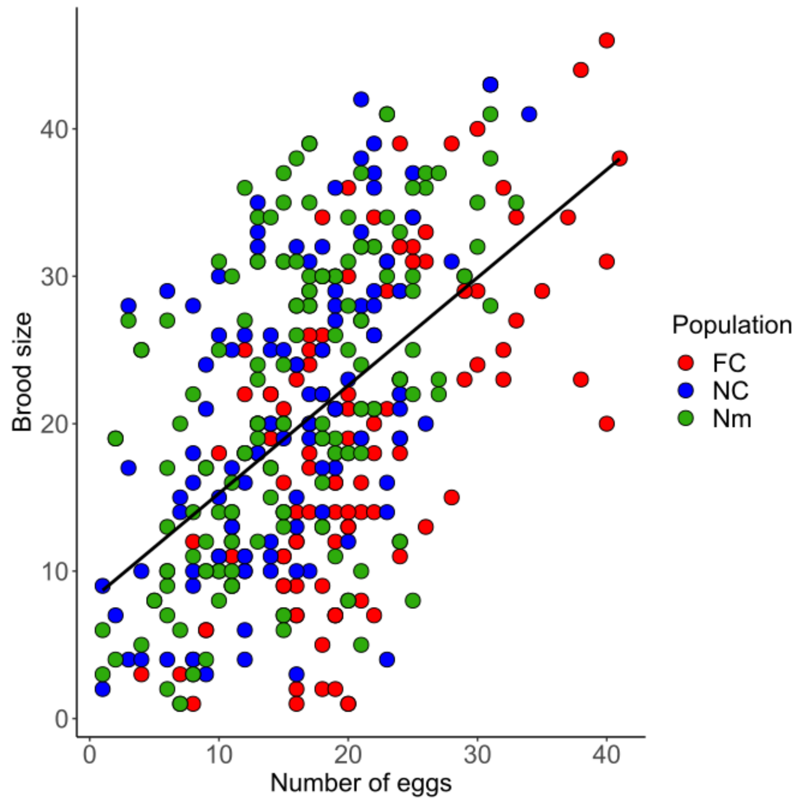

Fig. S3. Relationship between brood size on the number of eggs counted at the bottom of the breeding boxes. Each datapoint represents a different brood. Black line shows the “best fit” regression line for the data. Failed broods were not included in the regression.

Table S1. Variation in clutch size (number of eggs at the bottom of breeding box): summary of linear mixed model, with a population by block random effect. Asterisks denote statistical significance (**P* < 0.05; ***P* < 0.01; ****P* < 0.001).

**Term**	**Estimate**	**SE**	***t*-value**	***p***
Intercept	19.15	0.60	32.07	<0.001 ***
Population NC	-4.21	0.90	-4.72	<0.001 ***
Population Nm	-4.15	0.83	-5.00	<0.001 ***
Female size	2.62	0.35	7.50	<0.001 ***
Male size	-0.58	0.35	-1.64	0.10

Table S2. Brood size at dispersal: interaction between population of origin and presence of a hole in the carcass. The table shows least-square means and pairwise differences between populations in the absence and presence of a hole on the carcass. The package ‘emmeans’ was used to calculate least-square (LS) means and perform the pairwise comparisons, using P-value adjustment with Tukey method for multiple comparisons. DF denotes degrees of freedom, calculated with Satterthwaite’s approximation. Confidence level (CL) used was 0.95. Asterisks denote statistical significance (*P < 0.05; **P < 0.01; ***P < 0.001).

**Hole presence**	**Population**	**LS means**	**SE**	**DF**	**Lower CL**	**Upper CL**
No hole	FC	12.303	1.339	12.572	9.400	15.207
	NC	17.539	1.488	18.680	14.421	20.657
	Nm	19.069	1.295	10.998	16.217	21.920
Hole	FC	24.781	1.244	9.387	21.985	27.578
	NC	24.018	1.248	9.445	21.214	26.822
	Nm	23.798	1.234	9.085	21.010	26.585
**Hole presence**	**Contrast**	**Estimate**	**SE**	**DF**	***t*-ratio**	***p***
No hole	FC – NC	-5.236	2.013	15.800	-2.601	0.048 *
	FC - Nm	-6.766	1.864	11.789	-3.630	0.009 **
	NC - Nm	-1.530	1.973	14.613	-0.776	0.723
Hole	FC – NC	0.763	1.781	9.833	0.429	0.905
	FC - Nm	0.984	1.751	9.220	0.562	0.843
	NC - Nm	0.220	1.757	9.294	0.125	0.991

Table S3. Brood size at dispersal: interaction between the brood's population of origin and carcass upon which broods were raised. The table shows least-square means and pairwise differences between broods raised in FC, NC, and Nm carcasses, originating from FC, NC and Nm populations. The package 'emmeans' was used to calculate least-square (LS) means and perform the pairwise comparisons, using P-value adjustment with Tukey method for multiple comparisons. DF denotes degrees of freedom, calculated with Satterthwaite's approximation. Confidence level (CL) used was 0.95. Asterisks denote statistical significance (*$P < 0.05$; **$P < 0.01$; ***$P < 0.001$).

Population	Carcass	LS means	SE	DF	Lower CL	Upper CL
FC	FC	23.07	1.56	22.04	19.04	27.09
	NC	16.39	1.52	20.11	12.43	20.35
	Nm	16.17	1.55	21.20	12.17	20.17
NC	FC	20.89	1.61	24.82	16.78	25.00
	NC	22.22	1.60	24.68	18.11	26.32
	Nm	19.23	1.62	25.87	15.08	23.37
Nm	FC	20.56	1.51	19.38	16.62	24.50
	NC	20.47	1.52	20.24	16.50	24.43
	Nm	23.27	1.49	18.60	19.37	27.18
**Population**	**Contrast**	**Estimate**	**SE**	**DF**	***t*-ratio**	***p***
FC	FC – NC	6.68	2.10	359.09	3.17	0.005 **
	FC – Nm	6.90	2.12	360.53	3.25	0.004 **
	NC – Nm	0.22	2.06	360.05	0.11	0.99
NC	FC – NC	-1.33	2.24	359.07	-0.59	0.82
	FC – Nm	1.66	2.23	359.12	0.75	0.74
	NC – Nm	2.99	2.12	359.34	1.41	0.34
Nm	FC – NC	0.09	2.12	359.70	0.04	1.00
	FC – Nm	-2.71	2.07	359.33	-1.31	0.39
	NC – Nm	-2.80	2.02	359.10	-1.39	0.35

Table S4. Brood mass at dispersal: interaction between population of origin and presence of a hole in the carcass. The table shows least-square means and pairwise differences in brood mass, when broods originating from FC, NC or Nm populations were raised in carcasses with or without a hole. The package ‘emmeans’ was used to calculate least-square (LS) means and perform the pairwise comparisons, using P-value adjustment with Tukey method for multiple comparisons. DF denotes degrees of freedom, calculated with Satterthwaite’s approximation. Confidence level (CL) used was 0.95. Asterisks denote statistical significance (*P < 0.05; **P < 0.01; ***P < 0.001).

**Hole presence**	**Population**	**LS means**	**SE**	**DF**	**Lower CL**	**Upper CL**
No hole	FC	1.45	0.12	12.55	1.18	1.72
	NC	2.22	0.14	19.14	1.93	2.51
	Nm	2.38	0.12	11.24	2.11	2.64
Hole	FC	2.56	0.12	9.46	2.30	2.82
	NC	2.81	0.12	9.69	2.55	3.07
	Nm	2.62	0.12	9.75	2.36	2.89
**Hole presence**	**Contrast**	**Estimate**	**SE**	**DF**	***t*-ratio**	***p***
No hole	F - N	-0.77	0.19	15.62	-4.14	0.002**
	F - Nm	-0.93	0.17	11.66	-5.38	<0.001***
	N - Nm	-0.16	0.18	14.34	-0.86	0.68
Hole	F - N	-0.25	0.17	9.74	-1.51	0.33
	F - Nm	-0.06	0.16	9.31	-0.37	0.93
	N - Nm	0.19	0.16	9.29	1.16	0.51

Table S5. Brood mass at dispersal: interaction between the broods's population of origin and carcass where broods were raised. The table shows least-square means and pairwise differences in brood mass, when larvae originating from FC, NC or Nm populations were raised in FC, NC, or Nm carcasses. The package 'emmeans' was used to calculate least-square (LS) means and perform the pairwise comparisons, using P-value adjustment with Tukey method for multiple comparisons. DF denotes degrees of freedom, calculated with Satterthwaite's approximation. Confidence level (CL) used was 0.95.

**Population**	**Carcass**	**LS means**	**SE**	**DF**	**Lower CL**	**Upper CL**
FC	FC	2.13	0.15	21.66	1.76	2.51
	NC	1.93	0.14	19.29	1.56	2.29
	Nm	1.96	0.15	21.34	1.58	2.34
NC	FC	2.46	0.15	24.32	2.08	2.84
	NC	2.74	0.15	26.08	2.35	3.13
	Nm	2.35	0.15	26.18	1.96	2.74
Nm	FC	2.25	0.15	21.77	1.88	2.63
	NC	2.54	0.14	20.60	2.17	2.91
	Nm	2.71	0.14	18.03	2.35	3.08
**Population**	**Contrast**	**Estimate**	**SE**	**DF**	***t*-ratio**	***p***
FC	FC – NC	0.21	0.19	356.03	1.06	0.54
	FC – Nm	0.17	0.20	357.48	0.87	0.66
	NC – Nm	-0.03	0.19	357.36	-0.18	0.98
NC	FC – NC	-0.28	0.21	356.02	-1.35	0.37
	FC – Nm	0.11	0.21	356.08	0.53	0.86
	NC – Nm	0.39	0.20	356.18	1.93	0.13
Nm	FC – NC	-0.29	0.20	356.79	-1.43	0.32
	FC – Nm	-0.46	0.20	356.34	-2.35	0.05
	NC – Nm	-0.17	0.19	356.22	-0.90	0.64

Table S6. Brood mass at dispersal: interaction between carcass (prepared by FC, NC or Nm parents) and presence of hole. The table shows least-square means and pairwise differences in brood mass between broods raised in FC, NC, and Nm carcasses, with or without a hole present. The package 'emmeans' was used to calculate least-square (LS) means and perform the pairwise comparisons, using P-value adjustment with Tukey method for multiple comparisons. DF denotes degrees of freedom, calculated with Satterthwaite's approximation. Confidence level (CL) used was 0.95. Asterisks denote statistical significance (**P* < 0.05; ***P* < 0.01; ****P* < 0.001).

Carcass	Hole	LS means	SE	DF	Lower CL	Upper CL
FC	No hole	2.11	0.10	41.19	1.91	2.31
	Hole	2.45	0.14	125.27	2.18	2.73
NC	No hole	2.03	0.14	126.08	1.76	2.31
	Hole	2.77	0.10	38.83	2.58	2.97
Nm	No hole	1.91	0.13	111.06	1.64	2.17
	Hole	2.77	0.10	43.19	2.57	2.97

Carcass	Contrast	Estimate	SE	DF	*t*-ratio	*p*
FC	No hole - Hole	-0.34	0.17	357.34	-2.06	0.04*
NC	No hole - Hole	-0.74	0.17	357.77	-4.47	<0.001***
Nm	No hole - Hole	-0.87	0.16	358.82	-5.31	<0.001***

Table S7. Summary of linear regression of brood size on the number of eggs counted at the bottom of breeding boxes. Asterisks denote statistical significance (*P < 0.05; **P < 0.01; ***P < 0.001).

	**Estimate**	**SE**	***t***	***p***
(Intercept)	7.958	1.126	7.066	<0.001***
Number of eggs	0.732	0.060	12.184	<0.001***

Table S8. Variation in mortality between egg-laying and dispersal, approximated as residuals of a linear regression of clutch size on brood size. Least-square means and pairwise differences between populations in the absence and presence of a hole on the carcass are shown. The package 'emmeans' was used to calculate least-square (LS) means and perform the pairwise comparisons, using P-value adjustment with Tukey method for multiple comparisons. DF denotes degrees of freedom, calculated with Satterthwaite's approximation. Confidence level (CL) used was 0.95. Asterisks denote statistical significance (*P < 0.05; **P < 0.01; ***P < 0.001).

**Hole presence**	**Population**	**LS means**	**SE**	**DF**	**Lower CL**	**Upper CL**
No hole	FC	-9.81	1.30	6.62	-12.91	-6.71
	NC	-2.69	1.40	9.07	-5.86	0.48
	Nm	-0.64	1.28	6.33	-3.74	2.45
Hole	FC	2.00	1.24	5.64	-1.10	5.09
	NC	4.44	1.24	5.47	1.34	7.53
	Nm	4.51	1.24	5.60	1.42	7.60
**Hole presence**	**Contrast**	**Estimate**	**SE**	**DF**	***t*-ratio**	***p***
No hole	FC – NC	-7.13	1.91	7.80	-3.73	0.015*
	FC - Nm	-9.17	1.82	6.47	-5.03	0.005**
	NC - Nm	-2.04	1.90	7.64	-1.07	0.56
Hole	FC - NC	-2.44	1.75	5.55	-1.39	0.41
	FC - Nm	-2.51	1.76	5.62	-1.43	0.39
	NC - Nm	-0.07	1.75	5.54	-0.04	1.00

R code for analysis of data presented in:

Duarte, A, Rebar, D, Hallett, AC, Jarrett, BJM, Kilner, RM. Evolutionary change in the construction of the nursery environment when parents are prevented from caring for their young directly. PNAS (2021)

Code by: Ana Duarte

Date: 01/02/2021

Statistical models

Helper functions

We will need helper functions to:

- create graphics of model residuals for model validation
- calculate overdispersion
- obtain legends for graphics

```
# model validation
validatemodel <- function(model, ident){

        fits <- fitted(model)
        deviance.res<- residuals(model, type='deviance')
        Index <- c(1:(length(deviance.res)))

        jpeg(paste('model_valid_',ident,'.jpeg', sep =''))
        layout(matrix(c(1:3), byrow=T, ncol = 3))
        print(qqnorm(deviance.res))
        print(qqline(deviance.res))

        print(plot(deviance.res ~ Index))
        print(plot(deviance.res ~ fits))
        dev.off()

}

# check for overdispersion
overdisp_fun <- function(model) {
  rdf <- df.residual(model)
  rp <- residuals(model,type="pearson")
  Pearson.chisq <- sum(rp^2)
  prat <- Pearson.chisq/rdf
  pval <- pchisq(Pearson.chisq, df=rdf, lower.tail=FALSE)
  c(chisq=Pearson.chisq,ratio=prat,rdf=rdf,p=pval)
}

# obtain legends from graphs
get_legend<-function(myggplot){
  tmp <- ggplot_gtable(ggplot_build(myggplot))
```

```
  leg <- which(sapply(tmp$grobs, function(x) x$name) == "guide-box")
  legend <- tmp$grobs[[leg]]
  return(legend)
}
```

Evolution of the nursery environment.

This section includes the analysis of the data presented in:

- Figure 1
- Table 1

Importing libraries for generalized linear mixed models:

```
library(lme4)
library(lmerTest)
```

Read in the data and do some housekeeping work:

```
dt <- read.csv(file='Duarte_etal_PNAS_fulldata_LYSCORRECT.csv', header=T)
# changing variable names for ease of coding
names(dt)<-c("population", "block", "pair", "male.fam", "female.fam", "carc.w
t", "hole", "roundness", "roundness.adjust", "malesize", "male.lytic", "femsi
ze", "fem.lytic","eggnr", "donor.population", "donor.pair", "donor.crc.wt", "
donor.hole", "donor.roundness", "donor.round.adj",  "broodsize", "broodmass",
"success")

dt$block <- ifelse(dt$block == 1, -0.5, 0.5)
dt$hole.factor<-as.factor(ifelse(dt$hole==0,"No hole", "Hole"))
dt$donor.hole.factor<-as.factor(ifelse(dt$donor.hole==0,"No hole", "Hole"))
dt$donor.hole.factor <- relevel(dt$donor.hole.factor, "No hole")
print(levels(dt$donor.hole.factor))

## [1] "No hole" "Hole"

dt$success.factor<- as.factor(ifelse(dt$success == 0, 'Failure', 'Successful'
))

# Create a population-per-block effect,
# to account for variation between the independent replicates
# due to founder effects and asynchronous maintenance

dt$pop.block.eff <- as.factor(paste(dt$population,dt$block, sep =""))
```

Hole presence

First, we use a Mixed-Effects Generalized Linear Model with binomial distribution, because this is presence/absence data. Since we are using male and female size as covariates, we will only include data points for which we have both.

```
dt.hole<-dt[!is.na(dt$malesize) & !is.na(dt$femsize),]
```

```
hole1 <- glmer(hole ~ population + malesize + femsize + carc.wt + population:
femsize + population:malesize + (1 | pop.block.eff) + (1|male.fam) + (1| fema
le.fam), family='binomial',data = dt.hole)

summary(hole1)

## Generalized linear mixed model fit by maximum likelihood (Laplace
##   Approximation) [glmerMod]
##  Family: binomial  ( logit )
## Formula:
## hole ~ population + malesize + femsize + carc.wt + population:femsize +
##     population:malesize + (1 | pop.block.eff) + (1 | male.fam) +
##     (1 | female.fam)
##    Data: dt.hole
##
##      AIC      BIC   logLik deviance df.resid
##    573.0    625.7   -273.5    547.0      413
##
## Scaled residuals:
##     Min      1Q  Median      3Q     Max
## -1.6108 -0.9768 -0.4394  0.8538  1.8278
##
## Random effects:
##  Groups        Name        Variance  Std.Dev.
##  male.fam      (Intercept) 3.138e-15 5.602e-08
##  female.fam    (Intercept) 4.901e-14 2.214e-07
##  pop.block.eff (Intercept) 0.000e+00 0.000e+00
## Number of obs: 426, groups:  male.fam, 141; female.fam, 140; pop.block.eff
, 6
##
## Fixed effects:
##                         Estimate Std. Error z value Pr(>|z|)
## (Intercept)               1.7079     3.9088   0.437   0.6622
## populationNC             -8.5028     4.4584  -1.907   0.0565 .
## populationNm             -7.8337     4.6221  -1.695   0.0901 .
## malesize                 -0.6417     0.4397  -1.459   0.1445
## femsize                  -0.5507     0.5331  -1.033   0.3016
## carc.wt                   0.2276     0.1294   1.759   0.0786 .
## populationNC:femsize      1.3188     0.7416   1.778   0.0753 .
## populationNm:femsize      0.9948     0.7102   1.401   0.1613
## populationNC:malesize     0.8583     0.6102   1.407   0.1595
## populationNm:malesize     0.9797     0.6155   1.592   0.1114
## ---
## Signif. codes:  0 '***' 0.001 '**' 0.01 '*' 0.05 '.' 0.1 ' ' 1
##
## Correlation of Fixed Effects:
##             (Intr) ppltNC ppltnN malesz femsiz crc.wt ppltnNC:f ppltnNm:f
## populatinNC -0.700
## populatinNm -0.679  0.599
## malesize    -0.654  0.576  0.556
```

```
## femsize     -0.756  0.652  0.630  0.228
## carc.wt     -0.439 -0.017 -0.008 -0.007  0.025
## ppltnNC:fms  0.539 -0.791 -0.453 -0.164 -0.718 -0.008
## ppltnNm:fms  0.560 -0.490 -0.799 -0.171 -0.750 -0.002  0.539
## ppltnNC:mls  0.453 -0.661 -0.401 -0.721 -0.163  0.046  0.067     0.123
## ppltnNm:mls  0.463 -0.412 -0.720 -0.714 -0.163  0.015  0.117     0.161
##             ppltnNC:m
## populatinNC
## populatinNm
## malesize
## femsize
## carc.wt
## ppltnNC:fms
## ppltnNm:fms
## ppltnNC:mls
## ppltnNm:mls  0.515
## optimizer (Nelder_Mead) convergence code: 0 (OK)
## boundary (singular) fit: see ?isSingular
```

The random effects explain almost no variance, hence we remove these and try a GLM. We do model selection by comparing nested models with anova. If the models differ significantly and there is a reduction of AIC by at least 2 points, we select the model with lowest AIC.

```
hole2.glm <- glm(hole ~ population + malesize + femsize + carc.wt + populatio
n:femsize + population:malesize , family=binomial(link='logit'),data = dt.hol
e, na.action = na.omit)
hole3.glm <- glm(hole ~ population + malesize + femsize + carc.wt ,family=bin
omial(link='logit'),data = dt.hole, na.action = na.omit)
anova(hole2.glm, hole3.glm, test="Chisq")
```

```
## Analysis of Deviance Table
##
## Model 1: hole ~ population + malesize + femsize + carc.wt + population:fem
size +
##     population:malesize
## Model 2: hole ~ population + malesize + femsize + carc.wt
##   Resid. Df Resid. Dev Df Deviance Pr(>Chi)
## 1       416     547.03
## 2       420     552.74 -4  -5.7064   0.2222
```

```
AIC(hole2.glm, hole3.glm)
```

```
##           df      AIC
## hole2.glm 10 567.0340
## hole3.glm  6 564.7404
```

```
hole4.glm <- glm(hole ~ population + femsize + carc.wt , family=binomial(link
="logit"),data = dt.hole, na.action=na.omit)
anova(hole3.glm, hole4.glm, test="Chisq")
```

```
## Analysis of Deviance Table
##
## Model 1: hole ~ population + malesize + femsize + carc.wt
## Model 2: hole ~ population + femsize + carc.wt
##   Resid. Df Resid. Dev Df  Deviance Pr(>Chi)
## 1       420     552.74
## 2       421     552.76 -1 -0.021674    0.883
```

```
AIC(hole3.glm,hole4.glm)
```

```
##           df      AIC
## hole3.glm  6 564.7404
## hole4.glm  5 562.7621
```

`hole3.glm` is the minimal adequate model, as removal of male size does not reduce AIC by more than 2 units. Examine plots of residuals and obtain the global effect of population by comparing nested model without that fixed effect. In main text: reported in Table 1.

```
validatemodel(hole3.glm, 'hole3')  # will produce graphics for inspection of residuals

hole5.glm <- glm(hole ~ malesize + femsize + carc.wt , family='binomial',data = dt)
effect.population <- anova(hole3.glm, hole5.glm, test='Chisq')
effect.population
```

```
## Analysis of Deviance Table
##
## Model 1: hole ~ population + malesize + femsize + carc.wt
## Model 2: hole ~ malesize + femsize + carc.wt
##   Resid. Df Resid. Dev Df Deviance  Pr(>Chi)
## 1       420     552.74
## 2       422     586.06 -2  -33.321 5.814e-08 ***
## ---
## Signif. codes:  0 '***' 0.001 '**' 0.01 '*' 0.05 '.' 0.1 ' ' 1
```

Checking if the mixed effect population-per-block would change the results quantitatively.

```
hole3.glmer <- glmer(hole ~ population + malesize + femsize + carc.wt + (1 | pop.block.eff) ,family=binomial(link='logit'),data = dt, na.action = na.omit)
summary(hole3.glmer)
```

```
## Generalized linear mixed model fit by maximum likelihood (Laplace
##   Approximation) [glmerMod]
##  Family: binomial  ( logit )
## Formula: hole ~ population + malesize + femsize + carc.wt + (1 | pop.block.eff)
##    Data: dt
##
##      AIC      BIC   logLik deviance df.resid
##    566.7    595.1   -276.4    552.7      419
##
```

```
## Scaled residuals:
##     Min      1Q  Median      3Q     Max
## -1.4483 -1.0552 -0.5242  0.8339  1.8349
##
## Random effects:
##  Groups        Name        Variance Std.Dev.
##  pop.block.eff (Intercept) 0        0
## Number of obs: 426, groups:  pop.block.eff, 6
##
## Fixed effects:
##                Estimate Std. Error z value Pr(>|z|)
## (Intercept)  -5.29847    2.52729  -2.097   0.0360 *
## populationNC  1.35688    0.27455   4.942 7.72e-07 ***
## populationNm  1.22230    0.25317   4.828 1.38e-06 ***
## malesize      0.03628    0.24647   0.147   0.8830
## femsize       0.29910    0.28870   1.036   0.3002
## carc.wt       0.22561    0.12867   1.753   0.0795 .
## ---
## Signif. codes:  0 '***' 0.001 '**' 0.01 '*' 0.05 '.' 0.1 ' ' 1
##
## Correlation of Fixed Effects:
##              (Intr) ppltNC ppltnN malesz femsiz
## populatinNC -0.373
## populatinNm -0.156  0.537
## malesize    -0.505  0.280  0.064
## femsize     -0.587  0.289  0.142  0.060
## carc.wt     -0.697  0.070  0.000  0.037  0.046
## optimizer (Nelder_Mead) convergence code: 0 (OK)
## boundary (singular) fit: see ?isSingular
```

Obtain the proportion of carcasses with hole per population.

```
hole_prop <- prop.table(table(dt$hole, dt$population),2)
hole_prop

##
##             FC        NC        Nm
##   0 0.6986301 0.3943662 0.4137931
##   1 0.3013699 0.6056338 0.5862069
```

Carcass roundness

Start by fitting a GLMM with population-per-block, male family and female family as mixed effects. Continue with model selection from that first model, as above. Presented in Table 1.

```
round1<- lmer(roundness.adjust ~ population + malesize + femsize + carc.wt +
population:malesize + population:femsize + (1|pop.block.eff) + (1|male.fam) +
(1| female.fam),
          data = dt, na.action=na.omit)
summary(round1)
```

```
## Linear mixed model fit by REML. t-tests use Satterthwaite's method [
## lmerModLmerTest]
## Formula: roundness.adjust ~ population + malesize + femsize + carc.wt +
##     population:malesize + population:femsize + (1 | pop.block.eff) +
##     (1 | male.fam) + (1 | female.fam)
##    Data: dt
##
## REML criterion at convergence: -647.2
##
## Scaled residuals:
##     Min      1Q  Median      3Q     Max
## -3.4090 -0.6307  0.2183  0.7908  1.7332
##
## Random effects:
##  Groups        Name        Variance  Std.Dev.
##  male.fam      (Intercept) 0.0000000 0.00000
##  female.fam    (Intercept) 0.0000000 0.00000
##  pop.block.eff (Intercept) 0.0001389 0.01179
##  Residual                  0.0112065 0.10586
## Number of obs: 426, groups:  male.fam, 141; female.fam, 140; pop.block.eff
, 6
##
## Fixed effects:
##                        Estimate Std. Error         df t value Pr(>|t|)
## (Intercept)           -0.341437   0.192953 314.685939  -1.770 0.077773 .
## populationNC           0.960308   0.225441 343.041880   4.260 2.65e-05 **
*
## populationNm           1.231346   0.234806 349.509604   5.244 2.73e-07 **
*
## malesize               0.137631   0.021862 339.346693   6.295 9.48e-10 **
*
## femsize                0.099294   0.026250 373.683648   3.783 0.000181 **
*
## carc.wt                0.001584   0.005875 415.026911   0.270 0.787632
## populationNC:malesize  -0.097540   0.030822 400.032533  -3.165 0.001671 **
## populationNm:malesize  -0.145092   0.031260 399.191373  -4.641 4.70e-06 **
*
## populationNC:femsize   -0.099171   0.037226 408.880022  -2.664 0.008027 **
## populationNm:femsize   -0.108648   0.035723 406.345960  -3.041 0.002508 **
## ---
## Signif. codes:  0 '***' 0.001 '**' 0.01 '*' 0.05 '.' 0.1 ' ' 1
##
## Correlation of Fixed Effects:
##             (Intr) ppltNC ppltnN malesz femsiz crc.wt ppltnNC:m ppltnNm:m
## populatinNC -0.707
## populatinNm -0.684  0.585
## malesize    -0.675  0.578  0.555
## femsize     -0.769  0.647  0.621  0.245
## carc.wt     -0.410 -0.011  0.002 -0.001  0.031
## ppltnNC:mls  0.456 -0.662 -0.394 -0.709 -0.172  0.057
```

```
## ppltnNm:mls  0.470 -0.404 -0.723 -0.699 -0.171  0.006  0.496
## ppltnNC:fms  0.545 -0.787 -0.438 -0.173 -0.705 -0.027  0.066     0.121
## ppltnNm:fms  0.560 -0.476 -0.796 -0.180 -0.734 -0.010  0.127     0.163
##             ppltnNC:f
## populatinNC
## populatinNm
## malesize
## femsize
## carc.wt
## ppltnNC:mls
## ppltnNm:mls
## ppltnNC:fms
## ppltnNm:fms  0.518
## optimizer (nloptwrap) convergence code: 0 (OK)
## boundary (singular) fit: see ?isSingular
```

```
# random effects male and female ID are not explaining any variance
# leave pop.block.eff in

round2<- lmer(roundness.adjust ~ population + malesize + femsize + carc.wt +
population:malesize + population:femsize + (1| pop.block.eff) ,
          data = dt, na.action=na.omit)
summary(round2)
```

```
## Linear mixed model fit by REML. t-tests use Satterthwaite's method [
## lmerModLmerTest]
## Formula: roundness.adjust ~ population + malesize + femsize + carc.wt +
##     population:malesize + population:femsize + (1 | pop.block.eff)
##    Data: dt
##
## REML criterion at convergence: -647.2
##
## Scaled residuals:
##     Min      1Q  Median      3Q     Max
## -3.4090 -0.6307  0.2183  0.7908  1.7332
##
## Random effects:
##  Groups        Name        Variance  Std.Dev.
##  pop.block.eff (Intercept) 0.0001389 0.01179
##  Residual                  0.0112065 0.10586
## Number of obs: 426, groups:  pop.block.eff, 6
##
## Fixed effects:
##                         Estimate Std. Error         df t value Pr(>|t|)
## (Intercept)            -0.341437   0.192953 314.685629  -1.770 0.077773 .
## populationNC            0.960307   0.225441 343.041723   4.260 2.65e-05 **
*
## populationNm            1.231345   0.234806 349.509457   5.244 2.73e-07 **
*
## malesize                0.137631   0.021862 339.346372   6.295 9.48e-10 **
```

```
*
## femsize                  0.099294   0.026250 373.683436   3.783 0.000181 **
*
## carc.wt                  0.001584   0.005875 415.026908   0.270 0.787632
## populationNC:malesize  -0.097540   0.030822 400.032459  -3.165 0.001671 **
## populationNm:malesize  -0.145092   0.031260 399.191298  -4.641 4.70e-06 **
*
## populationNC:femsize   -0.099171   0.037226 408.879981  -2.664 0.008027 **
## populationNm:femsize   -0.108648   0.035723 406.345909  -3.041 0.002508 **
## ---
## Signif. codes:  0 '***' 0.001 '**' 0.01 '*' 0.05 '.' 0.1 ' ' 1
##
## Correlation of Fixed Effects:
##             (Intr) ppltNC ppltnN malesz femsiz crc.wt ppltnNC:m ppltnNm:m
## populatinNC -0.707
## populatinNm -0.684  0.585
## malesize    -0.675  0.578  0.555
## femsize     -0.769  0.647  0.621  0.245
## carc.wt     -0.410 -0.011  0.002 -0.001  0.031
## ppltnNC:mls  0.456 -0.662 -0.394 -0.709 -0.172  0.057
## ppltnNm:mls  0.470 -0.404 -0.723 -0.699 -0.171  0.006  0.496
## ppltnNC:fms  0.545 -0.787 -0.438 -0.173 -0.705 -0.027  0.066     0.121
## ppltnNm:fms  0.560 -0.476 -0.796 -0.180 -0.734 -0.010  0.127     0.163
##             ppltnNC:f
## populatinNC
## populatinNm
## malesize
## femsize
## carc.wt
## ppltnNC:mls
## ppltnNm:mls
## ppltnNC:fms
## ppltnNm:fms  0.518
```

```
anova(round1, round2)
```

```
## Data: dt
## Models:
## round2: roundness.adjust ~ population + malesize + femsize + carc.wt + pop
ulation:malesize + population:femsize + (1 | pop.block.eff)
## round1: roundness.adjust ~ population + malesize + femsize + carc.wt + pop
ulation:malesize + population:femsize + (1 | pop.block.eff) + (1 | male.fam)
+ (1 | female.fam)
##        npar     AIC     BIC logLik deviance Chisq Df Pr(>Chisq)
## round2   12 -687.93 -639.28 355.97  -711.93
## round1   14 -683.93 -627.17 355.97  -711.93     0  2          1
```

```
round3 <- lmer(roundness.adjust ~ population + malesize + femsize + populatio
n:malesize + population:femsize + (1| pop.block.eff) ,
```

```
         data = dt, na.action=na.omit)
summary(round3)

## Linear mixed model fit by REML. t-tests use Satterthwaite's method [
## lmerModLmerTest]
## Formula:
## roundness.adjust ~ population + malesize + femsize + population:malesize +
##     population:femsize + (1 | pop.block.eff)
##    Data: dt
##
## REML criterion at convergence: -655.6
##
## Scaled residuals:
##     Min      1Q  Median      3Q     Max
## -3.4072 -0.6317  0.2110  0.7913  1.7353
##
## Random effects:
##  Groups        Name        Variance  Std.Dev.
##  pop.block.eff (Intercept) 0.0001433 0.01197
##  Residual                  0.0111803 0.10574
## Number of obs: 426, groups:  pop.block.eff, 6
##
## Fixed effects:
##                          Estimate Std. Error        df t value Pr(>|t|)
## (Intercept)              -0.32111    0.17585 271.68659  -1.826  0.06894 .
## populationNC              0.96196    0.22524 345.77230   4.271 2.52e-05 ***
## populationNm              1.23239    0.23461 351.90567   5.253 2.60e-07 ***
## malesize                  0.13775    0.02184 342.53341   6.306 8.85e-10 ***
## femsize                   0.09918    0.02621 375.39099   3.784  0.00018 ***
## populationNC:malesize    -0.09812    0.03074 402.10579  -3.192  0.00152 **
## populationNm:malesize    -0.14527    0.03123 400.94797  -4.652 4.48e-06 ***
## populationNC:femsize     -0.09900    0.03717 410.05684  -2.663  0.00804 **
## populationNm:femsize     -0.10868    0.03569 407.63819  -3.045  0.00247 **
## ---
## Signif. codes:  0 '***' 0.001 '**' 0.01 '*' 0.05 '.' 0.1 ' ' 1
##
## Correlation of Fixed Effects:
##             (Intr) ppltNC ppltnN malesz femsiz ppltnNC:m ppltnNm:m ppltnNC
:f
## populatinNC -0.781
## populatinNm -0.750  0.585
## malesize    -0.741  0.578  0.555
## femsize     -0.830  0.648  0.622  0.246
## ppltnNC:mls  0.527 -0.662 -0.395 -0.711 -0.175
## ppltnNm:mls  0.518 -0.405 -0.723 -0.699 -0.172  0.497
## ppltnNC:fms  0.585 -0.788 -0.439 -0.173 -0.705  0.068     0.121
## ppltnNm:fms  0.609 -0.476 -0.796 -0.181 -0.735  0.128     0.164     0.518

anova(round2, round3)
```

```
## Data: dt
## Models:
## round3: roundness.adjust ~ population + malesize + femsize + population:ma
lesize + population:femsize + (1 | pop.block.eff)
## round2: roundness.adjust ~ population + malesize + femsize + carc.wt + pop
ulation:malesize + population:femsize + (1 | pop.block.eff)
##        npar     AIC     BIC logLik deviance Chisq Df Pr(>Chisq)
## round3   11 -689.82 -645.22 355.91  -711.82
## round2   12 -687.93 -639.28 355.97  -711.93 0.113  1     0.7367
```

round2 is the minimal adequate model. We can validate the model by checking the residuals.

```
validatemodel(round2, ident="round2")
```

Let us test the effect of interactions by running nested models without the interactions.

```
round2.1 <- update(round2, ~.- population:malesize)
round2.2 <- update(round2, ~.- population:femsize)
anova(round2, round2.1)

## Data: dt
## Models:
## round2.1: roundness.adjust ~ population + malesize + femsize + carc.wt + (
1 | pop.block.eff) + population:femsize
## round2: roundness.adjust ~ population + malesize + femsize + carc.wt + pop
ulation:malesize + population:femsize + (1 | pop.block.eff)
##          npar     AIC     BIC logLik deviance  Chisq Df Pr(>Chisq)
## round2.1   10 -671.14 -630.60 345.57  -691.14
## round2     12 -687.93 -639.28 355.97  -711.93 20.791  2  3.057e-05 ***
## ---
## Signif. codes:  0 '***' 0.001 '**' 0.01 '*' 0.05 '.' 0.1 ' ' 1

anova(round2, round2.2)

## Data: dt
## Models:
## round2.2: roundness.adjust ~ population + malesize + femsize + carc.wt + (
1 | pop.block.eff) + population:malesize
## round2: roundness.adjust ~ population + malesize + femsize + carc.wt + pop
ulation:malesize + population:femsize + (1 | pop.block.eff)
##          npar     AIC     BIC logLik deviance  Chisq Df Pr(>Chisq)
## round2.2   10 -682.08 -641.54 351.04  -702.08
## round2     12 -687.93 -639.28 355.97  -711.93 9.8517  2   0.007257 **
## ---
## Signif. codes:  0 '***' 0.001 '**' 0.01 '*' 0.05 '.' 0.1 ' ' 1
```

Check if the model gives same qualitative and p-values if random effect population-per-block not included.

```
round2.lm<- lm(roundness.adjust ~ population + malesize + femsize + carc.wt + 
population:malesize + population:femsize ,
          data = dt, na.action=na.omit)
```

Obtaining overall effect sizes for fixed effects.

```
round2.population <- update(round2, ~.- population)
aov.population <- anova(round2, round2.population)

round2.malesize <- update(round2, ~.- malesize)
aov.malesize <- anova(round2, round2.malesize)

round2.femsize <- update(round2, ~.- femsize)
aov.femsize <- anova(round2, round2.femsize)

round2.carcass <- lmer(roundness.adjust ~ population + malesize + femsize + p
opulation:malesize + population:femsize + (1| pop.block.eff) ,
          data = dt, na.action=na.omit)

aov.carcass <- anova(round2, round2.carcass)
```

Lytic activity females

We include only pairs for which I have male and female, as well as female size data because partner lytic activity is an important covariate in model.

```
dt.2<- dt[!is.na(dt$fem.lytic) & !is.na(dt$male.lytic),]
```

First we try untransformed lytic activity values with a GLMM. Carry out model selection as above, then validate the model.

```
femlys.1 <- lmer(fem.lytic ~ population * femsize + male.lytic + carc.wt  + 
(1|pop.block.eff) + (1| female.fam), data = dt.2)
summary(femlys.1)

## Linear mixed model fit by REML. t-tests use Satterthwaite's method [
## lmerModLmerTest]
## Formula: fem.lytic ~ population * femsize + male.lytic + carc.wt + (1 |
##     pop.block.eff) + (1 | female.fam)
##    Data: dt.2
## 
## REML criterion at convergence: 4802.2
## 
## Scaled residuals: 
##     Min      1Q  Median      3Q     Max 
## -1.9376 -0.5861 -0.1879  0.4485  4.7073 
## 
## Random effects:
##  Groups        Name        Variance Std.Dev.
##  female.fam    (Intercept)  1800     42.43  
##  pop.block.eff (Intercept)  2373     48.71  
```

```
##  Residual                  44754    211.55
## Number of obs: 358, groups:  female.fam, 137; pop.block.eff, 6
##
## Fixed effects:
##                        Estimate Std. Error         df t value Pr(>|t|)
## (Intercept)          -197.09249  339.13506  319.20710  -0.581   0.5615
## populationNC          224.93358  391.55575  232.70237   0.574   0.5662
## populationNm          -80.72736  363.32810  248.45275  -0.222   0.8243
## femsize                99.12730   57.38359  249.46140   1.727   0.0853 .
## male.lytic              0.35456    0.06238  328.25594   5.684 2.91e-08 ***
## carc.wt                -4.48477   16.15695  341.96589  -0.278   0.7815
## populationNC:femsize  -46.46197   85.65083  233.98586  -0.542   0.5880
## populationNm:femsize   22.50806   78.23048  252.26503   0.288   0.7738
## ---
## Signif. codes:  0 '***' 0.001 '**' 0.01 '*' 0.05 '.' 0.1 ' ' 1
##
## Correlation of Fixed Effects:
##              (Intr) ppltNC ppltnN femsiz ml.lyt crc.wt pplNC:
## populatinNC -0.537
## populatinNm -0.574  0.506
## femsize     -0.783  0.677  0.733
## male.lytic   0.003  0.036 -0.056 -0.033
## carc.wt     -0.610 -0.013 -0.016  0.003 -0.052
## ppltnNC:fms  0.517 -0.989 -0.488 -0.668 -0.037  0.014
## ppltnNm:fms  0.565 -0.495 -0.988 -0.735  0.064  0.011  0.489
```

```
femlys.2 <- lmer(fem.lytic ~ population + femsize + male.lytic + carc.wt +
(1|pop.block.eff) +(1| female.fam), data = dt.2)
summary(femlys.2)
```

```
## Linear mixed model fit by REML. t-tests use Satterthwaite's method [
## lmerModLmerTest]
## Formula: fem.lytic ~ population + femsize + male.lytic + carc.wt + (1 |
##     pop.block.eff) + (1 | female.fam)
##    Data: dt.2
##
## REML criterion at convergence: 4823.9
##
## Scaled residuals:
##     Min      1Q  Median      3Q     Max
## -1.9232 -0.5997 -0.1809  0.4371  4.6860
##
## Random effects:
##  Groups        Name        Variance Std.Dev.
##  female.fam    (Intercept)  2042     45.19
##  pop.block.eff (Intercept)  2345     48.43
##  Residual                  44368    210.64
## Number of obs: 358, groups:  female.fam, 137; pop.block.eff, 6
##
## Fixed effects:
```

```
##                  Estimate Std. Error         df t value Pr(>|t|)
## (Intercept)  -177.14495  263.21313  335.77780  -0.673  0.50140
## populationNC   18.29194   57.17234    3.01253   0.320  0.76992
## populationNm   20.81707   56.43910    2.86121   0.369  0.73784
## femsize        94.96892   33.19956  250.38246   2.861  0.00459 **
## male.lytic      0.34992    0.06191  330.39899   5.652 3.42e-08 ***
## carc.wt        -4.42907   16.11779  342.86869  -0.275  0.78364
## ---
## Signif. codes:  0 '***' 0.001 '**' 0.01 '*' 0.05 '.' 0.1 ' ' 1
##
## Correlation of Fixed Effects:
##             (Intr) ppltNC ppltnN femsiz ml.lyt
## populatinNC -0.180
## populatinNm -0.117  0.502
## femsize     -0.602  0.116  0.052
## male.lytic  -0.015  0.000  0.046 -0.024
## carc.wt     -0.795  0.008 -0.032  0.025 -0.052
```

```
anova(femlys.1,femlys.2)
```

```
## Data: dt.2
## Models:
## femlys.2: fem.lytic ~ population + femsize + male.lytic + carc.wt + (1 | p
op.block.eff) + (1 | female.fam)
## femlys.1: fem.lytic ~ population * femsize + male.lytic + carc.wt + (1 | p
op.block.eff) + (1 | female.fam)
##          npar    AIC    BIC  logLik deviance  Chisq Df Pr(>Chisq)
## femlys.2    9 4880.8 4915.7 -2431.4   4862.8
## femlys.1   11 4884.1 4926.8 -2431.1   4862.1 0.6868  2     0.7093
```

```
femlys.3 <- lmer(fem.lytic ~ population + femsize + male.lytic +   (1|pop.blo
ck.eff) +(1| female.fam), data = dt.2)
summary(femlys.3)
```

```
## Linear mixed model fit by REML. t-tests use Satterthwaite's method [
## lmerModLmerTest]
## Formula: fem.lytic ~ population + femsize + male.lytic + (1 | pop.block.ef
f) +
##     (1 | female.fam)
##    Data: dt.2
##
## REML criterion at convergence: 4831.4
##
## Scaled residuals:
##     Min      1Q  Median      3Q     Max
## -1.9322 -0.5983 -0.1823  0.4419  4.6810
##
## Random effects:
##  Groups        Name        Variance Std.Dev.
##  female.fam    (Intercept)  2051     45.28
##  pop.block.eff (Intercept)  2410     49.09
```

```
##  Residual                  44231    210.31
## Number of obs: 358, groups:  female.fam, 137; pop.block.eff, 6
##
## Fixed effects:
##                Estimate Std. Error         df t value Pr(>|t|)
## (Intercept)  -234.40975  159.40927  199.93675  -1.470  0.14300
## populationNC   18.41691   57.71944    3.03716   0.319  0.77036
## populationNm   20.31448   56.96848    2.88261   0.357  0.74589
## femsize        95.15509   33.14703  250.77300   2.871  0.00445 **
## male.lytic      0.34875    0.06175  333.13735   5.648 3.48e-08 ***
## ---
## Signif. codes:  0 '***' 0.001 '**' 0.01 '*' 0.05 '.' 0.1 ' ' 1
##
## Correlation of Fixed Effects:
##             (Intr) ppltNC ppltnN femsiz
## populatinNC -0.288
## populatinNm -0.235  0.503
## femsize     -0.961  0.114  0.052
## male.lytic  -0.093  0.001  0.044 -0.022
```

```
anova(femlys.2,femlys.3)
```

```
## Data: dt.2
## Models:
## femlys.3: fem.lytic ~ population + femsize + male.lytic + (1 | pop.block.e
ff) + (1 | female.fam)
## femlys.2: fem.lytic ~ population + femsize + male.lytic + carc.wt + (1 | p
op.block.eff) + (1 | female.fam)
##          npar    AIC    BIC  logLik deviance  Chisq Df Pr(>Chisq)
## femlys.3    8 4879.0 4910.0 -2431.5   4863.0
## femlys.2    9 4880.8 4915.7 -2431.4   4862.8 0.1536  1     0.6951
```

```
femlys.4 <- lmer(fem.lytic ~ femsize + male.lytic +   (1|pop.block.eff) + (1|
female.fam), data = dt.2)
summary(femlys.4)
```

```
## Linear mixed model fit by REML. t-tests use Satterthwaite's method [
## lmerModLmerTest]
## Formula: fem.lytic ~ femsize + male.lytic + (1 | pop.block.eff) + (1 |
##     female.fam)
##    Data: dt.2
##
## REML criterion at convergence: 4850.7
##
## Scaled residuals:
##     Min      1Q  Median      3Q     Max
## -1.9051 -0.6151 -0.1960  0.4238  4.7284
##
## Random effects:
##  Groups        Name        Variance Std.Dev.
##  female.fam    (Intercept)  2014     44.88
```

```
##  pop.block.eff (Intercept)  1185      34.42
##  Residual                  44267     210.40
## Number of obs: 358, groups:  female.fam, 137; pop.block.eff, 6
##
## Fixed effects:
##               Estimate Std. Error         df t value Pr(>|t|)
## (Intercept) -217.44925  150.36389  249.90348  -1.446  0.14939
## femsize       93.83039   32.77784  253.70721   2.863  0.00455 **
## male.lytic     0.35563    0.06127  330.69180   5.804 1.52e-08 ***
## ---
## Signif. codes:  0 '***' 0.001 '**' 0.01 '*' 0.05 '.' 0.1 ' ' 1
##
## Correlation of Fixed Effects:
##            (Intr) femsiz
## femsize    -0.986
## male.lytic -0.090 -0.024
```

```
anova(femlys.3,femlys.4)
```

```
## Data: dt.2
## Models:
## femlys.4: fem.lytic ~ femsize + male.lytic + (1 | pop.block.eff) + (1 | fe
male.fam)
## femlys.3: fem.lytic ~ population + femsize + male.lytic + (1 | pop.block.e
ff) + (1 | female.fam)
##          npar    AIC    BIC  logLik deviance Chisq Df Pr(>Chisq)
## femlys.4    6 4875.3 4898.5 -2431.6   4863.3
## femlys.3    8 4879.0 4910.0 -2431.5   4863.0 0.315  2     0.8543
```

```
validatemodel(femlys.4, ident='femlys.4')
```

Residuals do not look good, so we use **log-transformed** lytic activity values. Results presented in Table 1 in main text.

```
femloglys.1 <- lmer(log(fem.lytic) ~ population * femsize + log(male.lytic) +
carc.wt +   (1|pop.block.eff) + (1| female.fam), data = dt.2)
summary(femloglys.1)
```

```
## Linear mixed model fit by REML. t-tests use Satterthwaite's method [
## lmerModLmerTest]
## Formula: log(fem.lytic) ~ population * femsize + log(male.lytic) + carc.wt
+
##     (1 | pop.block.eff) + (1 | female.fam)
##    Data: dt.2
##
## REML criterion at convergence: 781.3
##
## Scaled residuals:
##     Min      1Q  Median      3Q     Max
## -3.8881 -0.5619  0.0373  0.7075  2.5907
##
```

```
## Random effects:
##  Groups        Name        Variance Std.Dev.
##  female.fam    (Intercept) 0.000000 0.00000
##  pop.block.eff (Intercept) 0.009868 0.09934
##  Residual                  0.493893 0.70278
## Number of obs: 358, groups:  female.fam, 137; pop.block.eff, 6
##
## Fixed effects:
##                        Estimate Std. Error        df t value Pr(>|t|)
## (Intercept)             2.30487    1.12824 349.17600   2.043  0.04181 *
## populationNC            1.19159    1.24991 347.05950   0.953  0.34108
## populationNm            0.67254    1.15874 345.66738   0.580  0.56202
## femsize                 0.52511    0.18399 349.97520   2.854  0.00457 **
## log(male.lytic)         0.27734    0.05649 328.19722   4.910 1.44e-06 ***
## carc.wt                -0.05917    0.05280 349.97063  -1.121  0.26326
## populationNC:femsize   -0.26025    0.27469 349.45871  -0.947  0.34406
## populationNm:femsize   -0.13088    0.25081 349.47334  -0.522  0.60211
## ---
## Signif. codes:  0 '***' 0.001 '**' 0.01 '*' 0.05 '.' 0.1 ' ' 1
##
## Correlation of Fixed Effects:
##             (Intr) ppltNC ppltnN femsiz lg(m.) crc.wt pplNC:
## populatinNC -0.529
## populatinNm -0.548  0.507
## femsize     -0.761  0.682  0.735
## lg(ml.lytc) -0.248  0.056 -0.016  0.017
## carc.wt     -0.583 -0.014 -0.016  0.002 -0.067
## ppltnNC:fms  0.512 -0.994 -0.492 -0.670 -0.054  0.015
## ppltnNm:fms  0.540 -0.499 -0.993 -0.733  0.026  0.011  0.490
## optimizer (nloptwrap) convergence code: 0 (OK)
## boundary (singular) fit: see ?isSingular
```

```
femloglys.2 <- lmer(log(fem.lytic) ~ population + femsize + log(male.lytic) +
carc.wt +   (1|pop.block.eff) , data = dt.2)
summary(femloglys.2)
```

```
## Linear mixed model fit by REML. t-tests use Satterthwaite's method [
## lmerModLmerTest]
## Formula: log(fem.lytic) ~ population + femsize + log(male.lytic) + carc.wt
+
##     (1 | pop.block.eff)
##    Data: dt.2
##
## REML criterion at convergence: 780.2
##
## Scaled residuals:
##     Min      1Q  Median      3Q     Max
## -3.8641 -0.5666  0.0588  0.7159  2.5796
##
## Random effects:
```

```
##  Groups        Name        Variance Std.Dev.
##  pop.block.eff (Intercept) 0.009669 0.09833
##  Residual                  0.492391 0.70171
## Number of obs: 358, groups:  pop.block.eff, 6
##
## Fixed effects:
##                  Estimate Std. Error        df t value Pr(>|t|)
## (Intercept)       2.87697    0.89252 350.67144   3.223 0.001386 **
## populationNC      0.01354    0.13676   3.10527   0.099 0.927169
## populationNm      0.06663    0.13418   2.87624   0.497 0.654929
## femsize           0.40262    0.10637 351.09917   3.785 0.000181 ***
## log(male.lytic)   0.27482    0.05621 327.77227   4.889 1.59e-06 ***
## carc.wt          -0.05847    0.05271 351.97870  -1.109 0.268132
## ---
## Signif. codes:  0 '***' 0.001 '**' 0.01 '*' 0.05 '.' 0.1 ' ' 1
##
## Correlation of Fixed Effects:
##             (Intr) ppltNC ppltnN femsiz lg(m.)
## populatinNC -0.177
## populatinNm -0.109  0.508
## femsize     -0.575  0.155  0.071
## lg(ml.lytc) -0.305  0.029  0.081  0.015
## carc.wt     -0.747  0.010 -0.046  0.024 -0.066
```

```
# with both female.fam and pop.block.eff as random effects, the model doesn't
converge.
# in this case it's more important to keep the population effect, because tha
t is what we are testing for
# does not change effect sizes of fixed terms

femloglys.3 <- lmer(log(fem.lytic) ~ population + femsize + log(male.lytic) +
(1|pop.block.eff) , data = dt.2)
summary(femloglys.3)
```

```
## Linear mixed model fit by REML. t-tests use Satterthwaite's method [
## lmerModLmerTest]
## Formula: log(fem.lytic) ~ population + femsize + log(male.lytic) + (1 |
##     pop.block.eff)
##    Data: dt.2
##
## REML criterion at convergence: 777.4
##
## Scaled residuals:
##     Min      1Q  Median      3Q     Max
## -3.8701 -0.5504  0.0595  0.6981  2.6382
##
## Random effects:
##  Groups        Name        Variance Std.Dev.
##  pop.block.eff (Intercept) 0.01175  0.1084
##  Residual                  0.49223  0.7016
```

```
## Number of obs: 358, groups:  pop.block.eff, 6
##
## Fixed effects:
##                   Estimate Std. Error        df t value Pr(>|t|)
## (Intercept)        2.14818    0.59444 309.28599   3.614 0.000352 ***
## populationNC       0.01513    0.14414   3.13414   0.105 0.922781
## populationNm       0.05961    0.14156   2.91559   0.421 0.702789
## femsize            0.40500    0.10635 351.98490   3.808 0.000165 ***
## log(male.lytic)    0.26914    0.05617 337.02116   4.792 2.48e-06 ***
## ---
## Signif. codes:  0 '***' 0.001 '**' 0.01 '*' 0.05 '.' 0.1 ' ' 1
##
## Correlation of Fixed Effects:
##              (Intr) ppltNC ppltnN femsiz
## populatinNC -0.254
## populatinNm -0.216  0.508
## femsize     -0.837  0.147  0.069
## lg(ml.lytc) -0.534  0.028  0.075  0.017
```

```
anova(femloglys.2, femloglys.3)
```

```
## Data: dt.2
## Models:
## femloglys.3: log(fem.lytic) ~ population + femsize + log(male.lytic) + (1
| pop.block.eff)
## femloglys.2: log(fem.lytic) ~ population + femsize + log(male.lytic) + car
c.wt + (1 | pop.block.eff)
##             npar    AIC    BIC  logLik deviance  Chisq Df Pr(>Chisq)
## femloglys.3    7 775.28 802.44 -380.64   761.28
## femloglys.2    8 775.68 806.73 -379.84   759.68 1.5908  1     0.2072
```

```
femloglys.4 <- lmer(log(fem.lytic) ~ femsize + log(male.lytic) +   (1|pop.blo
ck.eff) , data = dt.2)
summary(femloglys.4)
```

```
## Linear mixed model fit by REML. t-tests use Satterthwaite's method [
## lmerModLmerTest]
## Formula: log(fem.lytic) ~ femsize + log(male.lytic) + (1 | pop.block.eff)
##    Data: dt.2
##
## REML criterion at convergence: 772.7
##
## Scaled residuals:
##     Min      1Q  Median      3Q     Max
## -3.8834 -0.5599  0.0354  0.6937  2.7326
##
## Random effects:
##  Groups        Name        Variance Std.Dev.
##  pop.block.eff (Intercept) 0.004536 0.06735
##  Residual                  0.492194 0.70157
## Number of obs: 358, groups:  pop.block.eff, 6
```

```
##
## Fixed effects:
##                  Estimate Std. Error        df t value Pr(>|t|)
## (Intercept)       2.14644    0.56520 307.15221   3.798 0.000176 ***
## femsize           0.40534    0.10446 340.57436   3.880 0.000125 ***
## log(male.lytic)   0.27377    0.05549 322.37452   4.934 1.29e-06 ***
## ---
## Signif. codes:  0 '***' 0.001 '**' 0.01 '*' 0.05 '.' 0.1 ' ' 1
##
## Correlation of Fixed Effects:
##             (Intr) femsiz
## femsize     -0.843
## lg(ml.lytc) -0.539  0.009
```

```
anova(femloglys.3,femloglys.4)
```

```
## Data: dt.2
## Models:
## femloglys.4: log(fem.lytic) ~ femsize + log(male.lytic) + (1 | pop.block.e
ff)
## femloglys.3: log(fem.lytic) ~ population + femsize + log(male.lytic) + (1
| pop.block.eff)
##             npar    AIC    BIC  logLik deviance  Chisq Df Pr(>Chisq)
## femloglys.4    5 771.69 791.09 -380.84   761.69
## femloglys.3    7 775.28 802.44 -380.64   761.28 0.4106  2     0.8144
```

```
# femloglys.4 is minimal adequate model.
# We now check wether the model changes quantitatively without the random eff
ect population-per-block.
femloglys.4.lm <- lm(log(fem.lytic) ~ femsize + log(male.lytic) , data = dt.2
)
```

Validating the model:

```
validatemodel(femloglys.4, ident='femloglys4')
```

Lytic activity males

We already know that lytic activity values need log-transformation, hence we fit a GLMM on log-transformed values.

```
maleloglys.1 <- lmer(log(male.lytic) ~ population * malesize + log(fem.lytic)
+ carc.wt +   (1|pop.block.eff) + (1| male.fam), data = dt.2)
summary(maleloglys.1)
```

```
## Linear mixed model fit by REML. t-tests use Satterthwaite's method [
## lmerModLmerTest]
## Formula: log(male.lytic) ~ population * malesize + log(fem.lytic) + carc.w
t +
##     (1 | pop.block.eff) + (1 | male.fam)
##    Data: dt.2
##
```

```
## REML criterion at convergence: 718.1
##
## Scaled residuals:
##     Min      1Q  Median      3Q     Max
## -5.3589 -0.5412  0.0232  0.6163  2.2573
##
## Random effects:
##  Groups        Name        Variance Std.Dev.
##  male.fam      (Intercept) 0.04407  0.2099
##  pop.block.eff (Intercept) 0.03197  0.1788
##  Residual                  0.36501  0.6042
## Number of obs: 360, groups:  male.fam, 140; pop.block.eff, 6
##
## Fixed effects:
##                           Estimate Std. Error        df t value Pr(>|t|)
## (Intercept)                2.12796    0.98855 334.32937   2.153   0.0321 *
## populationNC               0.67405    0.99481 286.55853   0.678   0.4986
## populationNm               0.81335    1.01184 290.79916   0.804   0.4221
## malesize                   0.29412    0.15304 348.97855   1.922   0.0554 .
## log(fem.lytic)             0.20450    0.04638 351.46158   4.409 1.38e-05 ***
## carc.wt                    0.06819    0.04722 339.34107   1.444   0.1497
## populationNC:malesize     -0.14179    0.21631 342.05062  -0.655   0.5126
## populationNm:malesize     -0.20981    0.21551 336.92165  -0.974   0.3310
## ---
## Signif. codes:  0 '***' 0.001 '**' 0.01 '*' 0.05 '.' 0.1 ' ' 1
##
## Correlation of Fixed Effects:
##             (Intr) ppltNC ppltnN malesz lg(f.) crc.wt pplNC:
## populatinNC -0.563
## populatinNm -0.518  0.524
## malesize    -0.726  0.718  0.703
## lg(fm.lytc) -0.308  0.087  0.027  0.040
## carc.wt     -0.616  0.006 -0.025 -0.007  0.033
## ppltnNC:mls  0.536 -0.979 -0.499 -0.710 -0.085 -0.005
## ppltnNm:mls  0.505 -0.509 -0.980 -0.710 -0.027  0.021  0.504
```

```
maleloglys.2 <- lmer(log(male.lytic) ~ population + malesize + log(fem.lytic)
+ carc.wt +   (1|pop.block.eff) + (1| male.fam), data = dt.2)
summary(maleloglys.2)
```

```
## Linear mixed model fit by REML. t-tests use Satterthwaite's method [
## lmerModLmerTest]
## Formula: log(male.lytic) ~ population + malesize + log(fem.lytic) + carc.w
t +
##     (1 | pop.block.eff) + (1 | male.fam)
##    Data: dt.2
##
## REML criterion at convergence: 716.3
##
## Scaled residuals:
```

```
##     Min      1Q  Median      3Q     Max 
## -5.3999 -0.5923  0.0117  0.6221  2.2719 
## 
## Random effects:
##  Groups        Name        Variance Std.Dev.
##  male.fam      (Intercept) 0.04506  0.2123  
##  pop.block.eff (Intercept) 0.03092  0.1758  
##  Residual                  0.36317  0.6026  
## Number of obs: 360, groups:  male.fam, 140; pop.block.eff, 6
## 
## Fixed effects:
##                  Estimate Std. Error        df t value Pr(>|t|)    
## (Intercept)       2.67556    0.78856 325.23925   3.393 0.000777 ***
## populationNC      0.02206    0.20036   3.01170   0.110 0.919250    
## populationNm     -0.15330    0.19747   2.84197  -0.776 0.496999    
## malesize          0.17644    0.08778 336.05589   2.010 0.045217 *  
## log(fem.lytic)    0.20275    0.04614 353.27830   4.395 1.47e-05 ***
## carc.wt           0.06891    0.04713 341.27669   1.462 0.144612    
## ---
## Signif. codes:  0 '***' 0.001 '**' 0.01 '*' 0.05 '.' 0.1 ' ' 1
## 
## Correlation of Fixed Effects:
##             (Intr) ppltNC ppltnN malesz lg(f.)
## populatinNC -0.204                            
## populatinNm -0.130  0.500                     
## malesize    -0.511  0.131  0.043              
## lg(fm.lytc) -0.336  0.021  0.004 -0.022       
## carc.wt     -0.777  0.008 -0.024  0.001  0.032
```

```
anova(maleloglys.1,maleloglys.2)
```

```
## Data: dt.2
## Models:
## maleloglys.2: log(male.lytic) ~ population + malesize + log(fem.lytic) + c
arc.wt + (1 | pop.block.eff) + (1 | male.fam)
## maleloglys.1: log(male.lytic) ~ population * malesize + log(fem.lytic) + c
arc.wt + (1 | pop.block.eff) + (1 | male.fam)
##              npar    AIC    BIC  logLik deviance  Chisq Df Pr(>Chisq)
## maleloglys.2    9 715.14 750.11 -348.57   697.14                     
## maleloglys.1   11 718.21 760.95 -348.10   696.21 0.9319  2     0.6275
```

```
maleloglys.3 <- lmer(log(male.lytic) ~ population + malesize + log(fem.lytic)
+   (1|pop.block.eff) + (1| male.fam), data = dt.2)
summary(maleloglys.3)
```

```
## Linear mixed model fit by REML. t-tests use Satterthwaite's method [
## lmerModLmerTest]
## Formula: log(male.lytic) ~ population + malesize + log(fem.lytic) + (1 |  
##     pop.block.eff) + (1 | male.fam)
##    Data: dt.2
## 
```

```
## REML criterion at convergence: 714.2
##
## Scaled residuals:
##     Min      1Q  Median      3Q     Max
## -5.5193 -0.5877  0.0177  0.5972  2.3373
##
## Random effects:
##  Groups        Name        Variance Std.Dev.
##  male.fam      (Intercept) 0.04564  0.2136
##  pop.block.eff (Intercept) 0.02855  0.1690
##  Residual                  0.36420  0.6035
## Number of obs: 360, groups:  male.fam, 140; pop.block.eff, 6
##
## Fixed effects:
##                   Estimate Std. Error        df t value Pr(>|t|)
## (Intercept)        3.56815    0.49583 204.28972   7.196 1.16e-11 ***
## populationNC       0.02006    0.19448   3.01819   0.103   0.9243
## populationNm      -0.14627    0.19143   2.83398  -0.764   0.5034
## malesize           0.17660    0.08794 337.38731   2.008   0.0454 *
## log(fem.lytic)     0.20098    0.04619 354.37826   4.351 1.77e-05 ***
## ---
## Signif. codes:  0 '***' 0.001 '**' 0.01 '*' 0.05 '.' 0.1 ' ' 1
##
## Correlation of Fixed Effects:
##              (Intr) ppltNC ppltnN malesz
## populatinNC -0.313
## populatinNm -0.233  0.500
## malesize    -0.813  0.136  0.044
## lg(fm.lytc) -0.496  0.021  0.005 -0.022
```

```
anova(maleloglys.2,maleloglys.3)
```

```
## Data: dt.2
## Models:
## maleloglys.3: log(male.lytic) ~ population + malesize + log(fem.lytic) + (
1 | pop.block.eff) + (1 | male.fam)
## maleloglys.2: log(male.lytic) ~ population + malesize + log(fem.lytic) + c
arc.wt + (1 | pop.block.eff) + (1 | male.fam)
##              npar    AIC    BIC  logLik deviance  Chisq Df Pr(>Chisq)
## maleloglys.3    8 715.10 746.19 -349.55   699.10
## maleloglys.2    9 715.14 750.11 -348.57   697.14 1.9598  1     0.1615
```

```
maleloglys.4 <- lmer(log(male.lytic) ~ malesize + log(fem.lytic) +  (1| pop.b
lock.eff )+ (1| male.fam), data = dt.2)
summary(maleloglys.4)
```

```
## Linear mixed model fit by REML. t-tests use Satterthwaite's method [
## lmerModLmerTest]
## Formula: log(male.lytic) ~ malesize + log(fem.lytic) + (1 | pop.block.eff)
+
##     (1 | male.fam)
```

```
##    Data: dt.2
##
## REML criterion at convergence: 711.7
##
## Scaled residuals:
##     Min      1Q  Median      3Q     Max
## -5.5491 -0.5877  0.0319  0.6007  2.3482
##
## Random effects:
##  Groups        Name        Variance Std.Dev.
##  male.fam      (Intercept) 0.04597  0.2144
##  pop.block.eff (Intercept) 0.02056  0.1434
##  Residual                  0.36391  0.6033
## Number of obs: 360, groups:  male.fam, 140; pop.block.eff, 6
##
## Fixed effects:
##                   Estimate Std. Error        df t value Pr(>|t|)
## (Intercept)        3.53367    0.46584 312.07926   7.586 3.84e-13 ***
## malesize           0.17330    0.08679 333.86713   1.997   0.0467 *
## log(fem.lytic)     0.20219    0.04612 355.89976   4.384 1.54e-05 ***
## ---
## Signif. codes:  0 '***' 0.001 '**' 0.01 '*' 0.05 '.' 0.1 ' ' 1
##
## Correlation of Fixed Effects:
##              (Intr) malesz
## malesize     -0.827
## lg(fm.lytc) -0.519 -0.027
```

```
anova(maleloglys.3,maleloglys.4)
```

```
## Data: dt.2
## Models:
## maleloglys.4: log(male.lytic) ~ malesize + log(fem.lytic) + (1 | pop.block
.eff) + (1 | male.fam)
## maleloglys.3: log(male.lytic) ~ population + malesize + log(fem.lytic) + (
1 | pop.block.eff) + (1 | male.fam)
##              npar    AIC    BIC  logLik deviance  Chisq Df Pr(>Chisq)
## maleloglys.4    6 712.75 736.06 -350.37   700.75
## maleloglys.3    8 715.10 746.19 -349.55   699.10 1.6491  2     0.4384
```

```
# do the results change quantitatively if random effect removed?
malelys4.lm <- lm(log(male.lytic) ~ malesize + log(fem.lytic) , data = dt.2)
```

Validate the model:

```
validatemodel(maleloglys.4, ident='maleloglys.4')
```

Does male and female lytic activity differ? Result presented in main text.

```
df <- data.frame(population = dt.2$population, block=dt.2$block, pair=dt.2$pa
ir,carc.wt= dt.2$carc.wt,f.lys =  dt.2$fem.lytic, m.lys = dt.2$male.lytic)
```

```
library('reshape')

str(df)

## 'data.frame':    362 obs. of  6 variables:
##  $ population: chr  "FC" "FC" "FC" "FC" ...
##  $ block     : num  -0.5 -0.5 -0.5 -0.5 -0.5 -0.5 -0.5 -0.5 -0.5 -0.5 ...
##  $ pair      : int  1 2 3 5 6 9 10 11 12 13 ...
##  $ carc.wt   : num  12.1 12 13.1 13.8 13.2 ...
##  $ f.lys     : num  208.8 357.6 81.1 452.6 185.7 ...
##  $ m.lys     : num  470.4 31.5 222.7 171.7 251.9 ...

df.melted <- melt(df, id=c('population','block','pair', 'carc.wt') )
names(df.melted)[5:6] <- c('sex','lys.avg')

anova.lys.sex2 <- aov(log(lys.avg) ~ sex, data = df.melted)
summary(anova.lys.sex2)

##              Df Sum Sq Mean Sq F value Pr(>F)
## sex           1    0.6  0.6261   1.245  0.265
## Residuals   722  363.1  0.5029
```

Fitness consequences for broods from the changes in the nursery environment

Clutch size

Do parental regimes differ in the clutch size? Resuls presented in main text, Table S1 and Fig S2.

```
egg.1 <- lmer(eggnr ~ population * scale(femsize) + scale(malesize) + carc.wt
+ (1 | pop.block.eff) + (1|female.fam) + (1|male.fam), data =dt)
summary(egg.1)

## Linear mixed model fit by REML. t-tests use Satterthwaite's method [
## lmerModLmerTest]
## Formula: eggnr ~ population * scale(femsize) + scale(malesize) + carc.wt +
##     (1 | pop.block.eff) + (1 | female.fam) + (1 | male.fam)
##    Data: dt
##
## REML criterion at convergence: 2848.6
##
## Scaled residuals:
##      Min       1Q   Median       3Q      Max
## -2.37943 -0.65904 -0.05264  0.69718  2.95437
##
## Random effects:
##  Groups        Name        Variance Std.Dev.
##  male.fam      (Intercept)  0.0000  0.0000
##  female.fam    (Intercept)  0.0000  0.0000
##  pop.block.eff (Intercept)  0.1066  0.3265
##  Residual                  48.2921  6.9493
```

```
## Number of obs: 426, groups:  male.fam, 141; female.fam, 140; pop.block.eff
, 6
##
## Fixed effects:
##                             Estimate Std. Error        df t value Pr(>|t|
)
## (Intercept)                 18.72625    4.99658 407.08917   3.748 0.00020
4 ***
## populationNC                -4.27762    0.96772   4.00869  -4.420 0.01145
2 *
## populationNm                -3.99652    0.90745   3.12380  -4.404 0.01994
7 *
## scale(femsize)               3.10406    0.61394 368.13428   5.056 6.77e-0
7 ***
## scale(malesize)             -0.49273    0.35664 398.26423  -1.382 0.16787
0
## carc.wt                      0.03246    0.38457 417.73232   0.084 0.93277
5
## populationNC:scale(femsize)  -1.27578    0.88461 406.04402  -1.442 0.15001
9
## populationNm:scale(femsize)  -0.21035    0.84271 408.30647  -0.250 0.80300
9
## ---
## Signif. codes:  0 '***' 0.001 '**' 0.01 '*' 0.05 '.' 0.1 ' ' 1
##
## Correlation of Fixed Effects:
##             (Intr) ppltNC ppltnN scl(f) scl(m) crc.wt pNC:()
## populatinNC -0.146
## populatinNm -0.074  0.505
## scale(fmsz) -0.081  0.230  0.215
## scale(mlsz) -0.074  0.261  0.079  0.137
## carc.wt     -0.991  0.052 -0.022  0.041  0.050
## ppltnNC:s()  0.058 -0.013 -0.153 -0.700 -0.141 -0.029
## ppltnNm:s()  0.045 -0.159 -0.168 -0.724 -0.070 -0.016  0.506
## optimizer (nloptwrap) convergence code: 0 (OK)
## boundary (singular) fit: see ?isSingular
```

```
# male and female family do not explain any variance, so we remove those rand
om effects
egg.2 <- lmer(eggnr ~ population * scale(femsize) + scale(malesize) + carc.wt
+ (1 | pop.block.eff) , data =dt)
summary(egg.2)
```

```
## Linear mixed model fit by REML. t-tests use Satterthwaite's method [
## lmerModLmerTest]
## Formula: eggnr ~ population * scale(femsize) + scale(malesize) + carc.wt +
##     (1 | pop.block.eff)
##    Data: dt
##
## REML criterion at convergence: 2848.6
```

```
##
## Scaled residuals:
##      Min       1Q   Median       3Q      Max
## -2.37942 -0.65903 -0.05262  0.69718  2.95438
##
## Random effects:
##  Groups        Name        Variance Std.Dev.
##  pop.block.eff (Intercept)  0.1067  0.3267
##  Residual                  48.2921  6.9493
## Number of obs: 426, groups:  pop.block.eff, 6
##
## Fixed effects:
##                               Estimate Std. Error        df t value Pr(>|t|
)
## (Intercept)                   18.72620    4.99658 407.08830   3.748 0.00020
4 ***
## populationNC                  -4.27761    0.96776   4.00934  -4.420 0.01145
0 *
## populationNm                  -3.99652    0.90750   3.12438  -4.404 0.01994
2 *
## scale(femsize)                 3.10408    0.61395 368.15193   5.056 6.77e-0
7 ***
## scale(malesize)               -0.49272    0.35664 398.27172  -1.382 0.16787
8
## carc.wt                        0.03246    0.38457 417.73242   0.084 0.93276
9
## populationNC:scale(femsize)  -1.27580    0.88461 406.04864  -1.442 0.15001
5
## populationNm:scale(femsize)  -0.21036    0.84271 408.31025  -0.250 0.80300
2
## ---
## Signif. codes:  0 '***' 0.001 '**' 0.01 '*' 0.05 '.' 0.1 ' ' 1
##
## Correlation of Fixed Effects:
##             (Intr) ppltNC ppltnN scl(f) scl(m) crc.wt pNC:()
## populatinNC -0.146
## populatinNm -0.074  0.505
## scale(fmsz) -0.081  0.230  0.215
## scale(mlsz) -0.074  0.261  0.079  0.137
## carc.wt     -0.991  0.052 -0.022  0.041  0.050
## ppltnNC:s()  0.058 -0.013 -0.153 -0.700 -0.141 -0.029
## ppltnNm:s()  0.045 -0.159 -0.168 -0.724 -0.070 -0.016  0.506
```

```
anova(egg.1, egg.2)
```

```
## Data: dt
## Models:
## egg.2: eggnr ~ population * scale(femsize) + scale(malesize) + carc.wt + (
1 | pop.block.eff)
## egg.1: eggnr ~ population * scale(femsize) + scale(malesize) + carc.wt + (
```

```
1 | pop.block.eff) + (1 | female.fam) + (1 | male.fam)
##        npar  AIC    BIC  logLik deviance Chisq Df Pr(>Chisq)
## egg.2    10 2873 2913.6 -1426.5     2853
## egg.1    12 2877 2925.7 -1426.5     2853     0  2          1
```

```
egg.3 <- lmer(eggnr ~ population + scale(femsize) + scale(malesize) + carc.wt
+ (1|pop.block.eff), data =dt)
summary(egg.3)
```

```
## Linear mixed model fit by REML. t-tests use Satterthwaite's method [
## lmerModLmerTest]
## Formula: eggnr ~ population + scale(femsize) + scale(malesize) + carc.wt +
##     (1 | pop.block.eff)
##    Data: dt
##
## REML criterion at convergence: 2853.8
##
## Scaled residuals:
##     Min      1Q  Median      3Q     Max
## -2.3748 -0.7073 -0.0497  0.7407  2.9455
##
## Random effects:
##  Groups        Name        Variance Std.Dev.
##  pop.block.eff (Intercept)  0.00    0.000
##  Residual                  48.38    6.956
## Number of obs: 426, groups:  pop.block.eff, 6
##
## Fixed effects:
##                  Estimate Std. Error        df t value Pr(>|t|)
## (Intercept)      19.15492    4.98463 420.00000   3.843  0.00014 ***
## populationNC     -4.21170    0.89524 420.00000  -4.705 3.46e-06 ***
## populationNm     -4.14589    0.83052 420.00000  -4.992 8.78e-07 ***
## scale(femsize)    2.62251    0.35035 420.00000   7.485 4.23e-13 ***
## scale(malesize)  -0.57515    0.35283 420.00000  -1.630  0.10383
## carc.wt           0.01239    0.38461 420.00000   0.032  0.97431
## ---
## Signif. codes:  0 '***' 0.001 '**' 0.01 '*' 0.05 '.' 0.1 ' ' 1
##
## Correlation of Fixed Effects:
##             (Intr) ppltNC ppltnN scl(f) scl(m)
## populatinNC -0.142
## populatinNm -0.056  0.502
## scale(fmsz) -0.057  0.274  0.121
## scale(mlsz) -0.067  0.283  0.063  0.066
## carc.wt     -0.993  0.056 -0.030  0.034  0.047
## optimizer (nloptwrap) convergence code: 0 (OK)
## boundary (singular) fit: see ?isSingular
```

```
anova(egg.2, egg.3)
```

```
## Data: dt
## Models:
## egg.3: eggnr ~ population + scale(femsize) + scale(malesize) + carc.wt + (
1 | pop.block.eff)
## egg.2: eggnr ~ population * scale(femsize) + scale(malesize) + carc.wt + (
1 | pop.block.eff)
##       npar    AIC    BIC  logLik deviance  Chisq Df Pr(>Chisq)
## egg.3    8 2871.4 2903.8 -1427.7   2855.4
## egg.2   10 2873.0 2913.6 -1426.5   2853.0 2.3644  2     0.3066
```

```
egg.4 <- lmer(eggnr ~ population + scale(femsize) + scale(malesize) + (1|pop.
block.eff), data =dt)
summary(egg.4)
```

```
## Linear mixed model fit by REML. t-tests use Satterthwaite's method [
## lmerModLmerTest]
## Formula: eggnr ~ population + scale(femsize) + scale(malesize) + (1 |
##     pop.block.eff)
##    Data: dt
##
## REML criterion at convergence: 2853.7
##
## Scaled residuals:
##      Min       1Q   Median       3Q      Max
## -2.37646 -0.70739 -0.04858  0.74244  2.94974
##
## Random effects:
##  Groups        Name        Variance Std.Dev.
##  pop.block.eff (Intercept)  0.00    0.000
##  Residual                  48.27    6.948
## Number of obs: 426, groups:  pop.block.eff, 6
##
## Fixed effects:
##                  Estimate Std. Error       df t value Pr(>|t|)
## (Intercept)       19.3143     0.5997 421.0000  32.207  < 2e-16 ***
## populationNC      -4.2133     0.8928 421.0000  -4.719 3.23e-06 ***
## populationNm      -4.1451     0.8292 421.0000  -4.999 8.47e-07 ***
## scale(femsize)     2.6221     0.3497 421.0000   7.498 3.88e-13 ***
## scale(malesize)   -0.5757     0.3520 421.0000  -1.635    0.103
## ---
## Signif. codes:  0 '***' 0.001 '**' 0.01 '*' 0.05 '.' 0.1 ' ' 1
##
## Correlation of Fixed Effects:
##             (Intr) ppltNC ppltnN scl(f)
## populatinNC -0.724
## populatinNm -0.711  0.504
## scale(fmsz) -0.191  0.273  0.122
## scale(mlsz) -0.170  0.281  0.064  0.065
## optimizer (nloptwrap) convergence code: 0 (OK)
## boundary (singular) fit: see ?isSingular
```

```r
# We will go with egg.4 as minimal adequate model
# carcass mass is not significant and removing decreases AIC by 2 units
anova(egg.3, egg.4)

## Data: dt
## Models:
## egg.4: eggnr ~ population + scale(femsize) + scale(malesize) + (1 | pop.bl
ock.eff)
## egg.3: eggnr ~ population + scale(femsize) + scale(malesize) + carc.wt + (
1 | pop.block.eff)
##       npar    AIC    BIC  logLik deviance  Chisq Df Pr(>Chisq)
## egg.4    7 2869.4 2897.8 -1427.7   2855.4
## egg.3    8 2871.4 2903.8 -1427.7   2855.4 0.0011  1     0.9741

# We can now validate the model.
validatemodel(egg.4, ident='egg4')
# Checking if the results change quantitatively if random effect is removed.
egg4.lm <- lm(eggnr ~ population + scale(femsize) + scale(malesize) + carc.wt
, data =dt)
```

Breeding success

Fitting a binomial GLMM to beging with. Results presented in Table 2.

```r
m.success1 <- glmer(success ~ population * donor.population * donor.hole + (1
|pop.block.eff), family='binomial', data =dt)
summary(m.success1)

## Generalized linear mixed model fit by maximum likelihood (Laplace
##   Approximation) [glmerMod]
##  Family: binomial  ( logit )
## Formula:
## success ~ population * donor.population * donor.hole + (1 | pop.block.eff)
##    Data: dt
##
##      AIC      BIC   logLik deviance df.resid
##    312.0    389.3   -137.0    274.0      414
##
## Scaled residuals:
##     Min      1Q  Median      3Q     Max
## -5.0990  0.0001  0.2673  0.4851  0.7338
##
## Random effects:
##  Groups        Name        Variance Std.Dev.
##  pop.block.eff (Intercept) 0        0
## Number of obs: 433, groups:  pop.block.eff, 6
##
## Fixed effects:
##                                                 Estimate Std. Error z value
## (Intercept)                                    1.061e+00  3.867e-01   2.743
## populationNC                                   2.121e-01  5.766e-01   0.368
```

```
## populationNm                                   1.306e+00  7.170e-01   1.822
## donor.populationNC                             3.860e-01  6.770e-01   0.570
## donor.populationNm                             2.609e-01  6.828e-01   0.382
## donor.hole                                     1.850e+01  4.557e+03   0.004
## populationNC:donor.populationNC               -7.427e-01  9.956e-01  -0.746
## populationNm:donor.populationNC               -1.500e+00  1.070e+00  -1.403
## populationNC:donor.populationNm               -9.148e-01  9.321e-01  -0.981
## populationNm:donor.populationNm               -8.934e-01  1.106e+00  -0.808
## populationNC:donor.hole                       -1.713e+01  4.557e+03  -0.004
## populationNm:donor.hole                       -1.830e+01  4.557e+03  -0.004
## donor.populationNC:donor.hole                 -1.672e+01  4.557e+03  -0.004
## donor.populationNm:donor.hole                 -2.590e-01  5.583e+03   0.000
## populationNC:donor.populationNC:donor.hole     1.721e+01  4.557e+03   0.004
## populationNm:donor.populationNC:donor.hole     5.261e+01  1.246e+07   0.000
## populationNC:donor.populationNm:donor.hole     1.532e+00  5.583e+03   0.000
## populationNm:donor.populationNm:donor.hole     9.293e-01  5.583e+03   0.000
##                                               Pr(>|z|)
## (Intercept)                                    0.00609 **
## populationNC                                   0.71298
## populationNm                                   0.06850 .
## donor.populationNC                             0.56855
## donor.populationNm                             0.70241
## donor.hole                                     0.99676
## populationNC:donor.populationNC                0.45567
## populationNm:donor.populationNC                0.16075
## populationNC:donor.populationNm                0.32639
## populationNm:donor.populationNm                0.41917
## populationNC:donor.hole                        0.99700
## populationNm:donor.hole                        0.99680
## donor.populationNC:donor.hole                  0.99707
## donor.populationNm:donor.hole                  0.99996
## populationNC:donor.populationNC:donor.hole     0.99699
## populationNm:donor.populationNC:donor.hole     1.00000
## populationNC:donor.populationNm:donor.hole     0.99978
## populationNm:donor.populationNm:donor.hole     0.99987
## ---
## Signif. codes:  0 '***' 0.001 '**' 0.01 '*' 0.05 '.' 0.1 ' ' 1
## optimizer (Nelder_Mead) convergence code: 0 (OK)
## boundary (singular) fit: see ?isSingular

# random effect explains no variance, hence we remove it

m.success2 <- glm(success ~ population * donor.population * donor.hole - popu
lation:donor.population:donor.hole , family='binomial', data =dt)
summary(m.success2)

##
## Call:
## glm(formula = success ~ population * donor.population * donor.hole -
##     population:donor.population:donor.hole, family = "binomial",
```

```
##     data = dt)
##
## Deviance Residuals:
##     Min       1Q   Median       3Q      Max
## -2.9935   0.2364   0.3129   0.6405   0.8936
##
## Coefficients:
##                                    Estimate Std. Error z value Pr(>|z|)
## (Intercept)                          1.1236     0.3874   2.901  0.00372 **
## populationNC                         0.1420     0.5626   0.252  0.80079
## populationNm                         1.1146     0.6657   1.674  0.09408 .
## donor.populationNC                   0.1185     0.6358   0.186  0.85220
## donor.populationNm                   0.2933     0.6766   0.433  0.66470
## donor.hole                           2.4402     1.1787   2.070  0.03844 *
## populationNC:donor.populationNC     -0.5959     0.9062  -0.658  0.51080
## populationNm:donor.populationNC     -0.7143     0.9986  -0.715  0.47442
## populationNC:donor.populationNm     -0.8470     0.8930  -0.948  0.34288
## populationNm:donor.populationNm     -1.0515     0.9950  -1.057  0.29059
## populationNC:donor.hole             -1.0227     1.2111  -0.844  0.39843
## populationNm:donor.hole             -1.6186     1.2641  -1.280  0.20042
## donor.populationNC:donor.hole        0.7869     1.0548   0.746  0.45565
## donor.populationNm:donor.hole        0.7644     1.0413   0.734  0.46288
## ---
## Signif. codes:  0 '***' 0.001 '**' 0.01 '*' 0.05 '.' 0.1 ' ' 1
##
## (Dispersion parameter for binomial family taken to be 1)
##
##     Null deviance: 321.88  on 432  degrees of freedom
## Residual deviance: 280.39  on 419  degrees of freedom
## AIC: 308.39
##
## Number of Fisher Scoring iterations: 6
```

```
m.success3 <- glm(success ~ population * donor.population + donor.hole , fami
ly='binomial', data =dt)
summary(m.success3)
```

```
##
## Call:
## glm(formula = success ~ population * donor.population + donor.hole,
##     family = "binomial", data = dt)
##
## Deviance Residuals:
##     Min       1Q   Median       3Q      Max
## -2.8898   0.2348   0.3285   0.6269   0.8819
##
## Coefficients:
##                                    Estimate Std. Error z value Pr(>|z|)
## (Intercept)                         1.15066    0.38066   3.023   0.0025 **
## populationNC                        0.04407    0.55116   0.080   0.9363
```

```
## populationNm                      0.95924    0.65009   1.476   0.1401
## donor.populationNC                0.23821    0.62243   0.383   0.7019
## donor.populationNm                0.43516    0.66214   0.657   0.5111
## donor.hole                        2.05006    0.41159   4.981 6.33e-07 ***
## populationNC:donor.populationNC -0.59046    0.87518  -0.675   0.4999
## populationNm:donor.populationNC -0.82095    0.97897  -0.839   0.4017
## populationNC:donor.populationNm -0.88605    0.87563  -1.012   0.3116
## populationNm:donor.populationNm -1.18209    0.97754  -1.209   0.2266
## ---
## Signif. codes:  0 '***' 0.001 '**' 0.01 '*' 0.05 '.' 0.1 ' ' 1
##
## (Dispersion parameter for binomial family taken to be 1)
##
##     Null deviance: 321.88  on 432  degrees of freedom
## Residual deviance: 282.66  on 423  degrees of freedom
## AIC: 302.66
##
## Number of Fisher Scoring iterations: 6
```

```
anova(m.success2, m.success3, test="Chisq")
```

```
## Analysis of Deviance Table
##
## Model 1: success ~ population * donor.population * donor.hole - population
:donor.population:donor.hole
## Model 2: success ~ population * donor.population + donor.hole
##   Resid. Df Resid. Dev Df Deviance Pr(>Chi)
## 1       419     280.39
## 2       423     282.66 -4  -2.2694   0.6863
```

```
m.success4 <- glm(success ~ population + donor.population + donor.hole , fami
ly='binomial', data =dt)
summary(m.success4)
```

```
##
## Call:
## glm(formula = success ~ population + donor.population + donor.hole,
##     family = "binomial", data = dt)
##
## Deviance Residuals:
##     Min       1Q   Median       3Q      Max
## -2.7858   0.2290   0.3225   0.6104   0.8453
##
## Coefficients:
##                    Estimate Std. Error z value Pr(>|z|)
## (Intercept)          1.4564     0.3235   4.502 6.72e-06 ***
## populationNC        -0.3794     0.3585  -1.058    0.290
## populationNm         0.3611     0.3968   0.910    0.363
## donor.populationNC  -0.1884     0.3835  -0.491    0.623
## donor.populationNm  -0.2317     0.3702  -0.626    0.531
## donor.hole           2.0420     0.4095   4.986 6.17e-07 ***
```

```
## ---
## Signif. codes:  0 '***' 0.001 '**' 0.01 '*' 0.05 '.' 0.1 ' ' 1
##
## (Dispersion parameter for binomial family taken to be 1)
##
##     Null deviance: 321.88  on 432  degrees of freedom
## Residual deviance: 284.54  on 427  degrees of freedom
## AIC: 296.54
##
## Number of Fisher Scoring iterations: 6
```

```
anova(m.success3, m.success4, test="Chisq")
```

```
## Analysis of Deviance Table
##
## Model 1: success ~ population * donor.population + donor.hole
## Model 2: success ~ population + donor.population + donor.hole
##   Resid. Df Resid. Dev Df Deviance Pr(>Chi)
## 1       423     282.66
## 2       427     284.54 -4   -1.882   0.7575
```

```
m.success5 <- glm(success ~ population + donor.hole , family='binomial', data
=dt)
summary(m.success5)
```

```
##
## Call:
## glm(formula = success ~ population + donor.hole, family = "binomial",
##     data = dt)
##
## Deviance Residuals:
##     Min       1Q   Median       3Q      Max
## -2.7251   0.2223   0.3195   0.5784   0.8037
##
## Coefficients:
##              Estimate Std. Error z value Pr(>|z|)
## (Intercept)    1.3419     0.2687   4.994 5.93e-07 ***
## populationNC  -0.3777     0.3579  -1.055    0.291
## populationNm   0.3615     0.3964   0.912    0.362
## donor.hole     1.9850     0.3994   4.970 6.70e-07 ***
## ---
## Signif. codes:  0 '***' 0.001 '**' 0.01 '*' 0.05 '.' 0.1 ' ' 1
##
## (Dispersion parameter for binomial family taken to be 1)
##
##     Null deviance: 321.88  on 432  degrees of freedom
## Residual deviance: 284.99  on 429  degrees of freedom
## AIC: 292.99
##
## Number of Fisher Scoring iterations: 6
```

```
anova(m.success4, m.success5, test="Chisq")

## Analysis of Deviance Table
##
## Model 1: success ~ population + donor.population + donor.hole
## Model 2: success ~ population + donor.hole
##   Resid. Df Resid. Dev Df Deviance Pr(>Chi)
## 1       427     284.54
## 2       429     284.99 -2 -0.45034   0.7984

m.success6 <- glm(success ~ donor.hole.factor , family='binomial', data =dt)
anova(m.success5,m.success6)

## Analysis of Deviance Table
##
## Model 1: success ~ population + donor.hole
## Model 2: success ~ donor.hole.factor
##   Resid. Df Resid. Dev Df Deviance
## 1       429     284.99
## 2       431     288.79 -2  -3.7965

AIC(m.success5,m.success6)

##            df      AIC
## m.success5  4 292.9941
## m.success6  2 292.7906

# m.success5 is the minimal adequate model, AIC is not reduced further

# check that the results are not quantitatively affected by the presence/abse
nce of random effect
m.success5.glmer <- glmer(success ~ population + donor.hole + (1|pop.block.ef
f), family='binomial', data =dt)

validatemodel(m.success5, ident="success5")
```

Brood size

Results reported in Table 1 and Figures 2 and 3. Make sure we do not include any pairs where there were no eggs visible, and exclude failures from brood size analysis.

```
dt.3 <- dt[!is.na(dt$eggnr),]
dt.3.nofail <- dt.3[dt.3$broodsize > 0,]
#remove empty rows
dt.nofail<- dt.3.nofail[rowSums(is.na(dt.3.nofail)) != ncol(dt.3.nofail),]
```

We will start by fitting a GLMM and doing model selection as above.

```
brood.1 <- lmer(broodsize ~ population * donor.population + femsize + donor.h
ole.factor + population * donor.hole.factor + donor.population*donor.hole.fac
tor + donor.round.adj + (1|pop.block.eff), data = dt.nofail)
summary(brood.1)
```

```
## Linear mixed model fit by REML. t-tests use Satterthwaite's method [
## lmerModLmerTest]
## Formula:
## broodsize ~ population * donor.population + femsize + donor.hole.factor +
##     population * donor.hole.factor + donor.population * donor.hole.factor
+
##     donor.round.adj + (1 | pop.block.eff)
##    Data: dt.nofail
##
## REML criterion at convergence: 2679
##
## Scaled residuals:
##      Min       1Q   Median       3Q      Max
## -2.65685 -0.74560  0.00399  0.68371  2.45922
##
## Random effects:
##  Groups        Name        Variance Std.Dev.
##  pop.block.eff (Intercept)  0.3188  0.5646
##  Residual                  87.4970  9.3540
## Number of obs: 375, groups:  pop.block.eff, 6
##
## Fixed effects:
##                                          Estimate Std. Error      df t val
ue
## (Intercept)                               -11.938      7.928 355.673  -1.5
06
## populationNC                                0.651      2.403  31.897   0.2
71
## populationNm                                1.343      2.268  25.872   0.5
92
## donor.populationNC                         -7.738      2.435 356.356  -3.1
78
## donor.populationNm                         -9.991      2.482 358.897  -4.0
25
## femsize                                     5.560      1.464 358.741   3.7
99
## donor.hole.factorHole                      10.025      2.282 356.551   4.3
94
## donor.round.adj                             5.862      4.312 356.647   1.3
59
## populationNC:donor.populationNC             8.184      3.076 356.089   2.6
61
## populationNm:donor.populationNC             6.388      2.989 356.572   2.1
38
## populationNC:donor.populationNm             5.227      3.065 356.706   1.7
05
## populationNm:donor.populationNm             9.823      2.954 357.122   3.3
25
## populationNC:donor.hole.factorHole         -5.857      2.558 358.231  -2.2
89
```

```
## populationNm:donor.hole.factorHole         -7.587      2.460 357.334  -3.0
85
## donor.populationNC:donor.hole.factorHole    2.398      2.536 356.901   0.9
46
## donor.populationNm:donor.hole.factorHole    5.218      2.507 358.912   2.0
82
##                                          Pr(>|t|)
## (Intercept)                              0.133032
## populationNC                             0.788224
## populationNm                             0.558907
## donor.populationNC                       0.001613 **
## donor.populationNm                       6.95e-05 ***
## femsize                                  0.000171 ***
## donor.hole.factorHole                    1.47e-05 ***
## donor.round.adj                          0.174939
## populationNC:donor.populationNC          0.008147 **
## populationNm:donor.populationNC          0.033236 *
## populationNC:donor.populationNm          0.089001 .
## populationNm:donor.populationNm          0.000976 ***
## populationNC:donor.hole.factorHole       0.022650 *
## populationNm:donor.hole.factorHole       0.002196 **
## donor.populationNC:donor.hole.factorHole 0.345032
## donor.populationNm:donor.hole.factorHole 0.038078 *
## ---
## Signif. codes:  0 '***' 0.001 '**' 0.01 '*' 0.05 '.' 0.1 ' ' 1
```

```
# which interactions are significant?

brood.1.2 <- lmer(broodsize ~ population + donor.population + femsize + donor
.hole.factor + population * donor.hole.factor + donor.population*donor.hole.f
actor + donor.round.adj + (1|pop.block.eff), data = dt.nofail)
# population x donor.population significant
anova(brood.1,brood.1.2, test="F")
```

```
## Data: dt.nofail
## Models:
## brood.1.2: broodsize ~ population + donor.population + femsize + donor.hol
e.factor + population * donor.hole.factor + donor.population * donor.hole.fac
tor + donor.round.adj + (1 | pop.block.eff)
## brood.1: broodsize ~ population * donor.population + femsize + donor.hole.
factor + population * donor.hole.factor + donor.population * donor.hole.facto
r + donor.round.adj + (1 | pop.block.eff)
##           npar    AIC    BIC  logLik deviance  Chisq Df Pr(>Chisq)
## brood.1.2   14 2769.6 2824.6 -1370.8   2741.6
## brood.1     18 2761.4 2832.1 -1362.7   2725.4 16.195  4   0.002768 **
## ---
## Signif. codes:  0 '***' 0.001 '**' 0.01 '*' 0.05 '.' 0.1 ' ' 1
```

```
brood.1.3 <- lmer(broodsize ~ population * donor.population + femsize + donor
.hole.factor + population * donor.hole.factor +  donor.round.adj + (1|pop.blo
```

```
ck.eff), data = dt.nofail)
# donor.population x donor.hole NOT significant
anova(brood.1, brood.1.3, test="F")
```

```
## Data: dt.nofail
## Models:
## brood.1.3: broodsize ~ population * donor.population + femsize + donor.hol
e.factor + population * donor.hole.factor + donor.round.adj + (1 | pop.block.
eff)
## brood.1: broodsize ~ population * donor.population + femsize + donor.hole.
factor + population * donor.hole.factor + donor.population * donor.hole.facto
r + donor.round.adj + (1 | pop.block.eff)
##           npar    AIC    BIC  logLik deviance  Chisq Df Pr(>Chisq)
## brood.1.3   16 2761.9 2824.7 -1365.0   2729.9
## brood.1     18 2761.4 2832.1 -1362.7   2725.4 4.4976  2     0.1055
```

```
brood.1.4 <- lmer(broodsize ~ population * donor.population + femsize + donor
.hole.factor + donor.population*donor.hole.factor + donor.round.adj + (1|pop.
block.eff), data = dt.nofail)
# population x donor.hole significant
anova(brood.1, brood.1.4)
```

```
## Data: dt.nofail
## Models:
## brood.1.4: broodsize ~ population * donor.population + femsize + donor.hol
e.factor + donor.population * donor.hole.factor + donor.round.adj + (1 | pop.
block.eff)
## brood.1: broodsize ~ population * donor.population + femsize + donor.hole.
factor + population * donor.hole.factor + donor.population * donor.hole.facto
r + donor.round.adj + (1 | pop.block.eff)
##           npar    AIC    BIC  logLik deviance  Chisq Df Pr(>Chisq)
## brood.1.4   16 2768.0 2830.8 -1368.0   2736.0
## brood.1     18 2761.4 2832.1 -1362.7   2725.4 10.568  2   0.005073 **
## ---
## Signif. codes:  0 '***' 0.001 '**' 0.01 '*' 0.05 '.' 0.1 ' ' 1
```

```
brood.1.5 <- lmer(broodsize ~ population * donor.population + femsize + donor
.hole.factor + donor.round.adj + (1|pop.block.eff), data = dt.nofail)
# population x donor.hole is significant when compared to model m1.3, and m1.
3 has lower AIC
anova(brood.1.3, brood.1.5)
```

```
## Data: dt.nofail
## Models:
## brood.1.5: broodsize ~ population * donor.population + femsize + donor.hol
e.factor + donor.round.adj + (1 | pop.block.eff)
## brood.1.3: broodsize ~ population * donor.population + femsize + donor.hol
e.factor + population * donor.hole.factor + donor.round.adj + (1 | pop.block.
eff)
##           npar    AIC    BIC  logLik deviance  Chisq Df Pr(>Chisq)
## brood.1.5   14 2768.2 2823.2 -1370.1   2740.2
```

```
## brood.1.3   16 2761.9 2824.7 -1365.0   2729.9 10.299  2   0.005801 **
## ---
## Signif. codes:  0 '***' 0.001 '**' 0.01 '*' 0.05 '.' 0.1 ' ' 1
```

```
# test for effect of donor.round.adj
brood.1.6 <- lmer(broodsize ~ population * donor.population + femsize + donor
.hole.factor + population * donor.hole.factor + (1|pop.block.eff), data = dt.
nofail)
# donor.round.adj does not significantly improve fit
anova(brood.1.3, brood.1.6)
```

```
## Data: dt.nofail
## Models:
## brood.1.6: broodsize ~ population * donor.population + femsize + donor.hol
e.factor + population * donor.hole.factor + (1 | pop.block.eff)
## brood.1.3: broodsize ~ population * donor.population + femsize + donor.hol
e.factor + population * donor.hole.factor + donor.round.adj + (1 | pop.block.
eff)
##             npar    AIC    BIC logLik deviance Chisq Df Pr(>Chisq)
## brood.1.6   15 2762.1 2821.0  -1366   2732.1
## brood.1.3   16 2761.9 2824.7  -1365   2729.9 2.161  1     0.1416
```

```
# hence, brood.1.6 is minimal model

#inspect residuals
validatemodel(brood.1.5, ident="brood1.5")

# to obtain final p-values for interactions
brood.1.7 <- lmer(broodsize ~ population + donor.population + femsize + donor
.hole.factor + population * donor.hole.factor + (1|pop.block.eff), data = dt.
nofail)
anova(brood.1.6, brood.1.7)
```

```
## Data: dt.nofail
## Models:
## brood.1.7: broodsize ~ population + donor.population + femsize + donor.hol
e.factor + population * donor.hole.factor + (1 | pop.block.eff)
## brood.1.6: broodsize ~ population * donor.population + femsize + donor.hol
e.factor + population * donor.hole.factor + (1 | pop.block.eff)
##             npar    AIC    BIC  logLik deviance Chisq Df Pr(>Chisq)
## brood.1.7   11 2769.1 2812.3 -1373.5   2747.1
## brood.1.6   15 2762.1 2821.0 -1366.0   2732.1 15.03  4    0.00464 **
## ---
## Signif. codes:  0 '***' 0.001 '**' 0.01 '*' 0.05 '.' 0.1 ' ' 1
```

```
brood.1.8 <- lmer(broodsize ~ population * donor.population + femsize + donor
.hole.factor + population + donor.hole.factor + (1|pop.block.eff), data = dt.
nofail)
anova(brood.1.6, brood.1.8)
```

```
## Data: dt.nofail
## Models:
```

```
## brood.1.8: broodsize ~ population * donor.population + femsize + donor.hol
e.factor + population + donor.hole.factor + (1 | pop.block.eff)
## brood.1.6: broodsize ~ population * donor.population + femsize + donor.hol
e.factor + population * donor.hole.factor + (1 | pop.block.eff)
##           npar    AIC  BIC  logLik deviance  Chisq Df Pr(>Chisq)
## brood.1.8   13 2769.0 2820 -1371.5   2743.0
## brood.1.6   15 2762.1 2821 -1366.0   2732.1 10.902  2   0.004293 **
## ---
## Signif. codes:  0 '***' 0.001 '**' 0.01 '*' 0.05 '.' 0.1 ' ' 1

# checking that model is quantitatively the same when random effect not inclu
ded
brood.1.6.lm <- lm(broodsize ~ population * donor.population + femsize + dono
r.hole.factor + population * donor.hole.factor , data = dt.nofail)
```

Post-hoc comparisons with emmeans package. Results reported in Tables S2 and S3.

```
library(emmeans)
# Table S2
broodhoc.1 <- emmeans(brood.1.6, list(pairwise ~  population | donor.hole.fac
tor), adjust='tukey')
summary(broodhoc.1)

## $`emmeans of population | donor.hole.factor`
## donor.hole.factor = No hole:
##  population emmean   SE    df lower.CL upper.CL
##  FC           12.3 1.34 12.57      9.4     15.2
##  NC           17.5 1.49 18.68     14.4     20.7
##  Nm           19.1 1.30 11.00     16.2     21.9
##
## donor.hole.factor = Hole:
##  population emmean   SE    df lower.CL upper.CL
##  FC           24.8 1.24  9.39     22.0     27.6
##  NC           24.0 1.25  9.45     21.2     26.8
##  Nm           23.8 1.23  9.09     21.0     26.6
##
## Results are averaged over the levels of: donor.population
## Degrees-of-freedom method: satterthwaite
## Confidence level used: 0.95
##
## $`pairwise differences of population | donor.hole.factor`
## donor.hole.factor = No hole:
##  2       estimate   SE    df t.ratio p.value
##  FC - NC   -5.236 2.01 15.80  -2.601  0.0483
##  FC - Nm   -6.766 1.86 11.79  -3.630  0.0092
##  NC - Nm   -1.530 1.97 14.62  -0.776  0.7233
##
## donor.hole.factor = Hole:
##  2       estimate   SE    df t.ratio p.value
##  FC - NC    0.763 1.78  9.83   0.429  0.9046
##  FC - Nm    0.984 1.75  9.22   0.562  0.8432
```

```
##   NC - Nm    0.220 1.76  9.29   0.125  0.9914
##
## Results are averaged over the levels of: donor.population
## Degrees-of-freedom method: satterthwaite
## P value adjustment: tukey method for comparing a family of 3 estimates
```

```
broodhoc.2 <- emmeans(brood.1.6, list(pairwise ~ donor.population | populatio
n), adjust='tukey')
summary(broodhoc.2)
```

```
## $`emmeans of donor.population | population`
## population = FC:
##  donor.population emmean   SE   df lower.CL upper.CL
##  FC                 23.1 1.56 22.6     19.8     26.3
##  NC                 16.4 1.52 20.6     13.2     19.6
##  Nm                 16.2 1.54 21.7     13.0     19.4
##
## population = NC:
##  donor.population emmean   SE   df lower.CL upper.CL
##  FC                 20.9 1.61 25.4     17.6     24.2
##  NC                 22.2 1.60 25.3     18.9     25.5
##  Nm                 19.2 1.62 26.5     15.9     22.6
##
## population = Nm:
##  donor.population emmean   SE   df lower.CL upper.CL
##  FC                 20.6 1.51 19.9     17.4     23.7
##  NC                 20.5 1.52 20.7     17.3     23.6
##  Nm                 23.3 1.49 19.1     20.2     26.4
##
## Results are averaged over the levels of: donor.hole.factor
## Degrees-of-freedom method: satterthwaite
## Confidence level used: 0.95
##
## $`pairwise differences of donor.population | population`
## population = FC:
##  2        estimate   SE  df t.ratio p.value
##  FC - NC    6.6771 2.10 359   3.172  0.0047
##  FC - Nm    6.8961 2.12 361   3.254  0.0036
##  NC - Nm    0.2189 2.06 360   0.106  0.9938
##
## population = NC:
##  2        estimate   SE  df t.ratio p.value
##  FC - NC   -1.3277 2.24 359  -0.593  0.8238
##  FC - Nm    1.6632 2.23 359   0.745  0.7367
##  NC - Nm    2.9909 2.12 359   1.410  0.3367
##
## population = Nm:
##  2        estimate   SE  df t.ratio p.value
##  FC - NC    0.0911 2.12 360   0.043  0.9990
##  FC - Nm   -2.7113 2.07 359  -1.309  0.3911
```

```
##  NC - Nm  -2.8024 2.02 359  -1.387  0.3488
##
## Results are averaged over the levels of: donor.hole.factor
## Degrees-of-freedom method: satterthwaite
## P value adjustment: tukey method for comparing a family of 3 estimates
```

Brood mass

Results reported in Table 2 and Figures 2 and 3.

```
mass.1 <- lmer(broodmass ~ population * donor.population + femsize + donor.ho
le.factor + population * donor.hole.factor + donor.population*donor.hole.fact
or + donor.round.adj + (1|pop.block.eff), data = dt.nofail)
summary(mass.1)
```

```
## Linear mixed model fit by REML. t-tests use Satterthwaite's method [
## lmerModLmerTest]
## Formula:
## broodmass ~ population * donor.population + femsize + donor.hole.factor +
##     population * donor.hole.factor + donor.population * donor.hole.factor
+
##     donor.round.adj + (1 | pop.block.eff)
##    Data: dt.nofail
##
## REML criterion at convergence: 966.9
##
## Scaled residuals:
##     Min      1Q  Median      3Q     Max
## -3.1472 -0.5981  0.1564  0.7014  2.1385
##
## Random effects:
##  Groups        Name        Variance Std.Dev.
##  pop.block.eff (Intercept) 0.003505 0.0592
##  Residual                  0.742208 0.8615
## Number of obs: 375, groups:  pop.block.eff, 6
##
## Fixed effects:
##                                           Estimate Std. Error        df t
value
## (Intercept)                               -0.74760    0.73056 355.38956  -
1.023
## populationNC                               0.59101    0.22314  30.94539
2.649
## populationNm                               0.55494    0.21083  25.16101
2.632
## donor.populationNC                        -0.40813    0.22426 356.44663  -
1.820
## donor.populationNm                        -0.43444    0.22866 358.85133  -
1.900
## femsize                                    0.47789    0.13482 358.68893
3.545
```

```
## donor.hole.factorHole                      0.80499    0.21015 356.62343
3.831
## donor.round.adj                            0.39545    0.39719 356.71170
0.996
## populationNC:donor.populationNC            0.48629    0.28327 356.20353
1.717
## populationNm:donor.populationNC            0.49499    0.27526 356.64314
1.798
## populationNC:donor.populationNm            0.06246    0.28229 356.76821
0.221
## populationNm:donor.populationNm            0.63080    0.27212 357.14929
2.318
## populationNC:donor.hole.factorHole        -0.52399    0.23565 358.19307  -
2.224
## populationNm:donor.hole.factorHole        -0.86913    0.22655 357.34242  -
3.836
## donor.populationNC:donor.hole.factorHole   0.40331    0.23357 356.94448
1.727
## donor.populationNm:donor.hole.factorHole   0.52479    0.23092 358.86796
2.273
##                                          Pr(>|t|)
## (Intercept)                              0.306845
## populationNC                             0.012610 *
## populationNm                             0.014289 *
## donor.populationNC                       0.069615 .
## donor.populationNm                       0.058246 .
## femsize                                  0.000445 ***
## donor.hole.factorHole                    0.000151 ***
## donor.round.adj                          0.320106
## populationNC:donor.populationNC          0.086908 .
## populationNm:donor.populationNC          0.072982 .
## populationNC:donor.populationNm          0.825025
## populationNm:donor.populationNm          0.021007 *
## populationNC:donor.hole.factorHole       0.026802 *
## populationNm:donor.hole.factorHole       0.000148 ***
## donor.populationNC:donor.hole.factorHole 0.085083 .
## donor.populationNm:donor.hole.factorHole 0.023637 *
## ---
## Signif. codes:  0 '***' 0.001 '**' 0.01 '*' 0.05 '.' 0.1 ' ' 1
```

```
mass.1.1 <- lmer(broodmass ~ population * donor.population + femsize + donor.
hole.factor + population * donor.hole.factor + donor.round.adj + (1|pop.block
.eff), data = dt.nofail)
summary(mass.1.1)
```

```
## Linear mixed model fit by REML. t-tests use Satterthwaite's method [
## lmerModLmerTest]
## Formula:
## broodmass ~ population * donor.population + femsize + donor.hole.factor +
##     population * donor.hole.factor + donor.round.adj + (1 | pop.block.eff)
```

```
##    Data: dt.nofail
##
## REML criterion at convergence: 970
##
## Scaled residuals:
##     Min      1Q  Median      3Q     Max
## -3.1107 -0.5758  0.1705  0.6823  2.1671
##
## Random effects:
##  Groups        Name        Variance Std.Dev.
##  pop.block.eff (Intercept) 0.005299 0.07279
##  Residual                  0.748960 0.86542
## Number of obs: 375, groups:  pop.block.eff, 6
##
## Fixed effects:
##                                      Estimate Std. Error        df t value
## (Intercept)                          -0.96519    0.72752 355.46416  -1.327
## populationNC                          0.60380    0.22796  27.03214   2.649
## populationNm                          0.58131    0.21556  21.97844   2.697
## donor.populationNC                   -0.23931    0.19435 358.22248  -1.231
## donor.populationNm                   -0.18519    0.19750 359.36140  -0.938
## femsize                               0.49426    0.13510 360.57458   3.658
## donor.hole.factorHole                 1.11856    0.16041 358.77532   6.973
## donor.round.adj                       0.42866    0.39870 358.67933   1.075
## populationNC:donor.populationNC       0.50212    0.28368 358.19626   1.770
## populationNm:donor.populationNC       0.47365    0.27618 358.58585   1.715
## populationNC:donor.populationNm       0.07245    0.28354 358.75020   0.256
## populationNm:donor.populationNm       0.59759    0.27300 358.96778   2.189
## populationNC:donor.hole.factorHole   -0.53831    0.23661 360.04023  -2.275
## populationNm:donor.hole.factorHole   -0.86362    0.22750 359.20451  -3.796
##                                     Pr(>|t|)
## (Intercept)                         0.185466
## populationNC                        0.013325 *
## populationNm                        0.013180 *
## donor.populationNC                  0.219014
## donor.populationNm                  0.349057
## femsize                             0.000292 ***
## donor.hole.factorHole                1.5e-11 ***
## donor.round.adj                     0.283034
## populationNC:donor.populationNC     0.077572 .
## populationNm:donor.populationNC     0.087210 .
## populationNC:donor.populationNm     0.798454
## populationNm:donor.populationNm     0.029239 *
## populationNC:donor.hole.factorHole 0.023487 *
## populationNm:donor.hole.factorHole 0.000172 ***
## ---
## Signif. codes:  0 '***' 0.001 '**' 0.01 '*' 0.05 '.' 0.1 ' ' 1
```

```
# interaction between donor.population and hole is marginally significant, ma
ss1 has lower AIC by 2 units
anova(mass.1, mass.1.1)

## Data: dt.nofail
## Models:
## mass.1.1: broodmass ~ population * donor.population + femsize + donor.hole
.factor + population * donor.hole.factor + donor.round.adj + (1 | pop.block.e
ff)
## mass.1: broodmass ~ population * donor.population + femsize + donor.hole.f
actor + population * donor.hole.factor + donor.population * donor.hole.factor
+ donor.round.adj + (1 | pop.block.eff)
##          npar    AIC    BIC  logLik deviance  Chisq Df Pr(>Chisq)
## mass.1.1   16 974.91 1037.7 -471.46   942.91
## mass.1     18 972.98 1043.7 -468.49   936.98 5.9383  2    0.05135 .
## ---
## Signif. codes:  0 '***' 0.001 '**' 0.01 '*' 0.05 '.' 0.1 ' ' 1

mass.1.2 <- lmer(broodmass ~ population * donor.population + femsize + donor.
hole.factor + donor.population*donor.hole.factor  + donor.round.adj + (1|pop.
block.eff), data = dt.nofail)
summary(mass.1.2)

## Linear mixed model fit by REML. t-tests use Satterthwaite's method [
## lmerModLmerTest]
## Formula:
## broodmass ~ population * donor.population + femsize + donor.hole.factor +
##     donor.population * donor.hole.factor + donor.round.adj +
##     (1 | pop.block.eff)
##    Data: dt.nofail
##
## REML criterion at convergence: 979.1
##
## Scaled residuals:
##     Min      1Q  Median      3Q     Max
## -3.1898 -0.5975  0.2024  0.6712  2.0781
##
## Random effects:
##  Groups        Name        Variance Std.Dev.
##  pop.block.eff (Intercept) 0.001339 0.03659
##  Residual                  0.769687 0.87732
## Number of obs: 375, groups:  pop.block.eff, 6
##
## Fixed effects:
##                                           Estimate Std. Error        df t
value
## (Intercept)                               -0.56609    0.74140 358.46603  -
0.764
## populationNC                               0.39060    0.20471  28.26793
1.908
```

```
## populationNm                                0.26996    0.19527  23.60759
1.382
## donor.populationNC                         -0.31796    0.22693 358.57915  -
1.401
## donor.populationNm                         -0.30938    0.23002 360.93892  -
1.345
## femsize                                     0.45598    0.13691 360.88783
3.331
## donor.hole.factorHole                       0.34790    0.16845 359.46171
2.065
## donor.round.adj                             0.50935    0.40319 358.66823
1.263
## populationNC:donor.populationNC             0.36084    0.27742 358.59910
1.301
## populationNm:donor.populationNC             0.22392    0.27046 358.37176
0.828
## populationNC:donor.populationNm            -0.08079    0.27805 358.72745  -
0.291
## populationNm:donor.populationNm             0.37077    0.26821 358.95337
1.382
## donor.populationNC:donor.hole.factorHole    0.41807    0.23766 359.07043
1.759
## donor.populationNm:donor.hole.factorHole    0.51326    0.23488 360.99032
2.185
##                                           Pr(>|t|)
## (Intercept)                               0.445643
## populationNC                              0.066582 .
## populationNm                              0.179765
## donor.populationNC                        0.162037
## donor.populationNm                        0.179453
## femsize                                   0.000956 ***
## donor.hole.factorHole                     0.039618 *
## donor.round.adj                           0.207301
## populationNC:donor.populationNC           0.194195
## populationNm:donor.populationNC           0.408263
## populationNC:donor.populationNm           0.771561
## populationNm:donor.populationNm           0.167715
## donor.populationNC:donor.hole.factorHole 0.079408 .
## donor.populationNm:donor.hole.factorHole 0.029518 *
## ---
## Signif. codes:  0 '***' 0.001 '**' 0.01 '*' 0.05 '.' 0.1 ' ' 1
```

```
# interaction between population and hole is significant
anova(mass.1, mass.1.2)
```

```
## Data: dt.nofail
## Models:
## mass.1.2: broodmass ~ population * donor.population + femsize + donor.hole
.factor + donor.population * donor.hole.factor + donor.round.adj + (1 | pop.b
lock.eff)
```

```
## mass.1: broodmass ~ population * donor.population + femsize + donor.hole.f
actor + population * donor.hole.factor + donor.population * donor.hole.factor
+ donor.round.adj + (1 | pop.block.eff)
##          npar    AIC    BIC  logLik deviance  Chisq Df Pr(>Chisq)
## mass.1.2   16 984.11 1046.9 -476.05   952.11
## mass.1     18 972.98 1043.7 -468.49   936.98 15.133  2  0.0005176 ***
## ---
## Signif. codes:  0 '***' 0.001 '**' 0.01 '*' 0.05 '.' 0.1 ' ' 1
```

```
mass.1.3 <- lmer(broodmass ~ population + donor.population + femsize + donor.
hole.factor + population * donor.hole.factor + donor.population*donor.hole.fa
ctor + donor.round.adj + (1|pop.block.eff), data = dt.nofail)
summary(mass.1.3)
```

```
## Linear mixed model fit by REML. t-tests use Satterthwaite's method [
## lmerModLmerTest]
## Formula:
## broodmass ~ population + donor.population + femsize + donor.hole.factor +
##     population * donor.hole.factor + donor.population * donor.hole.factor
+
##     donor.round.adj + (1 | pop.block.eff)
##    Data: dt.nofail
##
## REML criterion at convergence: 973
##
## Scaled residuals:
##     Min      1Q  Median      3Q     Max
## -2.9597 -0.6320  0.1467  0.6962  2.3041
##
## Random effects:
##  Groups        Name        Variance Std.Dev.
##  pop.block.eff (Intercept) 0.003257 0.05707
##  Residual                  0.755153 0.86900
## Number of obs: 375, groups:  pop.block.eff, 6
##
## Fixed effects:
##                                            Estimate Std. Error        df t
value
## (Intercept)                                -0.99535    0.72691 358.81380  -
1.369
## populationNC                                0.74065    0.18363  14.66312
4.033
## populationNm                                0.85702    0.17002  10.93170
5.041
## donor.populationNC                         -0.09781    0.16823 360.94138  -
0.581
## donor.populationNm                         -0.17035    0.16433 362.88121  -
1.037
## femsize                                     0.49618    0.13542 362.72243
3.664
```

```
## donor.hole.factorHole                      0.75558    0.20971 360.64672  
3.603
## donor.round.adj                            0.39900    0.39573 360.61079  
1.008
## populationNC:donor.hole.factorHole        -0.44629    0.22664 362.30507  -
1.969
## populationNm:donor.hole.factorHole        -0.72729    0.21830 360.97341  -
3.332
## donor.populationNC:donor.hole.factorHole   0.41709    0.23463 360.98364  
1.778
## donor.populationNm:donor.hole.factorHole   0.48189    0.23227 362.89812  
2.075
##                                          Pr(>|t|)    
## (Intercept)                              0.171764    
## populationNC                             0.001130 ** 
## populationNm                             0.000385 ***
## donor.populationNC                       0.561341    
## donor.populationNm                       0.300584    
## femsize                                  0.000285 ***
## donor.hole.factorHole                    0.000359 ***
## donor.round.adj                          0.314002    
## populationNC:donor.hole.factorHole       0.049698 *  
## populationNm:donor.hole.factorHole       0.000953 ***
## donor.populationNC:donor.hole.factorHole 0.076308 .  
## donor.populationNm:donor.hole.factorHole 0.038721 *  
## ---
## Signif. codes:  0 '***' 0.001 '**' 0.01 '*' 0.05 '.' 0.1 ' ' 1
##
## Correlation of Fixed Effects:
##             (Intr) ppltNC ppltnN dnr.NC dnr.pN femsiz dnr..H dnr.r. pNC:..
## populatinNC -0.273                                                        
## populatinNm -0.245  0.499                                                 
## dnr.ppltnNC -0.092  0.088  0.073                                          
## dnr.ppltnNm -0.003  0.028  0.010  0.351                                   
## femsize     -0.884  0.186  0.133  0.067 -0.011                            
## dnr.hl.fctH -0.229  0.378  0.397  0.298  0.267  0.097                     
## donr.rnd.dj -0.441 -0.014  0.013 -0.123 -0.158  0.018  0.052              
## ppltnNC:..H  0.072 -0.705 -0.342 -0.064 -0.027 -0.004 -0.518  0.037       
## ppltnNm:..H  0.109 -0.339 -0.684 -0.061 -0.017 -0.051 -0.534  0.051  0.497
## dnr.pNC:..H  0.138 -0.068 -0.058 -0.707 -0.236 -0.082 -0.574 -0.011  0.023
## dnr.ppN:..H  0.102 -0.025 -0.014 -0.233 -0.685 -0.036 -0.565 -0.034  0.011
##             pN:..H d.NC:.
## populatinNC              
## populatinNm              
## dnr.ppltnNC              
## dnr.ppltnNm              
## femsize                  
## dnr.hl.fctH              
## donr.rnd.dj              
## ppltnNC:..H              
```

```
## ppltnNm:..H
## dnr.pNC:..H  0.020
## dnr.ppN:..H -0.003  0.502

# interaction between population and donor population is also significant
anova(mass.1, mass.1.3)

## Data: dt.nofail
## Models:
## mass.1.3: broodmass ~ population + donor.population + femsize + donor.hole
.factor + population * donor.hole.factor + donor.population * donor.hole.fact
or + donor.round.adj + (1 | pop.block.eff)
## mass.1: broodmass ~ population * donor.population + femsize + donor.hole.f
actor + population * donor.hole.factor + donor.population * donor.hole.factor
+ donor.round.adj + (1 | pop.block.eff)
##          npar    AIC    BIC  logLik deviance Chisq Df Pr(>Chisq)
## mass.1.3   14 975.53 1030.5 -473.76   947.53
## mass.1     18 972.98 1043.7 -468.49   936.98 10.55  4    0.03211 *
## ---
## Signif. codes:  0 '***' 0.001 '**' 0.01 '*' 0.05 '.' 0.1 ' ' 1

mass.1.4 <- lmer(broodmass ~ population * donor.population + femsize + donor.
hole.factor + population * donor.hole.factor + donor.population*donor.hole.fa
ctor + (1|pop.block.eff), data = dt.nofail)
# roundness not signigicant but also removing it doesn't decrease AIC so I'm
keeping it in the model
anova(mass.1, mass.1.4)

## Data: dt.nofail
## Models:
## mass.1.4: broodmass ~ population * donor.population + femsize + donor.hole
.factor + population * donor.hole.factor + donor.population * donor.hole.fact
or + (1 | pop.block.eff)
## mass.1: broodmass ~ population * donor.population + femsize + donor.hole.f
actor + population * donor.hole.factor + donor.population * donor.hole.factor
+ donor.round.adj + (1 | pop.block.eff)
##          npar    AIC    BIC  logLik deviance  Chisq Df Pr(>Chisq)
## mass.1.4   17 972.03 1038.8 -469.01   938.03
## mass.1     18 972.98 1043.7 -468.49   936.98 1.0519  1     0.3051

# mass.1 is minimal adequate model

validatemodel(mass.1, ident = "mass1")

# checking if removing random effect alters model quantitatively
mass.1.4.lm <- lm(broodmass ~ population * donor.population + femsize + donor
.hole.factor + population * donor.hole.factor + donor.population*donor.hole.f
actor, data = dt.nofail)
```

Post-hoc comparisons with emmeans. Results reported in Tables S4, S5 and S6.

```r
masshoc.1 <- emmeans(mass.1, list(pairwise ~ population |  donor.hole.factor
), adjust='tukey')
summary(masshoc.1)

## $`emmeans of population | donor.hole.factor`
## donor.hole.factor = No hole:
##  population emmean    SE    df lower.CL upper.CL
##  FC           1.45 0.125 12.55     1.18     1.72
##  NC           2.22 0.139 19.14     1.93     2.51
##  Nm           2.38 0.121 11.24     2.11     2.64
##
## donor.hole.factor = Hole:
##  population emmean    SE    df lower.CL upper.CL
##  FC           2.56 0.116  9.46     2.30     2.82
##  NC           2.81 0.117  9.69     2.55     3.07
##  Nm           2.62 0.117  9.75     2.36     2.89
##
## Results are averaged over the levels of: donor.population
## Degrees-of-freedom method: satterthwaite
## Confidence level used: 0.95
##
## $`pairwise differences of population | donor.hole.factor`
## donor.hole.factor = No hole:
##  2        estimate    SE    df t.ratio p.value
##  FC - NC   -0.7739 0.187 15.62  -4.143  0.0022
##  FC - Nm   -0.9302 0.173 11.66  -5.376  0.0005
##  NC - Nm   -0.1563 0.183 14.34  -0.855  0.6758
##
## donor.hole.factor = Hole:
##  2        estimate    SE    df t.ratio p.value
##  FC - NC   -0.2499 0.165  9.74  -1.512  0.3276
##  FC - Nm   -0.0611 0.163  9.31  -0.374  0.9265
##  NC - Nm    0.1889 0.163  9.29   1.155  0.5061
##
## Results are averaged over the levels of: donor.population
## Degrees-of-freedom method: satterthwaite
## P value adjustment: tukey method for comparing a family of 3 estimates

masshoc.2 <- emmeans(mass.1, list(pairwise ~ donor.population | population),
adjust='tukey')
summary(masshoc.2)

## $`emmeans of donor.population | population`
## population = FC:
##  donor.population emmean    SE   df lower.CL upper.CL
##  FC                 2.13 0.145 22.8     1.83     2.43
##  NC                 1.93 0.141 20.3     1.63     2.22
##  Nm                 1.96 0.145 22.5     1.66     2.26
##
## population = NC:
```

```
##  donor.population emmean    SE   df lower.CL upper.CL
##  FC                 2.46 0.150 25.6     2.15     2.77
##  NC                 2.74 0.153 27.5     2.43     3.05
##  Nm                 2.35 0.153 27.6     2.04     2.66
##
## population = Nm:
##  donor.population emmean    SE   df lower.CL upper.CL
##  FC                 2.25 0.146 22.9     1.95     2.55
##  NC                 2.54 0.144 21.7     2.24     2.84
##  Nm                 2.71 0.139 19.0     2.42     3.00
##
## Results are averaged over the levels of: donor.hole.factor
## Degrees-of-freedom method: satterthwaite
## Confidence level used: 0.95
##
## $`pairwise differences of donor.population | population`
## population = FC:
##  2       estimate    SE  df t.ratio p.value
##  FC - NC   0.2065 0.194 356   1.064  0.5374
##  FC - Nm   0.1720 0.198 358   0.871  0.6590
##  NC - Nm  -0.0344 0.191 357  -0.180  0.9823
##
## population = NC:
##  2       estimate    SE  df t.ratio p.value
##  FC - NC  -0.2798 0.207 356  -1.349  0.3691
##  FC - Nm   0.1096 0.207 356   0.529  0.8569
##  NC - Nm   0.3894 0.202 356   1.928  0.1323
##
## population = Nm:
##  2       estimate    SE  df t.ratio p.value
##  FC - NC  -0.2885 0.201 357  -1.435  0.3242
##  FC - Nm  -0.4588 0.195 356  -2.352  0.0502
##  NC - Nm  -0.1702 0.189 356  -0.903  0.6389
##
## Results are averaged over the levels of: donor.hole.factor
## Degrees-of-freedom method: satterthwaite
## P value adjustment: tukey method for comparing a family of 3 estimates
```

```
masshoc.3 <- emmeans(mass.1, list(pairwise ~ donor.hole.factor | donor.popula
tion), adjust='tukey')
summary(masshoc.3)
```

```
## $`emmeans of donor.hole.factor | donor.population`
## donor.population = FC:
##  donor.hole.factor emmean     SE    df lower.CL upper.CL
##  No hole             2.11 0.0985  41.2     1.91     2.31
##  Hole                2.45 0.1381 125.3     2.18     2.73
##
## donor.population = NC:
##  donor.hole.factor emmean     SE    df lower.CL upper.CL
```

```
##   No hole              2.03 0.1390 126.1     1.76     2.31
##   Hole                 2.77 0.0969  38.8     2.58     2.97
##
## donor.population = Nm:
##   donor.hole.factor emmean     SE    df lower.CL upper.CL
##   No hole              1.91 0.1343 111.1     1.64     2.17
##   Hole                 2.77 0.0997  43.2     2.57     2.97
##
## Results are averaged over the levels of: population
## Degrees-of-freedom method: satterthwaite
## Confidence level used: 0.95
##
## $`pairwise differences of donor.hole.factor | donor.population`
## donor.population = FC:
##   2               estimate    SE  df t.ratio p.value
##   No hole - Hole   -0.341 0.165 357  -2.058  0.0403
##
## donor.population = NC:
##   2               estimate    SE  df t.ratio p.value
##   No hole - Hole   -0.744 0.167 358  -4.468  <.0001
##
## donor.population = Nm:
##   2               estimate    SE  df t.ratio p.value
##   No hole - Hole   -0.865 0.163 359  -5.313  <.0001
##
## Results are averaged over the levels of: population
## Degrees-of-freedom method: satterthwaite
```

Brood mortality

Analysis of residuals from a regression of brood size on clutch size. Results reported in Table 3, 4, 5, and S8, and Figures 4 and S3.

First we fit the populationar regression, then add the residuals of that regression to the data set.

```
reg1 <- lm(broodsize ~ eggnr, data = dt.nofail)

dt.nofail$reg1.resid <- reg1$res
```

Now we fit a GLMM with residuals from regression of brood size on clutch size as response variable. We perform model selection as in all previous models.

```
# include only data for which there is female size
# to avoid errors in model comparisons (models would be fitted to data sets o
f different sizes)
dt.nofail.2 <- dt.nofail[!is.na(dt.nofail$femsize),]

res.m1 <- lmer(reg1.resid ~ population * donor.population + femsize + donor.h
ole.factor + population * donor.hole.factor + donor.round.adj + (1|pop.block.
```

```
eff), data = dt.nofail.2)
summary(res.m1)
```

```
## Linear mixed model fit by REML. t-tests use Satterthwaite's method [
## lmerModLmerTest]
## Formula:
## reg1.resid ~ population * donor.population + femsize + donor.hole.factor +
##     population * donor.hole.factor + donor.round.adj + (1 | pop.block.eff)
##    Data: dt.nofail.2
##
## REML criterion at convergence: 2522.9
##
## Scaled residuals:
##      Min       1Q   Median       3Q      Max
## -2.51558 -0.61471  0.07705  0.67666  2.55992
##
## Random effects:
##  Groups        Name        Variance Std.Dev.
##  pop.block.eff (Intercept)  1.375   1.173
##  Residual                  55.033   7.418
## Number of obs: 375, groups:  pop.block.eff, 6
##
## Fixed effects:
##                                      Estimate Std. Error       df t value
## (Intercept)                          -12.3293     6.2805 340.9183  -1.963
## populationNC                           4.8778     2.1924  13.7438   2.225
## populationNm                           6.7075     2.0977  11.5834   3.198
## donor.populationNC                    -3.1378     1.6660 358.1308  -1.883
## donor.populationNm                    -3.0481     1.6937 358.8284  -1.800
## femsize                                0.4192     1.1594 359.7658   0.362
## donor.hole.factorHole                 11.8939     1.3753 358.4593   8.648
## donor.round.adj                        3.1922     3.4183 358.3921   0.934
## populationNC:donor.populationNC        4.8472     2.4317 358.1163   1.993
## populationNm:donor.populationNC        2.8261     2.3678 358.3373   1.194
## populationNC:donor.populationNm        2.1588     2.4310 358.4452   0.888
## populationNm:donor.populationNm        4.7212     2.3408 358.5749   2.017
## populationNC:donor.hole.factorHole    -4.6346     2.0298 359.3391  -2.283
## populationNm:donor.hole.factorHole    -6.5479     1.9508 358.7122  -3.356
##                                      Pr(>|t|)
## (Intercept)                          0.050445 .
## populationNC                         0.043373 *
## populationNm                         0.007989 **
## donor.populationNC                   0.060449 .
## donor.populationNm                   0.072752 .
## femsize                              0.717914
## donor.hole.factorHole                 < 2e-16 ***
## donor.round.adj                      0.351004
## populationNC:donor.populationNC      0.046983 *
## populationNm:donor.populationNC      0.233431
## populationNC:donor.populationNm      0.375124
```

```
## populationNm:donor.populationNm    0.044446 *
## populationNC:donor.hole.factorHole 0.022996 *
## populationNm:donor.hole.factorHole 0.000874 ***
## ---
## Signif. codes:  0 '***' 0.001 '**' 0.01 '*' 0.05 '.' 0.1 ' ' 1

res.m1.2 <- lmer(reg1.resid ~ population + donor.population + femsize + donor
.hole.factor + population * donor.hole.factor + donor.round.adj + (1|pop.bloc
k.eff), data = dt.nofail.2)
# interaction population:donor.population marginally significant, its removal
does not improve AIC, so we keep it
anova(res.m1, res.m1.2)

## Data: dt.nofail.2
## Models:
## res.m1.2: reg1.resid ~ population + donor.population + femsize + donor.hol
e.factor + population * donor.hole.factor + donor.round.adj + (1 | pop.block.
eff)
## res.m1: reg1.resid ~ population * donor.population + femsize + donor.hole.
factor + population * donor.hole.factor + donor.round.adj + (1 | pop.block.ef
f)
##          npar    AIC    BIC  logLik deviance  Chisq Df Pr(>Chisq)
## res.m1.2   12 2589.8 2636.9 -1282.9   2565.8
## res.m1     16 2589.5 2652.3 -1278.8   2557.5 8.2582  4    0.08257 .
## ---
## Signif. codes:  0 '***' 0.001 '**' 0.01 '*' 0.05 '.' 0.1 ' ' 1

res.m1.3 <- lmer(reg1.resid ~ population * donor.population + femsize + donor
.hole.factor + population + donor.hole.factor + donor.round.adj + (1|pop.bloc
k.eff), data = dt.nofail.2)
# interaction population:donor.hole.factor significant, much lower AIC when p
resent
anova(res.m1, res.m1.3)

## Data: dt.nofail.2
## Models:
## res.m1.3: reg1.resid ~ population * donor.population + femsize + donor.hol
e.factor + population + donor.hole.factor + donor.round.adj + (1 | pop.block.
eff)
## res.m1: reg1.resid ~ population * donor.population + femsize + donor.hole.
factor + population * donor.hole.factor + donor.round.adj + (1 | pop.block.ef
f)
##          npar    AIC    BIC  logLik deviance  Chisq Df Pr(>Chisq)
## res.m1.3   14 2597.3 2652.2 -1284.6   2569.3
## res.m1     16 2589.5 2652.3 -1278.8   2557.5 11.752  2   0.002807 **
## ---
## Signif. codes:  0 '***' 0.001 '**' 0.01 '*' 0.05 '.' 0.1 ' ' 1

# is female size significant?
res.m1.4 <- lmer(reg1.resid ~ population * donor.population + donor.hole.fact
or + population * donor.hole.factor + donor.round.adj + (1|pop.block.eff), da
```

```
ta = dt.nofail.2)
anova(res.m1,res.m1.4)

## Data: dt.nofail.2
## Models:
## res.m1.4: reg1.resid ~ population * donor.population + donor.hole.factor +
population * donor.hole.factor + donor.round.adj + (1 | pop.block.eff)
## res.m1: reg1.resid ~ population * donor.population + femsize + donor.hole.
factor + population * donor.hole.factor + donor.round.adj + (1 | pop.block.ef
f)
##          npar    AIC    BIC  logLik deviance  Chisq Df Pr(>Chisq)
## res.m1.4   15 2587.6 2646.6 -1278.8   2557.6
## res.m1     16 2589.5 2652.3 -1278.8   2557.5 0.1254  1     0.7233

# no, and removing it reduces AIC by 2. so we can remove it

# is carcass roundness significant?
res.m1.5 <- lmer(reg1.resid ~ population * donor.population + femsize + donor
.hole.factor + population * donor.hole.factor + (1|pop.block.eff), data = dt.
nofail.2)
anova(res.m1, res.m1.5)

## Data: dt.nofail.2
## Models:
## res.m1.5: reg1.resid ~ population * donor.population + femsize + donor.hol
e.factor + population * donor.hole.factor + (1 | pop.block.eff)
## res.m1: reg1.resid ~ population * donor.population + femsize + donor.hole.
factor + population * donor.hole.factor + donor.round.adj + (1 | pop.block.ef
f)
##          npar    AIC    BIC  logLik deviance  Chisq Df Pr(>Chisq)
## res.m1.5   15 2588.5 2647.4 -1279.2   2558.5
## res.m1     16 2589.5 2652.3 -1278.8   2557.5 0.9466  1     0.3306

# no but removing it doesn't improve AIC much (by 1 only)

# removing both female size and carcass roundness
res.m1.6 <- lmer(reg1.resid ~ population * donor.population + donor.hole.fact
or + population * donor.hole.factor + (1|pop.block.eff), data = dt.nofail.2)
anova(res.m1, res.m1.6, test="Chisq")

## Data: dt.nofail.2
## Models:
## res.m1.6: reg1.resid ~ population * donor.population + donor.hole.factor +
population * donor.hole.factor + (1 | pop.block.eff)
## res.m1: reg1.resid ~ population * donor.population + femsize + donor.hole.
factor + population * donor.hole.factor + donor.round.adj + (1 | pop.block.ef
f)
##          npar    AIC    BIC  logLik deviance  Chisq Df Pr(>Chisq)
## res.m1.6   14 2586.6 2641.6 -1279.3   2558.6
## res.m1     16 2589.5 2652.3 -1278.8   2557.5 1.0661  2     0.5868
```

```
anova(res.m1.5, res.m1.6, test="Chisq")

## Data: dt.nofail.2
## Models:
## res.m1.6: reg1.resid ~ population * donor.population + donor.hole.factor +
population * donor.hole.factor + (1 | pop.block.eff)
## res.m1.5: reg1.resid ~ population * donor.population + femsize + donor.hol
e.factor + population * donor.hole.factor + (1 | pop.block.eff)
##          npar    AIC    BIC  logLik deviance  Chisq Df Pr(>Chisq)
## res.m1.6   14 2586.6 2641.6 -1279.3   2558.6
## res.m1.5   15 2588.5 2647.4 -1279.2   2558.5 0.1195  1     0.7296

# this model has lower AIC than res.m1.5 by 2 units, so we continue with res.
m1.6 as minimal adequate model

# checking model residuals
validatemodel(res.m1.6, ident="res.m1.6")

# Checking if presence/absence of random effect changes model quantitatively
res.m1.6.lm <- lm(reg1.resid ~ population * donor.population + donor.hole.fac
tor + population * donor.hole.factor , data = dt.nofail.2)

# significance of interactions in final model
res.m1.6.2 <- lmer(reg1.resid ~ population + donor.population + donor.hole.fa
ctor + population * donor.hole.factor + (1|pop.block.eff), data = dt.nofail.2
)
anova(res.m1.6, res.m1.6.2)

## Data: dt.nofail.2
## Models:
## res.m1.6.2: reg1.resid ~ population + donor.population + donor.hole.factor
+ population * donor.hole.factor + (1 | pop.block.eff)
## res.m1.6: reg1.resid ~ population * donor.population + donor.hole.factor +
population * donor.hole.factor + (1 | pop.block.eff)
##            npar    AIC    BIC  logLik deviance Chisq Df Pr(>Chisq)
## res.m1.6.2   10 2586.8 2626.0 -1283.4   2566.8
## res.m1.6     14 2586.6 2641.6 -1279.3   2558.6 8.192  4    0.08479 .
## ---
## Signif. codes:  0 '***' 0.001 '**' 0.01 '*' 0.05 '.' 0.1 ' ' 1

res.m1.6.3 <- lmer(reg1.resid ~ population * donor.population + donor.hole.fa
ctor + population + donor.hole.factor + (1|pop.block.eff), data = dt.nofail.2
)
anova(res.m1.6, res.m1.6.3)

## Data: dt.nofail.2
## Models:
## res.m1.6.3: reg1.resid ~ population * donor.population + donor.hole.factor
+ population + donor.hole.factor + (1 | pop.block.eff)
## res.m1.6: reg1.resid ~ population * donor.population + donor.hole.factor +
population * donor.hole.factor + (1 | pop.block.eff)
```

```
##              npar    AIC    BIC  logLik deviance  Chisq Df Pr(>Chisq)
## res.m1.6.3    12 2594.8 2641.9 -1285.4   2570.8
## res.m1.6      14 2586.6 2641.6 -1279.3   2558.6 12.172  2   0.002274 **
## ---
## Signif. codes:  0 '***' 0.001 '**' 0.01 '*' 0.05 '.' 0.1 ' ' 1
```

Post-hoc comparisons with emmeans package. Reported in Tables 4, 5 and S8.

```
res.hoc.1 <- emmeans(res.m1.6,  list(pairwise ~ population | donor.population
), adjust='tukey')
summary(res.hoc.1)

## $`emmeans of population | donor.population`
## donor.population = FC:
##  population emmean   SE    df lower.CL upper.CL
##  FC         -1.953 1.44 10.09  -5.1601     1.25
##  NC          0.504 1.47 10.86  -2.7306     3.74
##  Nm          1.269 1.42  9.44  -1.9131     4.45
##
## donor.population = NC:
##  population emmean   SE    df lower.CL upper.CL
##  FC         -5.010 1.42  9.63  -8.1986    -1.82
##  NC          2.291 1.48 11.19  -0.9572     5.54
##  Nm          1.292 1.43  9.72  -1.9002     4.48
##
## donor.population = Nm:
##  population emmean   SE    df lower.CL upper.CL
##  FC         -4.760 1.43  9.83  -7.9562    -1.56
##  NC         -0.166 1.49 11.55  -3.4280     3.10
##  Nm          3.240 1.41  9.15   0.0694     6.41
##
## Results are averaged over the levels of: donor.hole.factor
## Degrees-of-freedom method: satterthwaite
## Confidence level used: 0.95
##
## $`pairwise differences of population | donor.population`
## donor.population = FC:
##  2       estimate   SE    df t.ratio p.value
##  FC - NC   -2.458 2.06 10.47  -1.195  0.4813
##  FC - Nm   -3.222 2.02  9.76  -1.594  0.2932
##  NC - Nm   -0.764 2.04 10.14  -0.375  0.9261
##
## donor.population = NC:
##  2       estimate   SE    df t.ratio p.value
##  FC - NC   -7.301 2.05 10.39  -3.557  0.0125
##  FC - Nm   -6.302 2.02  9.67  -3.126  0.0275
##  NC - Nm    0.999 2.05 10.44   0.486  0.8793
##
## donor.population = Nm:
##  2       estimate   SE    df t.ratio p.value
```

```
##  FC - NC   -4.594 2.07 10.67  -2.223  0.1122
##  FC - Nm   -8.000 2.01  9.48  -3.989  0.0073
##  NC - Nm   -3.406 2.05 10.31  -1.663  0.2647
##
## Results are averaged over the levels of: donor.hole.factor
## Degrees-of-freedom method: satterthwaite
## P value adjustment: tukey method for comparing a family of 3 estimates
```

```
res.hoc.2<- emmeans(res.m1.6,  list(pairwise ~ population | donor.hole.factor
), adjust='tukey')
summary(res.hoc.2)
```

```
## $`emmeans of population | donor.hole.factor`
## donor.hole.factor = No hole:
##  population emmean   SE   df lower.CL upper.CL
##  FC         -9.813 1.30 6.62   -12.91   -6.714
##  NC         -2.686 1.40 9.07    -5.86    0.485
##  Nm         -0.645 1.28 6.33    -3.74    2.450
##
## donor.hole.factor = Hole:
##  population emmean   SE   df lower.CL upper.CL
##  FC          1.997 1.24 5.64    -1.09    5.090
##  NC          4.439 1.24 5.47     1.34    7.534
##  Nm          4.512 1.24 5.60     1.42    7.604
##
## Results are averaged over the levels of: donor.population
## Degrees-of-freedom method: satterthwaite
## Confidence level used: 0.95
##
## $`pairwise differences of population | donor.hole.factor`
## donor.hole.factor = No hole:
##  2        estimate   SE   df t.ratio p.value
##  FC - NC  -7.1265 1.91 7.80  -3.732  0.0148
##  FC - Nm  -9.1679 1.82 6.47  -5.033  0.0046
##  NC - Nm  -2.0414 1.90 7.64  -1.075  0.5558
##
## donor.hole.factor = Hole:
##  2        estimate   SE   df t.ratio p.value
##  FC - NC  -2.4416 1.75 5.55  -1.393  0.4060
##  FC - Nm  -2.5145 1.76 5.62  -1.430  0.3888
##  NC - Nm  -0.0728 1.75 5.54  -0.042  0.9990
##
## Results are averaged over the levels of: donor.population
## Degrees-of-freedom method: satterthwaite
## P value adjustment: tukey method for comparing a family of 3 estimates
```

Figures

The following code will produce figures which were used for the manuscript. Asterisks denoting significance were added in Inkscape and legends were also edited for aesthetics. Loading packages and defining themes to be applied:

```
library(ggplot2) # package to make graphs
library(gridExtra) # package to easily arrange graphs in grid
library(svglite)

mytheme <- theme_classic() + theme(axis.text = element_text(size=16),
                   axis.title=element_text(size=16),
                   #legend.position='none',
                   legend.title = element_text(size=14),
                   legend.text = element_text(size=14))

mytheme_nolegend <- theme_classic() + theme(axis.text = element_text(size=16)
,
                   axis.title=element_text(size=16),
                   legend.position='none')
```

Figure 1: Carcass roundness, interaction with parent size and population of origin

Use the predict function (see ?predict.merMod for more details) to get model predictions with dummy data frames, then plot these with the raw data to show what the model tells us (and check that it is providing a good fit to the data).

```
# Set up dummy data frame for predictions:
# - a sequence of size values
# - all population treatments
# - 'average over' carcass mass
# - 'average over' malesize/femsize

# dummy data frame for female size
df_pred1 <- as.data.frame(expand.grid(femsize = seq(from = min(dt$femsize, na
.rm=T),
                                                    to = max(dt$femsize, na.r
m=T),
                                                    length.out = 450),
                      population = c("FC","NC","Nm"),
                      carc.wt = mean(dt$carc.wt, na.rm=T),
                                     malesize = mean(dt$malesize, na.rm=T)))

# dummy data frame for male size
df_pred2 <- as.data.frame(expand.grid(malesize = seq(from = min(dt$malesize,
na.rm=T),
                                                    to = max(dt$malesize, na.
rm=T),
                                                    length.out = 450),
                      population = c("FC","NC","Nm"),
```

```
                      carc.wt = mean(dt$carc.wt, na.rm=T),
                                      femsize = mean(dt$femsize, na.rm=T)))


# Add fitted value to our data frame by sending this to predict
# - note that we average over random effects with 're.form = NA'
# Note: round2 was the minimal adequate model,
# hence we will use this to get predicted values

df_pred1$fit <- predict(round2, newdata = df_pred1, re.form = NA)
df_pred2$fit <- predict(round2, newdata = df_pred2, re.form = NA)
p.fems <- ggplot(NULL, aes(x = femsize, y=fit))
p.males <- ggplot(NULL, aes(x = malesize, y=fit))

# make sure no NAs present in the dataset
dt.noNAs <- dt[!is.na(dt$femsize) & !is.na(dt$malesize),]

#NOTE: for data points - use shapes from 21 to 25, which have both colour and
fill properties, in order to edit these separately

complete_pfems <- p.fems +
    scale_y_continuous(breaks=c(0.4, 0.6, 0.8, 1.0), limits=c(0.3,1),) +
    scale_x_continuous(breaks=c(3,3.5, 4, 4.5, 5, 5.5, 6)) +
    geom_line(data=df_pred1, aes(linetype=population, colour = population), s
ize = 2) +
    scale_linetype_manual(values=c(1, 2, 3)) +
        scale_color_manual(values=c('red','blue','#30ad0eff')) +
    geom_point(data = dt.noNAs,
             aes(x=femsize,y = roundness.adjust, fill=population),
             size = 2.5, shape=21) +
        scale_color_manual(values=c('red','blue','#30ad0eff')) +
        scale_fill_manual(values=c('red','blue','#30ad0eff'), name='Populatio
n', labels=c('FC', 'NC', 'Nm'))+
    labs(x = "Female pronotum width (mm)",
           y = "Adjusted carcass roundness") +
    mytheme

complete_pmales <- p.males +
    scale_y_continuous(breaks=c(0.4, 0.6, 0.8, 1.0), limits=c(0.3,1), labels=
rep("",4) )+
    scale_x_continuous(breaks=c(3,3.5, 4, 4.5, 5, 5.5, 6)) +
    geom_line(data=df_pred2, aes(linetype=population, colour = population), s
ize = 2) +
    scale_linetype_manual(values=c(1, 2, 3)) +
        scale_color_manual(values=c('red','blue','#30ad0eff')) +
    geom_point(data = dt.noNAs,
             aes(x=malesize,y = roundness.adjust, fill=population),
             size = 2.5, shape=21) +
        scale_color_manual(values=c('red','blue','#30ad0eff')) +
        scale_fill_manual(values=c('red','blue','#30ad0eff')) +
```

```
    labs(x = "Male pronotum width (mm)",
           y = "") +
     mytheme_nolegend

# get legend from complete_pfems
legend <- get_legend(complete_pfems)
#remove legend from plot
complete_pfems <- complete_pfems + theme(legend.position="none")

# use ggsave to directly save a svg file (or any other kind of file)
# grid.arrange() to place the two plots on the same grid
# and add the legend as a third column in the grid
# width and height arguments can be used to change size of image

grid1<- grid.arrange(complete_pfems, complete_pmales, legend, ncol=3, widths=
c(3, 3, 0.8))

ggsave('Fig1_roundness_by_size.svg',grid1,width=10, height=6)
```

Figure 2: brood size and brood mass pop x hole interactions

```
#defining new themes
mytheme_fig2 <- theme_classic() + theme(axis.text = element_text(size=16),
                   axis.title=element_text(size=16),
                   axis.title.x=element_blank(),
                       axis.text.x=element_blank(),
                       axis.ticks.x=element_blank(),
                   panel.background = element_rect(colour = 'black'),
                   legend.position='none')

mytheme_legend_fig2 <- theme_classic() + theme(axis.text = element_text(size=
16),
                   axis.title=element_text(size=16),
                   axis.title.x=element_blank(),
                       axis.text.x=element_blank(),
                       axis.ticks.x=element_blank(),
                   panel.background = element_rect(colour = 'black'),
                   legend.title = element_text(size=18),
                   legend.text = element_text(size=16))

# make a two-panel plot of brood size per population, in carcasses  with/with
out hole
broodsize_all <- ggplot(dt.nofail, aes(y=broodsize, x = population, fill=popu
lation )) +
             geom_boxplot(aes(fill=population), position='dodge2', width = 0.
8)+
                 scale_fill_manual(values=c('red','blue','#30ad0eff'), name=
```

```
'Population') +
                 labs(x='',y='') +
                     facet_wrap(.~ donor.hole.factor) +
                 mytheme_legend_fig2

# make a two-panel plot of brood mass per population, in carcasses  with/with
out hole
broodmass_all <- ggplot(dt.nofail, aes(y=broodmass, x = population, fill=popu
lation )) +
             geom_boxplot(aes(fill=population), position='dodge2', width = 0.
8)+
             scale_fill_manual(values=c('red','blue','#30ad0eff')) +
             labs(x='',y='') +
             facet_wrap(.~ donor.hole.factor) +
             mytheme_fig2

legendfig2 <- get_legend(broodsize_all)

#remove legend from first graph
broodsize_all <- broodsize_all + theme(legend.position="none")
# arrange layout
lay2 <- rbind(c(1,2),c(4,NA))
mygrid2 <- grid.arrange(broodsize_all, legendfig2,
               broodmass_all,
               layout_matrix = lay2, nrow=2, ncol= 2, widths=c(4,1))

ggsave('Fig2_brood_hole.svg', mygrid2, width=7, height=7)
```

Figure 3: brood size and brood mass, interactions between population and origin of carcass

```
broodsize_carcass <- ggplot(dt.nofail, aes(y=broodsize, x = donor.population)
) +
             geom_boxplot(aes(fill=donor.population), position='dodge2', widt
h = 0.8)+
             scale_fill_manual(values=c('red','blue','#30ad0eff'), name='Carc
ass') +
             labs(x='',y='') +
             facet_wrap(.~ population) +
             mytheme_legend_fig2

broodmass_carcass <- ggplot(dt.nofail, aes(y=broodmass, x = donor.population
)) +
             geom_boxplot(aes(fill=donor.population), position='dodge2', widt
h = 0.8)+
             scale_fill_manual(values=c('red','blue','#30ad0eff')) +
             labs(x='',y='') +
             facet_wrap(.~ population) +
```

```
             mytheme_fig2

legendfig3 <- get_legend(broodsize_carcass)
broodsize_carcass <- broodsize_carcass + theme(legend.position="none")
lay <- rbind(c(1,2),c(5,NA))

mygrid1 <- grid.arrange(broodsize_carcass, legendfig3,
               broodmass_carcass,
               layout_matrix = lay, nrow=2, ncol= 2, widths=c(6,1))

ggsave('Fig3_broodsize_mass_carcass.svg', mygrid1, width=10, height=7)
```

Figure 4: Brood mortality, interaction between population and origin of carcass

To make the figure 4, with regressions and residuals boxplots we will do all the different plots and then use grid.arrange to plot in one file. For simplicity we will first divide our dataset in three subsets, one for each carcass origin (FC, NC or Nm).

```
dt.nofail.Fcarc <- dt.nofail[dt.nofail$donor.population == 'FC',]
dt.nofail.Ncarc <- dt.nofail[dt.nofail$donor.population == 'NC',]
dt.nofail.Nmcarc <- dt.nofail[dt.nofail$donor.population == 'Nm',]

# plotting regression of egg number on broodsize
regplot_Fcarc <- ggplot(dt.nofail.Fcarc) +
         geom_point(aes(x=eggnr, y= broodsize, fill=population), size = 4, sh
ape = 21) +
         geom_smooth(data = dt.nofail, aes(x=eggnr, y=broodsize), method=lm,
se=FALSE, fullrange=TRUE, color='black') +
         scale_color_manual(values=c('black','black','black')) +
         scale_fill_manual(values=c('red','blue','#30ad0eff'))+
         xlab("") +
         ylab('Brood size')+
         mytheme

regplot_Ncarc <- ggplot(dt.nofail.Ncarc) +
         geom_point(aes(x=eggnr, y= broodsize, fill=population), size = 4, sh
ape = 21) +
         geom_smooth(data = dt.nofail,aes(x=eggnr, y=broodsize), method=lm, s
e=FALSE, fullrange=TRUE, color='black') +
         scale_color_manual(values=c('black','black','black')) +
         scale_fill_manual(values=c('red','blue','#30ad0eff'))+
         xlab("") +
         ylab("Brood size")+
         mytheme_nolegend

regplot_Nmcarc <- ggplot(dt.nofail.Nmcarc) +
          geom_point(aes(x=eggnr, y= broodsize, fill=population), size = 4, s
hape = 21) +
          geom_smooth(data = dt.nofail,aes(x=eggnr, y=broodsize), method=lm,
```

```
se=FALSE, fullrange=TRUE, color='black') +
          scale_color_manual(values=c('black','black','black')) +
          scale_fill_manual(values=c('red','blue','#30ad0eff'))+
          xlab("Number of eggs") + # last plot must include the x-axis label
              ylab("Brood size")+
          mytheme_nolegend

# plotting residuals in boxplots
resbrood_Fcarc <- ggplot(dt.nofail.Fcarc, aes(y=reg1.resid, x = population, f
ill=population )) +
          geom_boxplot(aes(fill=population), position='dodge2')+
          scale_fill_manual(values=c('red','blue','#30ad0eff'))+
          labs(x='',y='Residual') +
          mytheme_nolegend

resbrood_Ncarc <- ggplot(dt.nofail.Ncarc, aes(y=reg1.resid, x = population, f
ill=population )) +
          geom_boxplot(aes(fill=population), position='dodge2')+
                  scale_fill_manual(values=c('red','blue','#30ad0eff'))+
              labs(x='',y='Residual') +
              mytheme_nolegend

resbrood_Nmcarc <- ggplot(dt.nofail.Nmcarc, aes(y=reg1.resid, x = population,
fill=population )) +
           geom_boxplot(aes(fill=population), position='dodge2')+
                   scale_fill_manual(values=c('red','blue','#30ad0eff'))+
               labs(x='Population',y='Residual') +
               mytheme_nolegend

# get legend from first plot
legendfig4<- get_legend(regplot_Fcarc)
# remove legend from plot itself
regplot_Fcarc <- regplot_Fcarc + theme(legend.position="none")
# arrange layout
lay <- rbind(c(1,1),c(2,3),c(4,5),c(6,7))
mygrid <- grid.arrange( legendfig4, regplot_Fcarc, resbrood_Fcarc, regplot_Nc
arc, resbrood_Ncarc, regplot_Nmcarc, resbrood_Nmcarc, layout_matrix = lay, nr
ow=4, ncol=2, heights = c(4, 6,6,6),widths=c(3,3) )

ggsave('Fig4_residuals.svg', mygrid, width=9, height=12)
```

Figure S2: clutch size by population

```
svg('FigS2_eggs_color_FINAL.svg')
print(
      ggplot(dt, aes(x=population, y = eggnr)) +
          geom_boxplot(aes(color=population, fill =population)) +
```

```
          scale_color_manual(values=c('black','black','black')) +
          scale_fill_manual(values=c('red','blue','#30ad0eff'), name='Populat
ion', labels=c('FC', 'NC', 'Nm') ) +
          labs(x='Population', y='Number of eggs') +
          mytheme
)
dev.off()
```

Figure S3: regression of brood size on clutch size

```
svg('FigS3_residuals_bypop.svg')

regplot_all <- ggplot(dt.nofail) +
         geom_point(aes(x=eggnr, y= broodsize, fill=population), size = 4, sh
ape = 21) +
         geom_smooth(data = dt.nofail, aes(x=eggnr, y=broodsize), method=lm,
se=FALSE, fullrange=TRUE, color='black') +
         scale_color_manual(values=c('black','black','black')) +
         scale_fill_manual(values=c('red','blue','#30ad0eff'), name='Populati
on', labels=c('FC', 'NC', 'Nm'))+
         xlab("Number of eggs") +
         ylab('Brood size')+
         mytheme

print(regplot_all)

dev.off()
```

ImageJ script for image analysis

```
//
//    Title: Mouse ball surface area & volume calculator
//    Author: Jolyon Troscianko
//    Date: 30/04/2012
//
//
//    Description:
//    A photo of a mouse ball must be taken from above and from the side.
//    Photos must be taken from as close to 90 degrees apart as possible.
//    The script uses thresholding (which can be manually adjusted) to separate the mouse ball from the background.
//    Therefore the background should be white, and contrast the black mouse ball. The blue channel is used as this is the darkest
//    The background must be clean - any black dots/dirt can confuse the calculations
//    The script then uses a median filter to remove unwanted artefacts/noise/dirt etc... this can be adjusted
//    Next the number of pixels in each vertical slice across the mouse ball is counted, and the nearest slice in the orthogonal picture is found and counted
//    These are converted to millimeters in height and width at each pixel slice through the mouse
//    The circumference of an ellipse is approximated from width and height using C = PI * (a+b) * (1+(3*pow((a-b)/(a+b),2) / (10+pow(4-(3*pow((a-b)/(a+b),2)),0.5))))
//    The area of each ellipse is calculated as A = PI * a * b
//    The volume of each slice is calculated from the known pixels per millimeter, and summed across all slices
//    The area is the sum of the elliptical prism of each slice, plus the end of each surface
//    This method overestimates surface area due to the steps being counted on both vertical and end surfaces, but this is a systematic overestimation, so relative statistics shouldn't be affected
```

```
//

//    Update: now measures sphericity to avoid above problem of overestimating surface area.

//    Sphericity is taken as the average difference between the area of a circle with a circumference equal to the observed perimeter length of the mouse ball, and the observed area of the mouse ball

//    A sphericity value of 1 would be a perfect circle, lower values indicate worsenning sphericity.




//    topPath=File.openDialog("Select top-down image");     // get file location

//    sidePath=File.openDialog("Select side image");        // get file location


      Dialog.create("Settings");

            Dialog.addMessage("Pixel/distance calibration:");

            Dialog.addNumber("Top-down (pixels per mm)", 27.0636);

            Dialog.addNumber("Side (pixels per mm)", 27.6605);

            Dialog.addMessage("\nImage processing:");

            Dialog.addNumber("Threshold", 70);

            Dialog.addNumber("Smoothing radius (pixels)", 25);

            Dialog.addMessage("\nCamera positioning:");

            Dialog.addCheckbox("Flip image", true);

      Dialog.show();


      topPixelsMm = Dialog.getNumber();

      sidePixelsMm = Dialog.getNumber();;

      thresholdVal = Dialog.getNumber();;;

      smoothingVal= Dialog.getNumber();;;;

      flip = Dialog.getCheckbox();
```

```
// FILE LOCATIONS

      f = File.open(""); // display file open dialog for saving output

      print(f, "topfilename,sidefilename,avg circularity") ;

      topDir=getDirectory("Directory containing TOP images");     // select
working directory
      topFileList=getFileList(topDir);                            // list of
images in directory

      sideDir=getDirectory("Directory containing SIDE images");   // select
working directory
      sideFileList=getFileList(sideDir);                          // list of
images in directory

      if(topFileList.length > sideFileList.length)    // error if there are
unequal numbers of files
            exit("Error: unequal number of files in selected folders");

      if(topFileList.length < sideFileList.length)
            exit("Error: unequal number of files in selected folders");

print("\n________________________________");
print("SETTINGS");
print("     Threshold: " + thresholdVal);
print("     Smoothing Radius: " + smoothingVal);

// START OF BATCH PROCESSING LOOP
```

```
for(z=0; z<topFileList.length; z++1){
      topPath = topDir+topFileList[z];
      sidePath = sideDir+sideFileList[z];


// OPEN & PROCESS TOP IMAGE

      open(topPath);
      run("RGB Stack");
      run("Delete Slice");    // only uses the blue channel (channel with
highest contrast)
      run("Delete Slice");
      //setTool(0);     //Rectangle tool
      //waitForUser("Select the Target", "Draw a box to cover the mouse
ball\nensure no other dark objects are selected");

      //run("Crop");

      if(flip==1);      // if ticked, flips image so that both are the same
way round
      run("Flip Horizontally");

      setThreshold(0, thresholdVal);
      run("Convert to Mask");

      medianScript = "radius="+smoothingVal+" slice";
      run("Median...", medianScript);     // threshold

      w = getWidth();   // image dimensions
```

```
      h = getHeight();

// SELECT CENTRE

      doWand(w/2, h/2, 1, "4-connected");

      List.setMeasurements;   // save measurements of perimeter & area
      topPerim = List.getValue("Perim.");
      topArea = List.getValue("Area");

      //print("Top Perimeter: " + topPerim + " Top Area: " + topArea);

      run("Make Inverse");    // invert selection & delete (gets rid of dirt
spots etc..)
      setForegroundColor(255, 255, 255);
      run("Fill", "slice");
      run("Make Inverse");
      run("To Bounding Box");
      run("Crop");

      w = getWidth();   // image dimensions
      h = getHeight();
      topProfile = newArray(w);


for (x=0; x<w; x++){    // pixel column loop

      topProfile[x] = 0;
```

```
	for (y=0; y<h; y++){	// counts the number of white pixels in each column

		val = getPixel(x,y);
		if(val==255)
			topProfile[x] = topProfile[x] + 1;
	}
}


	topProfileCrop = newArray(0);

for(a=0; a<topProfile.length; a++){
	if(topProfile[a]>0){
		topProfileCrop = Array.concat(topProfileCrop, topProfile[a]);
	}
}

	topProfileMm = newArray(topProfileCrop.length);

for(a=0; a<topProfileCrop.length; a++){
	topProfileMm[a] = topProfileCrop[a] / topPixelsMm;	// make array of millimetre values
//	setResult("Top Pixels", a, topProfileCrop[a]);
//	setResult("Top Millimetres", a, topProfileMm[a]);
}

close();
```

```
// OPEN & PROCESS SIDE IMAGE

      open(sidePath);
      run("RGB Stack");
      run("Delete Slice");    // only uses the blue channel (channel with
highest contrast)
      run("Delete Slice");
      //setTool(0);     //Rectangle tool
      //waitForUser("Select the Target", "Draw a box to cover the mouse
ball\nensure no other dark objects are selected");

      //run("Crop");

      setThreshold(0, thresholdVal);
      run("Convert to Mask");

      medianScript = "radius="+smoothingVal+" slice";
      run("Median...", medianScript);     // threshold

      w = getWidth();   // image dimensions
      h = getHeight();

// SELECT CENTRE

      doWand(w/2, h/2, 1, "4-connected");

      List.setMeasurements;   // save measurements of perimeter & area
      sidePerim = List.getValue("Perim.");
      sideArea = List.getValue("Area");
```

```
	//print("Side Perimeter: " + sidePerim + " Side Area: " + sideArea);


	run("Make Inverse");    // invert selection & delete (gets rid of dirt 
spots etc..)
	setForegroundColor(255, 255, 255);
	run("Fill", "slice");
	run("Make Inverse");
	run("To Bounding Box");
	run("Crop");


	w = getWidth();   // image dimensions
	h = getHeight();
	sideProfile = newArray(w);


for (x=0; x<w; x++){    // pixel column loop


	sideProfile[x] = 0;


	for (y=0; y<h; y++){    // counts the number of white pixels in each 
column


		val = getPixel(x,y);
		if(val==255)
			sideProfile[x] = sideProfile[x] + 1;
	}
}
```

```
	sideProfileCrop = newArray(0);

for(a=0; a<sideProfile.length; a++){
	if(sideProfile[a]>0){
		sideProfileCrop = Array.concat(sideProfileCrop, sideProfile[a]);
	}
}

	sideProfileMm = newArray(sideProfileCrop.length);

for(a=0; a<sideProfileCrop.length; a++){
	sideProfileMm[a] = sideProfileCrop[a] / sidePixelsMm; // make array of
millimetre values
//	setResult("Side Pixels", a, sideProfileCrop[a]);
//	setResult("Side Millimetres", a, sideProfileMm[a]);
}

close();

// CIRCUMFRENCE CALCULATION

	arrayRatios = topProfileMm.length / sideProfileMm.length;

	circumfrenceArray = newArray(sideProfileMm.length);
	areaArray = newArray(sideProfileMm.length);

	for(i=0; i<sideProfileMm.length; i++){

		bLoc = round(arrayRatios*i);
```

```
            b = topProfileMm[bLoc]/2;

            a = sideProfileMm[i]/2;

            circumfrenceArray[i] = PI * (a+b) * (1+(3*pow((a-b)/(a+b),2) /
(10+pow(4-(3*pow((a-b)/(a+b),2)),0.5))));

            areaArray[i] = PI * a * b;

      }


      topLength =  topProfileMm.length / topPixelsMm;

      sideLength =  sideProfileMm.length / sidePixelsMm;

      aveLength = (topLength + sideLength)/2;

      mmPerSlice = aveLength/sideProfileMm.length; // average length of mouse


      VolumeSum = 0;

      AreaSum = 0;


for(a=0; a<sideProfileCrop.length; a++){  // loop to count the area and
volume of each slice

      AreaSum = AreaSum + (circumfrenceArray[a]*mmPerSlice);

      VolumeSum = VolumeSum + (areaArray[a]*mmPerSlice);

//    setResult("Circumfrence", a, circumfrenceArray[a]);

}


      EndArea = 0;


for(a=0; a<sideProfileCrop.length-1; a++)

      EndArea = EndArea + (pow(pow(areaArray[a]-areaArray[a+1],2),0.5)); //
sums end surface values - always positive


      AreaSum = AreaSum + EndArea;
```

```
// SPHERICITY CALCULATION - this takes the average value of the area to circumference ratio of the top & side images

	topCircleArea = PI*pow((topPerim/(2*PI)),2);	// this calculates the area a circle would have of the observed perimeter length
	sideCircleArea = PI*pow((sidePerim/(2*PI)),2);

	topRoundness =  topArea / topCircleArea;  // perfect circle area /observed area, so a perfect circle would be 1, going down to zero with worse roundness
	sideRoundness = sideArea / sideCircleArea;

	sphericity = (topRoundness+sideRoundness)/2;	// average roundness

// PRINT RESULTS

print("\n..........................................................");
print("Top-down image: " + topFileList[z]);
print("Side-on image: " + sideFileList[z]);
print("Sphericity:\t"+ sphericity);
print("Volume (mm^3):\t"+VolumeSum);

print(f, topFileList[z] + "," + sideFileList[z] + "," + sphericity);

} //end batch processing loop

File.close(f);
```
